# Sobremesa L-type Amino Acid Transporter Expressed in Glia Is Essential for Proper Timing of Development and Brain Growth

**DOI:** 10.1016/j.celrep.2018.08.067

**Published:** 2018-09-18

**Authors:** Diego Galagovsky, Ana Depetris-Chauvin, Gérard Manière, Flore Geillon, Martine Berthelot-Grosjean, Elodie Noirot, Georges Alves, Yael Grosjean

**Affiliations:** 1 Centre des Sciences du Goût et de l’Alimentation, AgroSup Dijon, CNRS, INRA, Université Bourgogne Franche-Comté, 21000 Dijon, France; 2 Plateforme DImaCell, INRA, Université Bourgogne Franche-Comté, 21000 Dijon, France; 3 Department of Evolutionary Neuroethology, Max Planck Institute for Chemical Ecology, 07745 Jena, Germany

**Keywords:** amino acid transporter, glia, nervous system, tissue growth, development, insulin, PTTH, ecdysone, feeding, *Drosophila*

## Abstract

In *Drosophila*, ecdysone hormone levels determine the timing of larval development. Its production is regulated by the stereotypical rise in prothoracicotropic hormone (PTTH) levels. Additionally, ecdysone levels can also be modulated by nutrition (specifically by amino acids) through their action on *Drosophila* insulin-like peptides (Dilps). Moreover, in glia, amino-acid-sensitive production of Dilps regulates brain development. In this work, we describe the function of an SLC7 amino acid transporter, Sobremesa (Sbm). Larvae with reduced Sbm levels in glia remain in third instar for an additional 24 hr. These larvae show reduced brain growth with increased body size but do not show reduction in insulin signaling or production. Interestingly, Sbm downregulation in glia leads to reduced Ecdysone production and a surprising delay in the rise of PTTH levels. Our work highlights Sbm as a modulator of both brain development and the timing of larval development via an amino-acid-sensitive and Dilp-independent function of glia.

## Highlights

• Glia express the SLC7 amino acid transporter Sobremesa, which controls development
• Sobremesa downregulation in glia leads to contrasting effects: small brain and big body size
• Sobremesa downregulation results in reduced ecdysone production
• Sobremesa downregulation causes a delayed rise in PTTH

## Graphical Abstract

**Figure:**
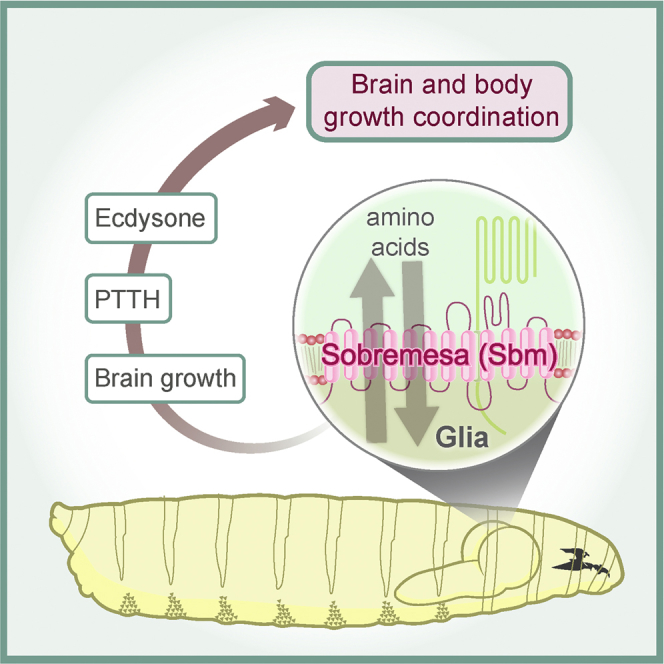

## Teaser

Galagovsky et al. reveal a molecular and cellular pathway linking metabolism to developmental growth control. This pathway governs body and nervous system growth timing and involves an amino acid transporter expressed in glia.

## Introduction

Members of the SLC7 family of proteins are membrane-bound transporters dedicated to the passage of amino acids. They are classified into two subfamilies: cationic amino acid transporters (CATs), facilitating diffusion of cationic amino acids, and L-type amino acid transporters (LATs), which are mostly specific exchangers. CATs act as single molecules, while LATs form heterodimeric amino acid transporters (HATs) together with members of the SLC3 family of proteins. The HAT is formed through a disulfide bridge between the LAT (12 transmembrane domain light subunit) and the SLC3 subunit (heavy subunit), involving a cysteine positioned in the external loop between the III and IV transmembrane domains of the LAT (Fotiadis et al., 2013, Verrey et al., 2004). Based on the structural characteristics of these proteins, bioinformatics analysis of the *Drosophila melanogaster* genome identified five genes that would code for proteins with LAT characteristics, namely *genderblind*, *minidiscs*, *JhI-21*, *CG1607*, and *CG9413* (Augustin et al., 2007, Reynolds et al., 2009). While the expression and function of a *D. melanogaster* CAT transporter Slimfast has been extensively studied (Bjordal et al., 2014, Cheng et al., 2011, Colombani et al., 2003, Okamoto and Nishimura, 2015, Shim et al., 2012), LATs have been a subject of less scrutiny, and not all five LATs have been functionally characterized. In the present study, we focus on CG9413, a yet-undescribed putative *Drosophila* LAT.

SLC7 amino acid transporters can have a potentially crucial role impacting the physiology of an organism. They can serve the uptake of amino acids to directly feed the protein synthesis machinery or impact the reuptake of amino acids in excretory organs such as the gut and kidneys (Palacín et al., 2005). Related to this, they can feed amino acids into signaling pathways that control the production, release, or response to hormones that regulate amino-acid-sensitive processes such as growth, developmental timing, or feeding, thus functioning as amino acid sensors (Fotiadis et al., 2013, Miguel-Aliaga, 2012).

Through amino acid sensors, the nutritional value of food is coupled to the hormonal signals that coordinate growth and developmental timing. In *Drosophila*, insulin-like peptides (Dilps) activate the insulin-like signaling (IIS) pathway in target tissues and induce cellular growth and proliferation (Edgar, 2006, Mirth and Riddiford, 2007). Their production and release depends on both local and remote signals from nutrient sensors that assess the nutritional status of the organism (Delanoue et al., 2016, Géminard et al., 2009, Okamoto and Nishimura, 2015, Rajan and Perrimon, 2012). Developmental timing, determined by rises of the molting hormone ecdysone and the stereotyped rise in the prothoracicotropic hormone (PTTH) (McBrayer et al., 2007), is also coupled to nutrition through Dilp signaling (Colombani et al., 2005, Mirth et al., 2005). CAT Slimfast functions as a general amino acid transporter and, in that capacity, as a nutrient sensor (Colombani et al., 2003). It is expressed in important organs for growth control, such as in fat body and the CNS, and feeds amino acids into the signaling pathways that ultimately impact the production and release of Dilps (Bjordal et al., 2014, Cheng et al., 2011, Colombani et al., 2003, Okamoto and Nishimura, 2015). Interestingly, LAT-1 Minidiscs and JhI-21 have recently been shown to function as leucine sensors in the insulin-producing cells of the larva, thus having a role in the regulation of growth and metabolism (Manière et al., 2016, Ziegler et al., 2018).

In addition to their role as amino acid sensors, SLC7 transporters can also impact neurotransmission, since they can serve in the maintenance of the concentrations of specific amino acids that function as neurotransmitters or as precursors for neurotransmitters (Featherstone, 2011). In the case of LATs, Genderblind is expressed in some glia surrounding glutamatergic neurons, where it regulates the level of extracellular glutamate (Augustin et al., 2007, Piyankarage et al., 2008). Furthermore, Genderblind and also JhI-21 control ionotropic glutamate receptor (iGluR) clustering at the neuromuscular junction (NMJ) (Augustin et al., 2007, Ziegler et al., 2016), implicating an essential role for both LATs in neurotransmission. Moreover, *genderblind* disruption in adults impacts pheromonal perception in males and food odor detection in both sexes (Grosjean et al., 2008).

In the present study, we focus on CG9413, one of the less-studied *Drosophila* putative LATs. We show evidence supporting its capacity to form a HAT together with the *Drosophila* SLC3 protein CD98hc. We confirm that it is expressed in the gut and in the CNS of larvae. Moreover, we reveal that expression of CG9413, here named *sobremesa* (*sbm*), is crucial for proper larval growth, feeding, and timing of pupation. Most importantly, *sbm* expression in glia is a key factor for proper timing of development and brain growth. Sbm glial knockdown provokes a reduction in PTTH somatic levels, revealing an unknown glial function and a possible axis of amino-acid-sensitive communication between glia and PTTH-producing cells.

## Results

### Sobremesa (CG9413 - Sbm) Shows Characteristics of an SLC7 Amino Acid Transporter

**Figure 1:**
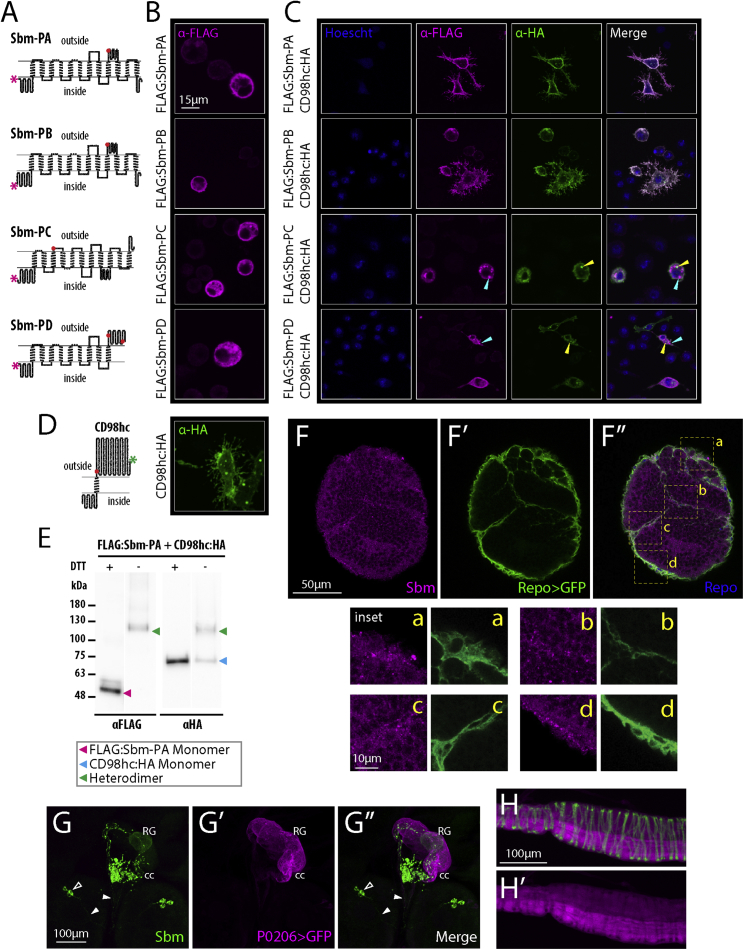
Cellular Localization, Interaction with CD98hc, and Expression Pattern of *sobremesa* (CG9413 – *sbm*) (A) Putative secondary structure of the translation products of each *sbm* mRNA. The magenta asterisk shows the position of the FLAG tag. Each circle represents an amino acid, and red circles represent extracellular cysteine. (B) Expression of each FLAG-tagged protein in S2 cells revealed by staining against FLAG (magenta). PA and PB, but not PC and PD, localize in part to the plasma membrane, and PC and PD form clumps inside the cell. (C) Coexpression of each FLAG:Sbm protein with CD98hc:HA (blue, Hoescht; magenta, FLAG; green, HA) in S2 cells. PA and PB, but not PC and PD, colocalize with CD98hc:HA (cyan and yellow arrowheads for FLAG and HA signal, respectively). PC and PD alter CD98hc localization and abolish the membrane extensions phenotype provoked by CD98hc expression. (D) Putative structure of CD98hc (left) and immunostaining of S2 cells expressing CD98hc:HA depicting the membrane extension phenotype (right). The green asterisk shows the position of the HA tag on CD98hc. (E) Western blot of coexpressing FLAG:Sbm-PA and CD98hc:HA cell extracts in the presence (+) and absence (−) of reducing agent (DTT), revealed with anti-FLAG and anti-HA antibodies. The expected position of the heterodimer and monomer bands are pointed based on the molecular weight marker. Heterodimer bands are revealed in the absence of DTT and disappear with DTT, while monomer bands behave in the opposite way (expected molecular weights: Sbm-PA, 57.6 kDA; CD98hc, 62.7 kDa). (F) Immunostaining in larval brains of Repo-Gal4 > UAS-CD8:GFP reveals expression of Sbm in glia, apparently on the membrane of the subperineurial and cortex glia (Sbm, magenta; GFP, green; Repo, blue; in F″, yellow letters and broken line squares signal the corresponding positions of insets a–d). (G) Sbm (green) is expressed in a group of neurons (somas, hollow arrowhead; projections, filled arrowheads) projecting to the corpora cardiaca (cc) portion of the ring gland (RG). Counterstaining against GFP (magenta) in a P0206-Gal4 > UAS-CD8:GFP line delimits the RG. (H) Immunostaining against Sbm (magenta) shows the expression of Sbm in part of the hindgut; counterstaining with phalloidin (green) is shown to delineate the tissue.

To study *sbm* gene expression in *D. melanogaster*, we performed diagnostic PCRs over total cDNA from *w*^*1118*^ control flies (Figure S1A), followed by cloning and sequencing of CDSs. We confirmed the existence and predicted sequences listed in FlyBase of its 4 transcripts: *sbm*-RA, RB, RC, and RD. Sbm-PA and PB would have a structure that could interact with the putative *Drosophila* SLC3 protein, the HAT heavy subunit CD98hc. On the contrary, Sbm-PC and PD would be shorter proteins with atypical cysteine locations possibly not compatible for this interaction (Figure 1A).

To visualize these possible physical interactions, we made tagged versions of the different isoforms. CD98hc fused to hemagglutinin (CD98hc:HA) expressed alone in S2 cells appears localized in part to the plasma membrane and provokes membrane extensions resembling filopodia and lamelipodia (Figures 1D and S1C). FLAG:Sbm PA and PB expressed alone also show this localization, while PC and PD localize mainly in big clumps inside the cell or near the cell surface. None of the FLAG:Sbm variants provoke any change in the normal morphology of S2 cells (Figure 1B). Co-expression of CD98hc:HA with either FLAG:Sbm PA or PB showed colocalization of FLAG:Sbm variants with CD98hc:HA at the plasma membrane, supporting the hypothetical interaction between the two proteins (Figure 1C). Western blots revealed either with FLAG or HA antibodies show a band at the expected size for the heterodimer. Breaking the disulfide bridges by adding a reducing agent (DTT) effectively abolished the heterodimer band, displaying clear bands corresponding to the monomers (Figures 1E and S1B). On the contrary, co-expression of PC or PD FLAG:Sbm and CD98hc:HA variants leads to a localization in dots inside the cell with no visible colocalization of these 2 subunits and does not affect cell morphology (Figure 1C). Furthermore, FLAG:Sbm-PC and PD do not produce the dimer size bands expected when co-expressed with CD98hc:HA (Figure S1B). These results strongly suggest that Sbm-PA and PB are able to interact with CD98hc to form a heterodimer at the plasma membrane, while the shorter proteins (PC and PD) would not form a heterodimer with CD98hc and may even interfere with its localization. The interaction between Sbm and CD98hc constitutes important evidence to confirm Sbm as an SLC7A family member and also that they form a complex, which potentially functions as a HAT to transport amino acids.

### *sbm* Expression Pattern

To explore *sbm* expression pattern in further detail at the larval stage, we constructed a Sbm-Gal4 line and produced an antibody that would detect all Sbm proteins. *sbm* is expressed in the CNS, both in glia and neurons (Figures 1F, 1G, and S1D–S1G), and the gut (Figures 1H and S1H). Distinct signal can be seen in subperineurial and cortex glia (Figures 1F, S2A, and S2B), but not in other glial subtypes (Figures S2C–S2F), and in a cluster of four neurons in each brain hemisphere that project to the corpora cardiaca in the ring gland (Figures 1G and S1D–S1F). The expression pattern observed with an antibody against Sbm is supported by that observed with the Gal4 line (Figures S1F–S1H). Noteworthy, the antibody signal appears localized in part to the membrane of the cells (Figures 1F, 1G, S2A, and S2B), supporting the predicted structure and function of the protein as a membrane transporter.

### Downregulation of *sbm* in Glia Delays Development and Affects Growth Differentially Among Organs

**Figure 2:**
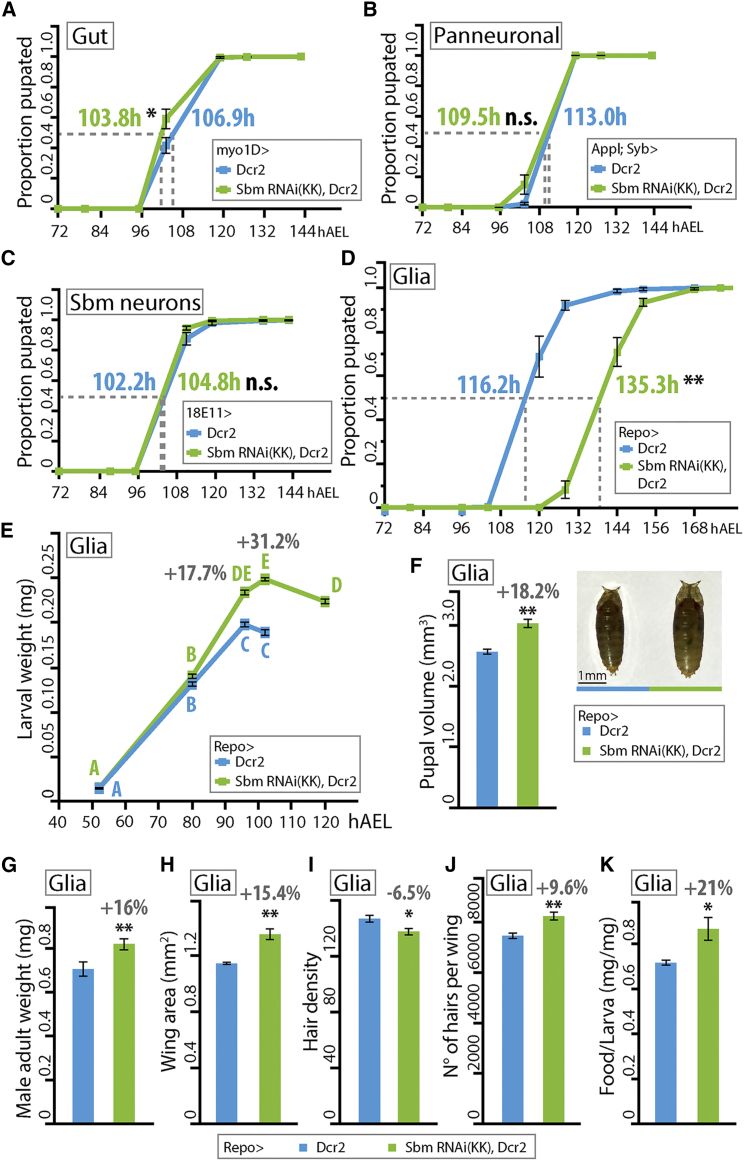
*sbm* Downregulation in Glia Has a Specific Effect on Developmental Timing and Growth (A–C) Proportion of larvae pupated through time after downregulation of *sbm* in the gut (A; myo1D-Gal4), panneuronally (B; Appl-Gal4; Syb-Gal4), and in the group of neurons that project to the corpora cardiaca (C; 18E11-Gal4). In all cases, a t test was performed, and n.s. indicates nonsignificant differences. t values are as follows: t = 2.56 (A, n = 8), t = 1.95 (B, n = 9), and t = 1.79 (C, n = 5). (D–K) Downregulation of *sbm* in glia (via expression of the RNAi with Repo-Gal4) leads to a delay to pupation, an increase in final size, and an increase in feeding. (D) Proportion of larvae pupated through time (t test, t = 8.07; ^∗∗^p < 0.0001; n = 15). (E) Larval weight (in milligrams) at different time points through development (two-way ANOVA; data were transformed to natural logarithm [ln] to fulfill the test criteria; p < 0.0001, F = 18.99; points with different letters signify that they are significantly different with p < 0.05 in a Tukey post-test; n = 15–22 per age and genotype). (F) Estimated pupal volume (in cubic centimeters) (t test, t = 6.09; ^∗∗^p < 0.0001; n = 15). The picture on the right shows example individuals of each genotype. (G) Adult male fly weight (mg) (t test, t = 7.32; ^∗∗^p < 0.0001; n = 36–65). (H) Male fly wing area (mm^2^) (t test, t = 5.51; ^∗∗^p < 0.01; n = 3). (I) Hair density on male fly wings (t test, t = 2.63; ^∗^p < 0.05; n = 3). (J) Estimated total number of hairs per male wing (t test, t = 4.03; ^∗∗^p < 0.01; n = 3). (K) Amount of food (mg) ingested by 22-hr-old synchronized third-instar larvae normalized by their mass (mg) (t test, t = 3.28; ^∗^p < 0.05; n = 5). In all graphs, data represent mean ± SEM, and the percentages above the bars indicate the difference in respect to the control. hAEL, hours after egg laying.

Since *sbm* is expressed in several larval organs and cell types, we next screened for the effects of *sbm* downregulation in these restricted expression domains. To that end, we drove expression of RNAi in the gut using the myo1D-Gal4 driver line (Figure 2A), panneuronally by combining Appl-Gal4 and Syb-Gal4 lines (Figure 2B) or specifically in the Sbm immunoreactive cluster of neurons that project to the corpora cardiaca with the 18E11-Gal4 line (Figure 2C), and finally in glia with Repo-Gal4 line (Sepp et al., 2001, Yuasa et al., 2003) (Figure 2D). Downregulation with two independent RNAi lines strongly reduces *sbm* mRNA levels to the same extent (Figures S4A and S4B) and significantly decreases the protein levels exclusively in the cellular type where the expression is directed (Figures S3A–S3D). Notoriously, specific downregulation of *sbm* with these two independent RNAi lines in glia provoked a strong developmental delay of almost 24 hr (Figures 2D, S4D, and S5D), while the downregulation of *sbm* on any of the other expression domains did not affect developmental timing (Figures 2A–2C). This phenotype led us to name this gene *sbm*, a Spanish word that translates as “the extended period of time spent at the table conversing long after the meal has actually ended.” This developmental delay was specific to the third larval instar, since molting from the second to the third instar was not delayed (Figure S4C). Downregulation of Sbm using specific glial subtype drivers failed to reproduce the developmental delay phenotype observed with the general glial driver Repo-Gal4 (Figure S5A), suggesting that *sbm* function could be important in several glial subtypes and that its downregulation in one could be compensated by others. It is interesting to note that a very strong ubiquitous knockdown of the gene with the Tubulin-Gal4 driver provokes an extended third larval instar in which larvae remain in the feeding substrate for up to 10 days and never commence wandering or pupation (Figures S5B and S5C); this suggests that Sbm could have a function in other tissues apart from glia and the CNS.

Surprisingly, despite the developmental delay in Repo > SbmRNAi flies, larval weight was notably increased, especially at 96 hr after egg laying (hAEL), a time corresponding to the last 24 hr of the third larval instar of controls (Figure 2E), resulting in individuals of bigger final size. This was also attested by an increase in pupal volume (Figures 2F and S4E) and adult male weight (Figure 2G), which also had bigger wings composed of bigger and more numerous cells (Figures 2H–2J). Also noteworthy is that food intake and the rate of food incorporation to body mass was increased in Repo > SbmRNAi flies (Figure 2K.) Contrary to the glial knockdown of Sbm, its overexpression does not lead to a very significant developmental delay and has a negative effect on growth (Figures S5D and S5E).

**Figure 3:**
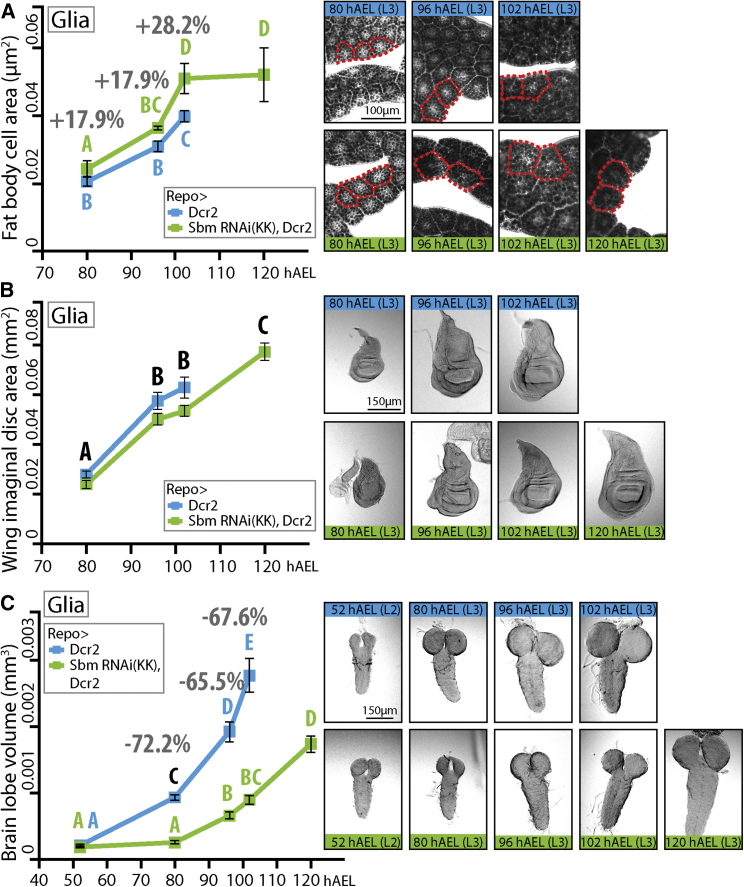
Effects of *sbm* Downregulation in Glia on Various Tissue Growth (A) Fat body cell area (μm^2^) through time and representative pictures of fat bodies stained with Sudan black to show fat accumulation (the red broken line exemplifies some measured cells in each picture) (two-way ANOVA and Duncan post-test, p < 0.01, F = 6.26; n = 4–5.) (B) Wing imaginal disc area (mm^2^) through time and representative pictures (Two-Way ANOVA with a Duncan Post-Test, interaction between the variables time and genotype is not significant so only statistical differences between time points is shown in the graph, p < 0.0001, F = 123.13; n = 11–15). (C) Estimation of larval single brain lobe volume (mm^3^) through time and representative pictures (two-way ANOVA with a Tukey post-test, p < 0.0001, F = 22.55; DMS = 0.34; n = 4–5.) In all statistical analyses, data were transformed to natural logarithm (ln) to fulfill the test criteria. In all graphs, points with different letters signify that they are significantly different (p < 0.05). Data represent mean ± SEM; the percentages above points indicate the difference in respect to the control. hAEL, hours after egg laying.

**Figure 4:**
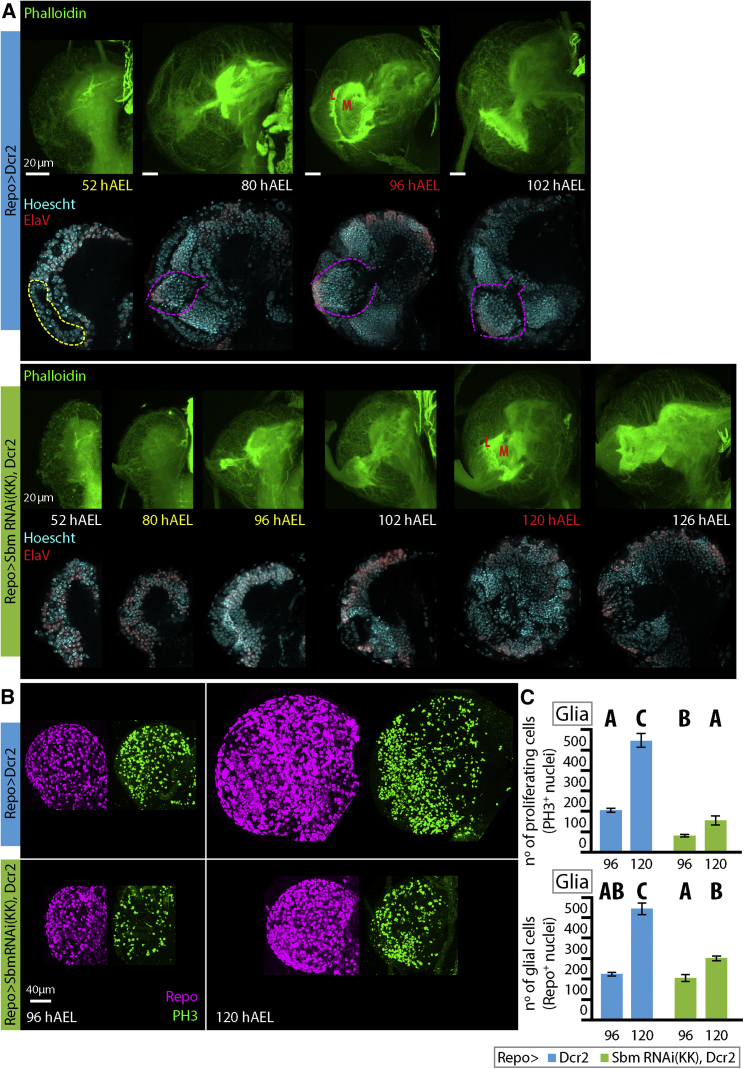
Effects of Sbm Downregulation in Glia on Larval Brain Size, Development, and Brain Cell Proliferation (A) Observation of the formation of the optic lobe and its neuropils by staining brains with phalloidin (green, cell membranes) and Hoescht (blue, nuclei) and neurons (ElaV, red) at different time points through larval development (hAEL, hours after egg laying) shows that Sbm downregulation in glia produces a delay in the development of larval brain structures. In control brains (top two rows), a group of ElaV-negative (red) cells is observed at 52 hAEL (region marked by broken yellow line) in glia Sbm RNAi-treated larvae (bottom two rows); this is not observed earlier than the mid-third-instar larval stage (80–96 hAEL). In control brains, optic lobe formation is already evident by the early third-instar (80 hAEL, broken yellow lines), and the optic lobe (L) and medulla (M) neuropils are already formed in mid-third-instar larvae (96 hAEL). In Sbm RNAi-treated larvae, optic lobe formation seems to occur between 96 and 102 hAEL, and neuropils are seen at 120 hAEL. It is important to note that while structures form, they do not seem to have the regularity observed in controls. (B) Representative pictures showing proliferating cells (PH3, green) and glial cells (Repo, magenta) in the brain of control Repo > Dcr2 (top row) and Repo > SbmRNAi Dcr2 (bottom row) larvae at 96 and 120 hAEL. (C) Quantification of the number of proliferating cells (top) and the number of glial cells (bottom) (two-way ANOVA and Duncan post-test, p < 0.0001, F = 34.33 and F = 37.96; n = 3–6). Points with different letters signify that they are significantly different (p < 0.05); data represent mean ± SEM. hAEL, hours after egg laying.

In order to study if the increased larval weight was a consequence of a general elevated growth rate, we analyzed the size of different organs. We found that not all organs present the same growth rate nor final size differences between controls and Repo > SbmRNAi larvae as the whole animals do. In the case of fat body, an endoreplicative tissue, the cell area behaved similarly to the whole larva, with bigger cells during the early parts of the third larval instar (80 and 96 hAEL), a marked increase in size in the Repo > SbmRNAi larvae relative to controls in the last hours (102 hAEL), and no further rise during the extra time before pupation (120 hAEL) (Figure 3 A). Conversely, the wing imaginal disc area was always significantly smaller in RNAi-treated larvae (a diploid tissue) than in controls, though growth rates were similar (80–102 hAEL). Nevertheless, since the growth rate was maintained during the extra hours they spend as larvae, the final size of the imaginal disc is bigger (120 hAEL), which could in turn account for the bigger wings measured in adults (Figures 3B and 2H). Finally, to our surprise, we observed that the brains of Repo > SbmRNAi larvae were much smaller than that of controls. Even though second-instar larvae do not show statistically significant differences in estimated brain lobe volume (52 hAEL) compared to controls, by the first day of the larval third-instar, their brain size has not changed, much resulting in a brain that is 72.2% smaller than that of controls (80 hAEL). This difference in growth rate is further maintained though time (96 and 102 hAEL), and while Repo > SbmRNAi larvae spend almost 24 extra hours eating and growing (120 hAEL), their brain never reaches the final control size (Figures 3C and S6A). Observations of the anatomical features (Dearborn and Kunes, 2004, Ngo et al., 2017) of the brain of the Sbm-knockdown larvae through these time points show that brain development is delayed compared to controls (Figure 4A). This is also highlighted by the differences in the number of dividing cells. We found that Repo > SbmRNAi larvae have significantly fewer proliferating cells than controls at both 96 and 120 hAEL. This has negative consequences, at least on the number of glial cells, which are significantly less numerous in Repo > SbmRNAi larvae at 120 hAEL, but not at 96 hAEL (Figures 4B and 4C).

To sum up, reduced levels of Sbm in glia produce a developmental delay that results in heavier larvae, bigger wing imaginal discs, and bigger fat body cells but a smaller brain, possibly due to a decrease in cell proliferation with delayed consequences on neuron and glial cell numbers. Extension of the larval stage is accompanied by larvae staying longer in the food, resulting in bigger pupae and bigger and heavier adult flies.

### Downregulation of *sbm* in Dilp6-Expressing Glia Leads to Developmental Delay, but General Glial Knockdown Does Not Lead to Expected Effects of Dilp6 Deficiency

Larval growth is primarily regulated by eight insulin-like peptides (Dilp1–8). Since they all act through a single insulin receptor, regulation of growth is achieved through the spatial, temporal, and circumstantial regulation of their production and release. One such peptide, Dilp6, is produced by glial cells in a nutrient-sensitive manner and regulates brain growth (Okamoto and Nishimura, 2015, Sousa-Nunes et al., 2011).

**Figure 5:**
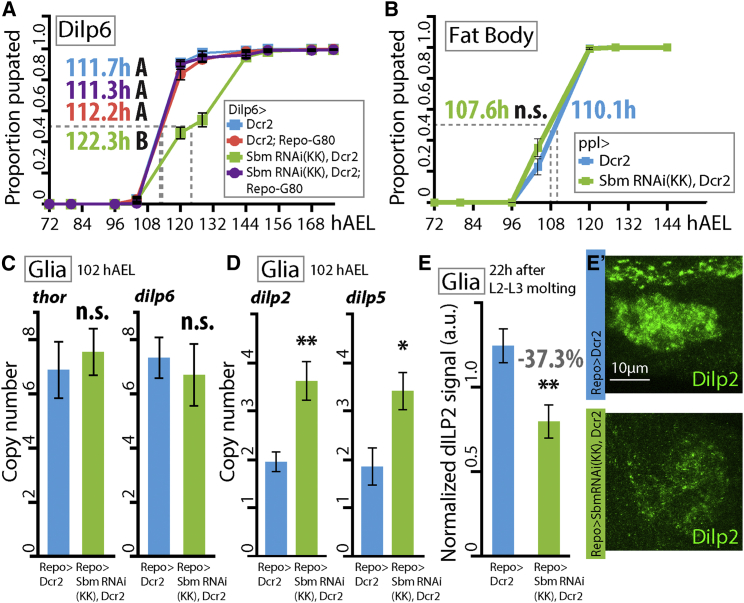
Downregulation of *sbm* in *dilp6*-Expressing Glia Partially Reproduces the Developmental Delay Observed without Affecting *dilp6* Levels (A) Proportion of larvae pupated through time when overexpressing Dcr2 or SbmRNAi, Dcr2 in *dilp6*-expressing cells (Dilp6-Gal4) either alone or combined with Repo-Gal80 (one-way ANOVA; data were transformed to the reciprocal values to best fulfill the test criteria, p < 0.0001, F = 15.79; points with different letters signify that they are significantly different [p < 0.05] with Duncan post-test; n = 4–10). (B) Idem A but for downregulation of *sbm* in the fat body (ppl-Gal4) (t-Test, t = 1.29; n = 4). (C and D) Transcription levels of *thor* and *dilp6* (C) and *dilp2* and *dilp5* (D) (expressed in copy number) in the brain of 102 hAEL third-instar larvae after downregulation of *sbm* in all glia (Repo-Gal4) (t test, *thor* t = 0.57, *dilp6* t = 0.54, *dilp2* t = 4.25, and *dilp5* t = 3.25; ^∗^p < 0.05; ^∗∗^p < 0.001; n = 4). (E) Dilp2 immunoreactive signal levels in the somas of insulin-producing cells (IPCs) 22 hr after the second- to third-instar molt in control and Repo > SbmRNAi larvae (t test, t = 3.27; ^∗∗^p < 0.001; n = 28). The percentage in the graph indicates the difference with respect to controls. (E′) Representative z stack images of each genotype that illustrate the differences in Dilp2 levels. In all graphs, error bars represent SEM and n.s. indicates nonsignificant differences.

This led us to explore if the downregulation of *sbm* in Dilp6-producing glia could reproduce the developmental-delay phenotype observed with the panglial driver Repo. Expression of Sbm RNAi with a Dilp6-Gal4 line (which drives expression in glial cells of the subperineurial glia and others [possibly the cortex glia (Okamoto and Nishimura, 2015) and fat body]) significantly delayed the onset of pupation by ∼11 hr (Figure 5A), a slighter effect than with the general glial driver. This delay was fully rescued when Gal4 activity was blocked in glial cells co-expressing Gal80, indicating that the observed developmental delay in Dilp6 > SbmRNAi larvae depends on deregulation of Sbm in Dilp6+ glial cells. (Figure 5A). Plus, larvae expressing the RNAi in the fat body did not display a pupation time statistically different from controls (Figure 5B).

Amino acids enter glial cells via CAT transporter Slif, which functions as an amino acid sensor, activating the TOR signaling pathway and positively regulating *dilp6* expression (Okamoto and Nishimura, 2015, Sousa-Nunes et al., 2011). If Sbm also functions as an amino acid sensor, then its downregulation should produce effects similar to those caused by a the lack of Slif (i.e., decreased *dilp6* expression and reduced activation of the IIS pathway [target of Dilp6] in the whole brain and reduced production of Dilp5 in insulin-producing cells [IPCs] of the brain; Okamoto and Nishimura, 2015). Contrary to this prediction, the expression level of *dilp6* or the direct target of the IIS pathway, *thor*, were not affected in Repo > SbmRNAi larvae, meaning that the pathway is equally active in the brain of RNAi-treated larvae and controls (Figure 5C). Moreover, the mRNA levels of the two Dilps produced in IPCs, *dilp5* and *dilp2*, were almost doubled in the Repo > SbmRNAi larvae (Figure 5D). It has been described that levels of the Dilp2 immunoreactive signal are inversely correlated with its release into the circulation from the producing cells (Géminard et al., 2009). We found that Repo > SbmRNAi larvae show a lower immunoreactive Dilp2 signal in IPCs (Figure 5E), suggesting that the release of this peptide is augmented in Repo > SbmRNAi larvae.

In conclusion, Sbm functions in Dilp6+ glia, but a reduction of Sbm levels in all glia does not act negatively on *dilp6* expression and function. This suggests that Sbm in glial cells could regulate developmental timing and growth through a different effector.

### *sbm* Downregulation in Glia Reduces the Levels of Key Hormones that Control Developmental Timing and Growth

**Figure 6:**
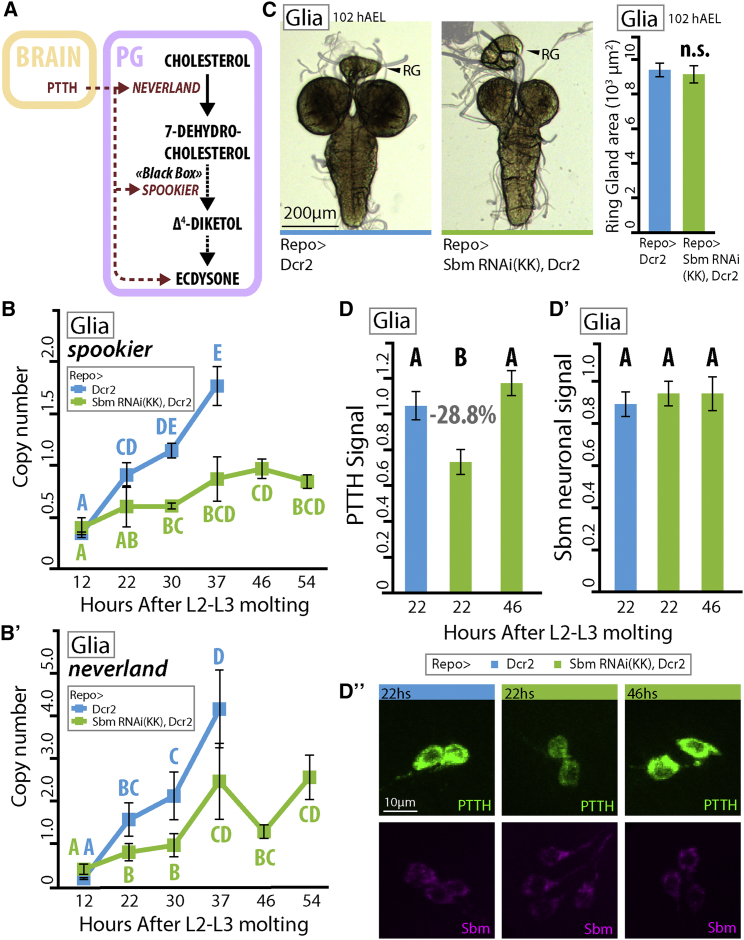
Effects on Developmental Timing Are Likely due to Disruption in PTTH and Ecdysone Levels (A) As development proceeds, prothoracicotropic hormone (PTTH) is produced in the brain of the larva and released in the prothoracic gland (PG), where it induces the expression of the genes of the molting hormone ecdysone synthesis pathway. (B and B′) Transcription levels of *spookier* (B) and *neverland* (B′) (expressed in copy number) at different time points in third-instar larvae (L3), synchronized at second- to third-instar molt, upon downregulation of *sbm* in glia (Repo-Gal4) (two-way ANOVA with a Duncan post-test; data were transformed to ln to fulfill the test criteria, p < 0.05, F = 3.12 and F = 3.93; n = 5). (C) Photograph depicting the difference in relative sized between the RG and brain in 102 hAEL larvae (equivalent to ∼37 hours after L2–L3 molt) and RG area measurements expressed in 10^3^ μm^2^ (t test t = 0.40; n.s., nonsignificant differences; n = 11). (D and D′) Somatic PTTH (D) and neuronal somatic Sbm levels (D′; as internal control) at different time points in synchronized third-instar larvae upon downregulation of *sbm* in glia (Repo-Gal4). In both cases, one-way ANOVA with a Duncan post-test was used. In (D), p < 0.05, F = 14.46, n = 40–46. In (D′), p = 0.71, F = 3.37, n = 40–46. (D″) Representative images of somatic PTTH (green) levels and Sbm (magenta) levels in the *sbm*-expressing neurons. In all graphs, data represent mean ± SEM, and different letters signify groups that are significantly different (p < 0.05) in a Duncan post-test.

In *D. melanogaster*, larval growth rate and the progression of larval stages are determined by levels of the molting hormone 20-ecdysone (20E). PTTH induces the expression of the genes that code for the enzymes responsible for synthesizing 20E in the prothoracic gland (McBrayer et al., 2007, Rewitz et al., 2009). A good proxy for assessing the levels of ecdysone production are the transcriptional levels of the genes of its synthesis pathway, such as *neverland* and *spookier* (Figure 6A) (McBrayer et al., 2007). We found that in control animals, the transcriptional levels of both genes rise until the moment of wandering, while the levels of these genes are lower in Repo > SbmRNAi larvae than controls at every time point. Most importantly, in Repo > SbmRNAi larvae, *spookier* transcriptional levels never reach the peak levels attained by controls at 37 hr after the L2–L3 molt (Figure 6B). Also, transcript levels of the ecdysone receptor (EcRA), an ecdysone-target gene (Varghese and Cohen, 2007), are reduced in 96 hAEL Repo > SbmRNAi larvae (∼24 hr after L2–L3 molt) relative to controls (Figure S6B). Taken together, these results strongly suggest that 20E production in *sbm* mutant larvae is reduced.

Systemic circulating Dilps activate the IIS pathway in the prothoracic gland (PG), a part of the ring gland (RG), increasing its size and increasing 20E levels (Colombani et al., 2005, Mirth et al., 2005). We measured the size of the RG in 102 hAEL larvae and did not find statistically significant differences between Repo > SbmRNAi larvae and controls (Figure 6C). This indicates that the increased Dilp expression and release observed (Figures 5D and 5E) do not significantly affect RG growth; hence, alterations in the transcription of the 20E synthesis genes should not be a consequence of Dilp signaling. Therefore, we hypothesized that alterations on PTTH levels may be responsible for the reduction in the transcription of 20E synthesis genes levels observed.

Because peak levels of PTTH are normally attained a few hours before peak transcriptional levels of ecdysone synthesis genes (McBrayer et al., 2007), we analyzed somatic PTTH levels in the brains of larvae at 22 hr after the L2–L3 molt. Immunoreactive signal levels were significantly reduced in RNAi-treated larvae compared to controls (Figures 6D and S6B). Interestingly, PTTH signal levels in Repo > SbmRNAi larvae at 46 hr after L2–L3 molting (a time point at which control animals have already pupated) were statistically indistinguishable from those of the controls 24 hr earlier (Figures 6D and S6B), indicating that lower levels of Sbm in glia do not abolish the rise in PTTH levels but rather produce a shift in the PTTH peak. This shift is likely the cause of ecdysone in the RG not reaching the levels needed to trigger pupation in the stereotypical timing, leading to the 24-hr prolongation of the larval third-instar observed in these animals and resulting in a phenotype strongly resembling the recently described PTTH-null mutants (Shimell et al., 2018).

## Discussion

**Figure 7:**
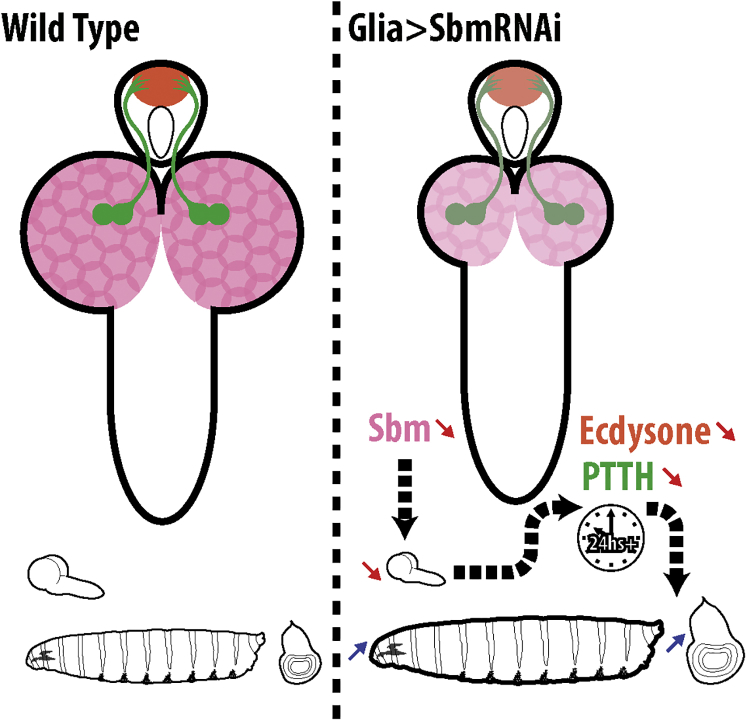
Model Sbm is expressed in glia of the larva (magenta). Downregulation of Sbm in glia provokes a delay in the development of the brain and a reduction in its final size (red arrow); in turn, this impacts the production of PTTH in PTTH neurosecretory cells (green) and leads to a reduction in expression of the genes responsible for the production of ecdysone in the PG (orange). This leads to a delay in the initiation of wandering and pupation, allowing the larvae an extra 24 hr in which tissues such as the represented wing imaginal disc continue growing (blue arrows), resulting in larvae and pupae of an increased size with relatively small brains.

In this work, we have shown that downregulation of *sbm* in glia provokes negative effects on developmental timing with consequent alterations in growth, and this effect is specific to the larval third-instar. This is associated with a delayed PTTH peak and reduced expression of 20E synthesis and target genes. These larvae have a significantly smaller brain than controls, with fewer proliferating cells and normal levels of dilp6 transcription and activation of the IIS pathway (Figure 7).

Developmental timing and growth are regulated through PTTH and ecdysone. During the larval third-instar in *D. melanogaster*, there is an important developmental checkpoint, the critical weight (CW), after which starvation will no longer delay pupation. CW is defined by a rise in the levels of ecdysone, a product of nutrient-dependent Dilp signaling and rising levels of PTTH (Mirth and Riddiford, 2007, Nijhout et al., 2014). Lack of PTTH provokes a shift in the CW and prolongs the growth periods before and after it (McBrayer et al., 2007, Shimell et al., 2018), resulting in bigger larvae. The phenotypes we describe in the present report are consistent with this.

### Nature of the Link between Glia and PTTH Neurons

Glial cells regulate the exit from quiescence of neuroblasts in third-instar larval brain (Lanet et al., 2013, Sousa-Nunes et al., 2011), allowing for an increase in proliferation and brain size. This regulation is mediated through two factors produced by glia: Dilp6 and Jellybelly (Jeb) (Cheng et al., 2011, Sousa-Nunes et al., 2011). Dilp6 is regulated at the transcriptional level according to the nutritional status of the larva (Okamoto and Nishimura, 2015, Sousa-Nunes et al., 2011). Jeb is constitutively produced in glia and allows for “brain growth sparing” in nutritionally adverse conditions, permitting the brain to grow at a higher rate than the rest of the body (Cheng et al., 2011). Both factors activate the phosphatidylinositol 3-kinase (PI3K) signaling pathway in quiescent neuroblasts; Dilp6 does it through the insulin receptor, while Jeb does it through the receptor Alk. We observed that brain growth and proliferation in the brain are highly impaired in glial-Sbm-deficient larvae, which could be due to reduced production of either of these two factors. Since our results indicate that *dilp6* transcription does not appear to be affected in this context, future work should focus on Jeb as a likely candidate in mediating the observed phenotypes. We also observed that PI3K pathway activation does not appear to be altered by Sbm glial downregulation. This raises the possibility that another unknown factor is involved, though it remains to be determined whether the release of Dilp6 from glial cells, the temporal dynamics of its transcription, or the temporal dynamics of PI3K pathway activation are affected. In any case, we have shown that glial downregulation of Sbm reduces brain size and that this may be in part due to reduced proliferation, which at least initially does not affect glia itself. This could mean that downstream to the glial signals, neuroblasts communicate with PTTH-producing neurons during their proliferation, allowing for the coordination of the progression of brain development with developmental timing, possibly ensuring that a minimal brain development is achieved before triggering the processes that indefectibly lead to pupation.

It is interesting to note that the recently described PTTH-null mutants (Shimell et al., 2018) show growth phenotypes very similar to the ones described in the present work, strengthening our model (Figure 7): a 24-hr delay to pupation in the larval third-instar without affecting the L2–L3 transition; an increase in larval, pupal, and adult size; a desynchronization of growth of different tissues; and altered transcription of ecdysone genes. More importantly, the PTTH-null mutation does not alter brain size, which adds to the idea that Sbm function in the glia is necessary to regulate brain growth, and this in turn regulates developmental timing (Figure 7).

It is important to add that the relationship between glia and PTTH could potentially be direct and involve close contact between Sbm^+^ glia and PTTH-producing neurons. Imaging and functional experiments should be carried out to explore this possibility.

### Cellular Function of Sbm

Whether the link between glia and PTTH neurons is direct or indirect, in both cases, the question that arises is how is the Sbm amino acid antiporter involved? As a LAT, Sbm could act by exchanging specific amino acids between the internal brain medium and glia, regulating in this way their relative concentrations and ensuring proper cellular functions. Transported amino acids could be themselves neurotransmitters or precursors for neurotransmitters. In this regard, Genderblind, another member of the SLC7 family expressed in some glia, has been described to regulate ambient glutamate levels, impacting neuronal physiology and social communication (Augustin et al., 2007, Featherstone, 2011, Grosjean et al., 2008, Piyankarage et al., 2008). Also, the SLC7 antiporter JhI-21 has a similar function in the NMJ (Ziegler et al., 2016). Alternatively, Sbm could exchange amino acids against Na^+^ (Fotiadis et al., 2013) and affect the glial resting membrane potential and, in doing so, alter the Ca^2+^ waves necessary for production and release of Dilp6 (Spéder and Brand, 2014). Lastly, Sbm could be transporting amino acids from the hemolymph and serving to sense the levels of one or more specific amino acids in the glia, as it has been described for Minidiscs in IPCs, which transports leucine (Manière et al., 2016). Further investigations describing Sbm amino acid transport capabilities will help to solve its action on PTTH neurons through glia.

## STAR★Methods

### Key Resources Table

**Table:** 

REAGENT or RESOURCE	SOURCE	IDENTIFIER
**Antibodies**		

Mouse monoclonal Anti-FLAG	Sigma	Cat#F3165; RRID:AB_259529
Rabbit polyclonal Anti-HA	Sigma	Cat#H6908; RRID:AB_260070
Rabbit polyclonal Anti-Phospho-Histone-3	Sigma	Cat# H0412; RRID:AB_477043
Mouse monoclonal Anti-HA	Eurogentec	Cat# AFC-101P; RRID:AB_291231
Rabbit polyclonal Anti-GFP	Thermo Fisher Scientific	Cat# A-11122; RRID:AB_221569
Mouse monoclonal Anti-GFP	Thermo Fisher Scientific	Cat# MA5-15256; RRID:AB_10979281
Mouse monoclonal Anti-ElaV	DSHB	Cat# 9F8A9; RRID:AB_2314364
Mouse monoclonal Anti-Repo	DSHB	Cat# 8D12; RRID:AB_528448
Guinea Pig polyclonal Anti-PTTH	Yamanaka et al., 2013	N/A
Rat polyclonal Anti-Dilp2	Géminard et al., 2009	N/A
Rabbit polyclonal Anti-Sbm	This paper	N/A
Goat polyclonal anti-mouse IgG-HRP	Santa Cruz Biotechnology	Cat# sc-2005; RRID:AB_631736
Goat anti-Rabbit IgG (H+L) Cross-Adsorbed Secondary Antibody, Alexa Fluor 488	Thermo Fisher Scientific	Cat# A11008; RRID:AB_141365
Goat anti-Rabbit IgG (H+L) Cross-Adsorbed Secondary Antibody, Alexa Fluor 594	Thermo Fisher Scientific	Cat# A11012; RRID:AB_2534079
Goat anti-Rat IgG (H+L) Cross-Adsorbed Secondary Antibody, Alexa Fluor 488	Thermo Fisher Scientific	Cat# A-11006; RRID:AB_2534074
Goat anti-Mouse IgG (H+L) Cross-Adsorbed Secondary Antibody, Alexa Fluor 488	Thermo Fisher Scientific	Cat# R37120; RRID:AB_2556548
Goat anti-Guinea Pig IgG (H+L) Highly Cross-Adsorbed Secondary Antibody, Alexa Fluor 488	Thermo Fisher Scientific	Cat# A-11073; RRID:AB_2534117
Goat anti-Mouse IgG (H+L) Cross-Adsorbed Secondary Antibody, Alexa Fluor 594	Thermo Fisher Scientific	Cat# A-11005; RRID:AB_2534073
Goat anti-Mouse IgG1 Cross-Adsorbed Secondary Antibody, Alexa Fluor 633	Thermo Fisher Scientific	Cat# A-21126; RRID:AB_2535768

**Experimental Models: Cell Lines**		

*Drosophila* S2 Cells	Thermo Fisher Scientific	Cat# R69007

**Experimental Models: Organisms/Strains**		

*D. melanogaster* UAS-Sbm RNAi	Vienna Drosophila Resource Center	VDRC #108867
*D. melanogaster* UAS-Sbm RNAi(GD)	Vienna Drosophila Resource Center	VDRC #45180
*D. melanogaster* UAS-Dcr2	Vienna Drosophila Resource Center	VDRC #60009
*D. melanogaster* AttP2	Kyoto Drosophila Genomics and Genetic Resources	DGGR #36303
*D. melanogaster* Myo1D-Gal4	Kyoto Drosophila Genomics and Genetic Resources	DGGR #113094
*D. melanogaster* NP2276-Gal4	Kyoto Drosophila Genomics and Genetic Resources	DGGR #112853
*D. melanogaster* NP6520-Gal4	Kyoto Drosophila Genomics and Genetic Resources	DGGR #105240
*D. melanogaster* NP3233-Gal4	Kyoto Drosophila Genomics and Genetic Resources	DGGR #113173
*D. melanogaster* Dilp6-Gal4	Kyoto Drosophila Genomics and Genetic Resources	DGGR #103877
*D. melanogaster* Appl-Gal4	Bloomington Drosophila Stock Center	BDSC #32040
*D. melanogaster* Syb-Gal4	Bloomington Drosophila Stock Center	BDSC #51635
*D. melanogaster* 18E11-Gal4	Bloomington Drosophila Stock Center	BDSC #45446
*D. melanogaster* Repo-Gal4	Bloomington Drosophila Stock Center	BDSC #7415
*D. melanogaster* Ppl-Gal4	Bloomington Drosophila Stock Center	BDSC #58768
*D. melanogaster* Nrv2-Gal4, UAS-GFP	Bloomington Drosophila Stock Center	BDSC #6794
*D. melanogaster* Tub-Gal4	Bloomington Drosophila Stock Center	BDSC #5138
*D. melanogaster* UAS-GFP	Bloomington Drosophila Stock Center	BDSC # 5130
*D. melanogaster* Act5C(FRT.CD2)Gal4	Bloomington Drosophila Stock Center	BDSC #4780
*D. melanogaster* UAS-FLP	Bloomington Drosophila Stock Center	BDSC #4539 and #8209
*D. melanogaster* P0206-Gal4	Gift from Dr. Pablo Wappner (Colombani et al., 2005	N/A
*D. melanogaster* Alrm-Gal4	Gift from Dr. Marc Freeman (Doherty et al., 2009)	N/A
*D. melanogaster* MZ0709-Gal4	Gift from Dr. Marc Freeman (Doherty et al., 2009)	N/A
*D. melanogaster* Repo-Gal80	Gift from Dr. Yuh Nung Jan (Awasaki et al., 2008	N/A
*D. melanogaster* Sbm-Gal4	This paper	N/A
*D. melanogaster* UAS-SbmPA	This paper	N/A

**Oligonucleotides**		

Please refer to Table S1.	N/A	N/A

**Recombinant DNA**		

BACR22I24	BAC PAC Resources at the Children’s Hospital Oakland Research Institute	Genebank ID AC022346

### Contact for Reagent and Resource Sharing

Further information and requests for resources and reagents should be directed to and will be fulfilled by the lead contact, Yael Grosjean (yael.grosjean@u-bourgogne.fr)

### Experimental Model and Subject Details

#### Fly stocks and rearing conditions

Flies were reared in food containing 6.5% corn flour, 6.5% yeast extract, 1% Agar-Agar and 3% of a 0.1% Tegosept (Apex) antifungal solution in ethanol. Rearing temperature was 25°C for ubiquitous expression experiments. In all other experiments crosses, ovipositions and embryo development were carried at 25°C while development from the 1^st^-instar stage onward was conducted at 29°C in constant darkness. In all experiments with larvae, animals used were of unknown gender. In experiments with adult flies, only male flies were used to avoid variability due to gender dimorphism affecting size. A complete list of fly stocks is given in the Key Resources Table.

#### S2 cell culture

Cells were cultured in Schneider’s medium (Invitrogen) containing penicillin (50 u/mL), streptomycin (50 μg/mL) and 10% fetal bovine serum (FBS), at 28°C.

### Method Details

#### sbm transcript recognition, cloning and tagging

Total RNA was extracted using Isol-RNA Lysis Reagent (5 Prime) and cDNA synthesized using iScript Select cDNA Synthesis Kit (BioRad) with OligodT and a Random Primers (for cloning the different mRNAs) or specific primer (for identification of transcripts). PCR for transcript identification and cloning of different isoforms was done using specific primers listed in Table S1. Amplification products were purified using NucleoSpin Extract II (Macherey Nagel) and cloned into the TOPO entry vector (Invitrogen).

Protein tagged versions were generated using the primers listed in Table S1, cloned into TOPO vector (Invitrogen) for FLAG versions or pCR8 vector for HA version, and subcloned into the S2 cell expression vector pMT-DEST48 (Invitrogen) using the Gateway cloning system (Invitrogen).

#### Sbm GAL4 and UAS-Sbm cloning

To generate the Sbm-Gal4 transgenic line we analyzed the genome region of the *sbm* gene with Evoprinter (https://evoprinter.ninds.nih.gov/), comparing to the *D. sechellia*, *D. yakuba*, *D. erecta* and *D. Pseudoobscura* sequences and found that there were sections of the introns of the gene with a degree of conservation that suggested that they could contain regulatory sequences. We cloned into the TOPO vector (Invitrogen) an approximately 3.4 kb long fragment of the *locus* including the introns of the RC and RD transcripts, from the template BACR22I24 (Genebank ID AC022346, obtained from the BAC PAC Resources at the Children’s Hospital Oakland Research Institute) with the primers listed in Table S1. Using the Gateway system (Invitrogen) we subcloned this fragment into the pBPGAL4.2Uw-2 (a gift from Gerald Rubin, Addgene plasmid #26227, (Pfeiffer et al., 2010) and sent to Genetic Services, Inc. (MA, USA) for injection into their *y, w; attP2* line (location 68A4, chromosome 3L.)

To generate the UAS-Sbm transgenic line, we used the Gateway system (Invitrogen) to subclone the CDS from the TOPO vector to the pUASg:attB vector (Bischof et al., 2013) and sent to Genetic Services, Inc. (MA, USA) for injection into their *y, w; attP2* line (location 68A4, chromosome 3L.)

#### Sbm antibody

Polyclonal anti-Sbm antibody was produced by immunization of rabbits with the synthetic peptide EAPETDSSGTGRMRKPLE corresponding to amino acids 42-59 of Sbm-PA and PD, and amino acids 51-68 of Sbm-PB and PC (Eurogentec).

#### S2 cell immunocytology and western blots

Cells were seeded at 0.5 million cells/mL 30 h before transfection in multiwell plates with the same medium, then washed in Schneider’s medium without FBS and transfected with FuGENE HD Transfection Reagent (Promega) in a ratio of 4.5:1 to sterile plasmid DNA (0.25 μg/μL). Cells were incubated for 17 h in the medium without FBS followed by 8 h in medium with 10% FBS and induced for 21 h with 0.5 mM copper sulfate.

For immunocytology cells were seeded on wells containing poly-L-lysine treated coverslips (0.01%, Sigma) before induction. After induction, they were washed three times with PBS and two times with PBS-Glycine 0.1M. Cells were permeabilized with Cytoperm (BD-Bioscience) and washed with PBS-Saponin 0.05%. Cells were incubated for 1 h with primary antibodies, then washed and further incubated with secondary antibodies for 30’ and with Hoechst (1 μg/mL) for 5 minutes to mark nuclei. Cells were mounted in Fluorescent Mounting Medium (Dako). Images were obtained with a Leica TCS SP2 confocal microscope.

For western blots, transfected cells were scrapped, lysed 30 min at 4°C with 1% Triton X-100 buffer (150 mM NaCl; 50 mM Tris-HCl pH8; Triton X-100 1%; protease inhibitor Roche, 1 mM PMSF) and total proteins were quantified by Lowry method (“DC Protein Assay” kit from Biorad). Samples were then treated in Laemmli Buffer containing or not reducing agent (700 mM beta-mercaptoethanol; 200 mM DTT). Proteins were separated on polyacrylamide gel and then transferred on PVDF membranes. The membrane was probed with mouse anti HA antibody and then with the HRP conjugated secondary antibody, before being revealed with ECL reagent (Amersham biosciences).

#### Dissections and Immunostainings

Larval tissues were dissected in PBS and fixed in 4% paraformaldehyde (PFA) for 20 min at room temperature (RT), rinsed 3x15 min in PBS with 0.3% Triton X-100 (PT), and blocked with 7% normal goat serum (NGS; Sigma #G9023) for 45 min, followed by incubation with primary antibody at 4°C (overnight, or 48 h to 72 h). Samples were washed 4x15 min in PT, incubated with secondary antidobies for 2-3 h at RT, washed 4x15min in PT and mounted in Vectashield mounting medium. A list of the primary and secondary antibodies is listed in Table S2. For Phalloidin (Thermo Fisher Scientific) and Hoescht 33342 (Thermo Fisher Scientific) stainings a working solution was added together with, or 15 min immediately after secondary antibodies, respectively.

#### Fluorescence quantification

Images were taken on either a Leica TCS SP2, a Leica TCS SP8 or an LSM 880 Axio Imager 2 Zeiss confocal microscope. All quantitative analyses were performed employing FIJI Software. Within each experiment, all pictures were taken employing the same confocal settings and in the case of the Leica TCS SP8 Confocal microscope, using its Hybrid mode. Sbm signal in neurons, glia and gut, and somatic PTTH and somatic dilp2 levels were quantified from confocal images. In all cases, we analyzed the mean gray value, a measurement that is independent of the area.

For quantification of Sbm signal in glial cells, confocal images of brain hemispheres stained against Sbm and REPO were taken with a 20X objective and an optical zoom of 1.5X using a 1 μm step size. First, a Z projection of 6 stacks showing Sbm^+^ glial signal was made. Then, a region of interest (ROI) was selected, adjusting the threshold image in order to mark most of the Sbm^+^ glial signal. Mean Sbm fluorescence intensity and area was measured within the ROI created. A rectangle of the same or a higher area was located outside of glial membrane and used to subtract background signal. For the analysis, we considered the mean gray value (with the subtracted background signal) a measurement that is independent of the area. The same protocol was applied to measure Sbm levels in neuronal somas.

The mean gray value of PTTH in somas was quantified from confocal Z stacks of brains stained against Sbm and PTTH. Photos were obtained with a 20X objective with an optical zoom of 3X using a 0.69 μm step size. The signal of Sbm in neuronal somas was also quantified to compare its variation to that of PTTH. To compare the different genotypes and developmental times, PTTH values were normalized to the average PTTH signal of each independent experiment.

For dilp2 immunoreactive analysis within the IPCs, confocal images of brains stained against Dilp2 and FOXO (to better localize IPCs somas) were taken with a 40X objective and an optical zoom of 3 using a 0.69 μm step size. The mean gray value signal from Z stacks was measured as before, and data was normalized to the average dilp2 signal of each independent experiment to compare between genotypes.

Nuclei of glial cells (anti-Repo) and of dividing cells (anti-PH3) were counted using the Quantitation Module of Volocity® 6.3 software (Quorum Technologies Inc., Ontario), on LASX average Z projection images of one brain lobe, pre-treated with median filter.

In all cases of fluorescent signal quantification, the sample size is the number of brains measured, each brain is considered a replicate.

#### Developmental timing, growth assessment and feeding assays

Crosses were performed in oviposition chambers and eggs collected onto an agar plate supplemented with yeast paste for 4 hours. Recently hatched 1st-instar larvae were transferred to rearing vials (60 L1 larvae per vial). The number of pupa was counted and pupal volume was calculated as previously described (Galagovsky et al., 2014). Each mutant condition and its specific control were assayed strictly at the same time to allow their precise comparisons. Larval and male adult weight was measured with a Sartorius R160P balance. In the case of larvae, each replicate represents the average of 3 groups of 8 larvae. Virgin males were kept in groups of 15, left for 2 days at 25°C and frozen at −80°C until weighted. Wing size measurements were performed as in (Galagovsky et al., 2014). For feeding assays, larvae of the appropriate age were transferred to regular food tubes supplemented with 0.5% Brilliant Blue FCF (Sigma). After 2 h, larvae were recovered, thoroughly rinsed, dried and frozen at −80°C in groups of 8. The amount of food ingested was determined by absorbance measurement of FCF dye at 625 nm. Three groups of 8 larvae were averaged for each point.

#### Organ size measurement

Larvae were dissected and fixed in 0.4% PFA. Organs were dissected and mounted in 80% Glycerol in PBS, pictures taken with a Leica DM5000B microscope and measured using the FIJI software. Fat bodies were previously stained with Sudan Black (Sigma) following the protocol in (Tennessen et al., 2014). Brain lobe volume was estimated by approximating it to a sphere (V = 4/3π(1/2W)^3^) where W is the average of the width of both brain lobes of a same brain.

#### Real Time PCRs

Total RNA for *dilp2*, *dilp5*, *dilp6* and *thor* analysis was prepared from 20 120 hAEL third-instar larval brains per sample. Larvae were dissected in PBS and brains immediately placed in Isol-RNA Lysis reagent (5-Prime) on ice and then lysed using a TissueLyser (QIAGEN) and frozen at −80°C until use. For *spookier* and *neverland* samples were prepared from 8 whole third instar larvae per sample, carefully synchronized at the second to third instar transition. For *sbm* 10 whole third instar larvae per sample were used. 4 to 7 independent samples were prepared for each point. In all cases *rpl29* was used to normalize. To determine copy number a standard curve was performed from a mix of equal amounts of all the samples in each experiment.

RNA was treated with DNAase (Thermo Fisher Scientific) and cDNA was synthesized using iScript cDNA synthesis kit (BioRad). Real Time PCRs were carried out using the IQ SYBR Green Supermix (BioRad) in a MyiQ single-color Real-Time PCR detection system (BioRad). Primers used are detailed in Table S1.

### Quantification and Statistical Analysis

Statistical analyses were performed with the InfoStat package version 2009 (Grupo InfoStat, FCA, Universidad Nacional de Cordoba, Argentina). To determine if data met the criteria for ANOVA analysis normality was tested using Shapiro-Wilks test and the homogeneity of variance was assessed with Levene’s test. p < 0.05 was considered statistically significant. In the cases were data did not meet these criteria, transformations were. If transformations were not sufficient to meet the criteria, non-parametric analysis were performed. For the analysis of pupation curves, each independent replicate, i.e., each vial with 60 initial larvae, was adjusted to a Gompertz non-linear regression model, and the median time to pupation was estimated. The statistical tests, number of replicates and special requirements for each experiment, as well as type of data transformation performed if necessary are detailed in the legends of the corresponding figures.

## Supplemental Information

Document S1. Figures S1–S6 and Table S1

Document S2. Article plus Supplemental Information
