# Supplementary material for: Sobremesa L-type Amino Acid Transporter Expressed in Glia Is Essential for Proper Timing of Development and Brain Growth
Source: Cell Rep. 2018 Sep 18;24(12):3156–3166.e4. doi: 10.1016/j.celrep.2018.08.067 (PMC6167638; doi:10.1016/j.celrep.2018.08.067)
Supplement: Document S1. Figures S1–S6 and Table S1 [file mmc1.pdf]

Cell Reports, Volume *24*

Supplemental Information

Sobremesa L-type Amino Acid Transporter Expressed in Glia Is Essential for Proper Timing of Development and Brain Growth

Diego Galagovsky, Ana Depetris-Chauvin, Gérard Manière, Flore Geillon, Martine Berthelot-Grosjean, Elodie Noirot, Georges Alves, and Yael Grosjean

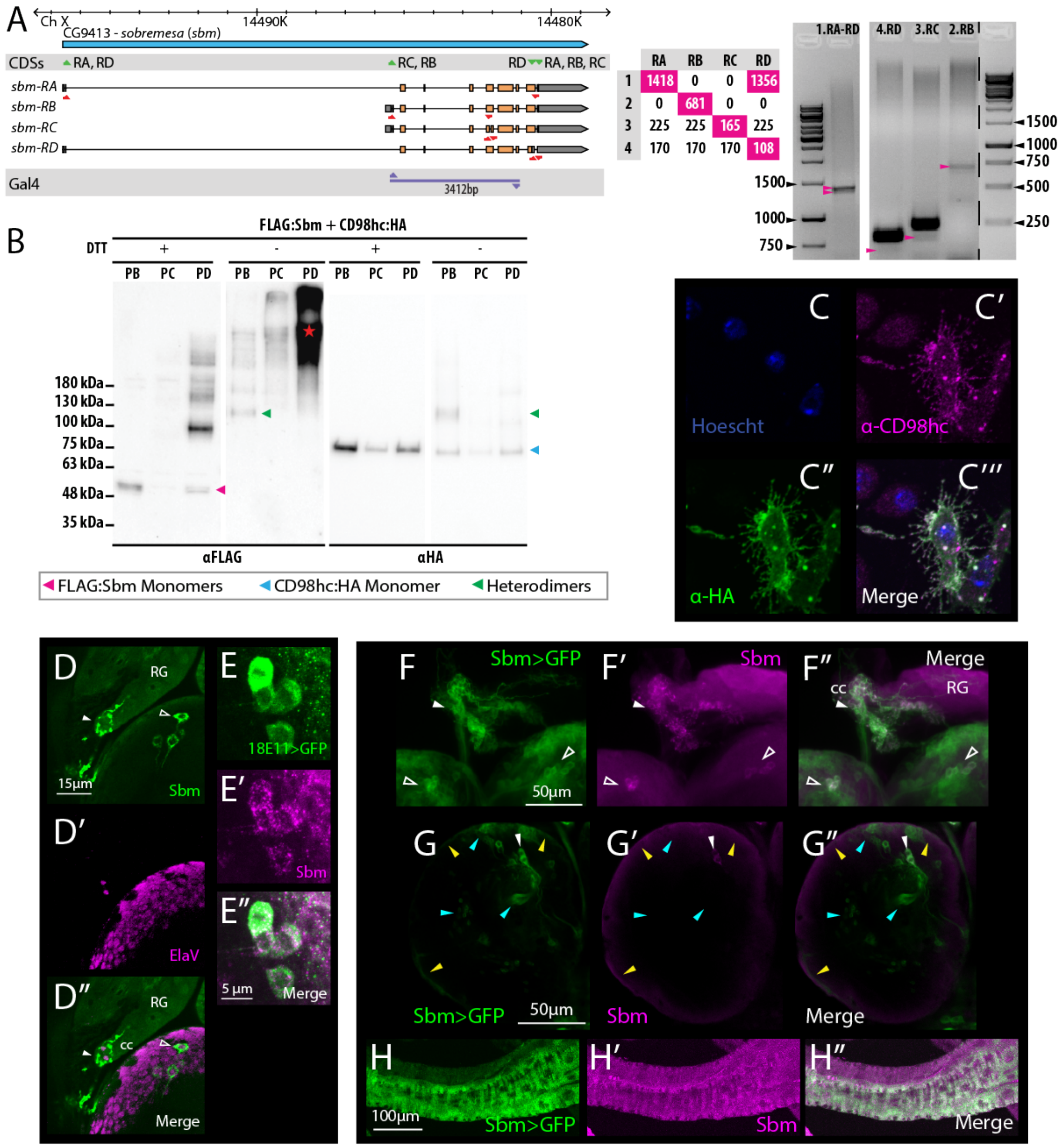

FIGURE S1. ***sobremesa*** **(CG9413 –** ***sbm*****) gene structure, products, expression pattern and localization. Related to Figure 1.** (A) Gene location and structure as reported in Flybase. We confirmed the existence of the four putative mRNAs. The two forward and two reverse primers used to amplify and clone the CDSs are shown in green on their target location relative to the mRNAs and the genomic *locus*. After cloning, colonies were screened using the pairs of primers

shown in red and selected according to the size of the amplification product obtained, as detailed on the table (magenta numbers indicate the unique bands used as diagnostic). Two gels showing diagnostic bands (magenta arrow heads) amplified by PCR from total cDNA are shown on the far right. In lilac is shown the region of the *locus* cloned and the primers used to generate the Sbm-Gal4 driver line. (B) Western Blot of extracts from S2 cells expressing FLAG:Sbm-PB, PC or PD together with CD98hc:HA in the presence (+) and absence (-) of reducing agent (DTT), revealed with anti-FLAG and anti-HA antibodies. The expected position of the heterodimer and monomer bands are pointed based on the molecular weight marker. The red star indicates signal saturation. For FLAG tagged Sbm-PB expected heterodimer bands are revealed in the absence of DTT and disappear or are fainter in the presence of DTT, while monomer bands behave in the opposite way. (C) The expression of CD98hc in S2 cells provokes membrane extensions. Pictures show in blue, Hoescht; magenta, CD98hc and green, HA. (D) Sbm antibody reveals expression in a group of cells in the brain (somas-hollow arrowhead, projections-filled arrowheads) projecting to the corpora cardiaca (cc) portion of the ring gland (RG). Staining against ElaV confirms that these cells are neurons. (E) The transgenic line 18E11-Gal4 drives expression in the Sbm^+ neurons as revealed by expression of GFP (18E11>GFP in green) in the Sbm immunoreactive neurons (anti-Sbm in magenta.) (F-H) The Sbm-Gal4 transgenic line drives expression in the Sbm immunoreactive tissues and cells. (F) Sbm-Gal4 driven expression of GFP (green) can be observed in the somas (hollow arrowheads) and in the projections (filled arrowheads) of the neurons that express Sbm (magenta) and innervate the corpora cardiaca (cc) of the ring gland (RG). (G) Sbm-Gal4 drives expression of GFP (green) in the Sbm^+ surface glia (magenta); yellow arrowheads point to spots of similar signal localization; a white arrowhead marks the Sbm^+ neurons. Sbm-Gal4 also drives expression in many cells that do not show Sbm immunoreactive signal (examples are marked by cyan arrowhead.) (H) Sbm-Gal4 drives expression of GFP (green) in the region of the hindgut of the larva that shows immunoreactive Sbm signal (magenta.)

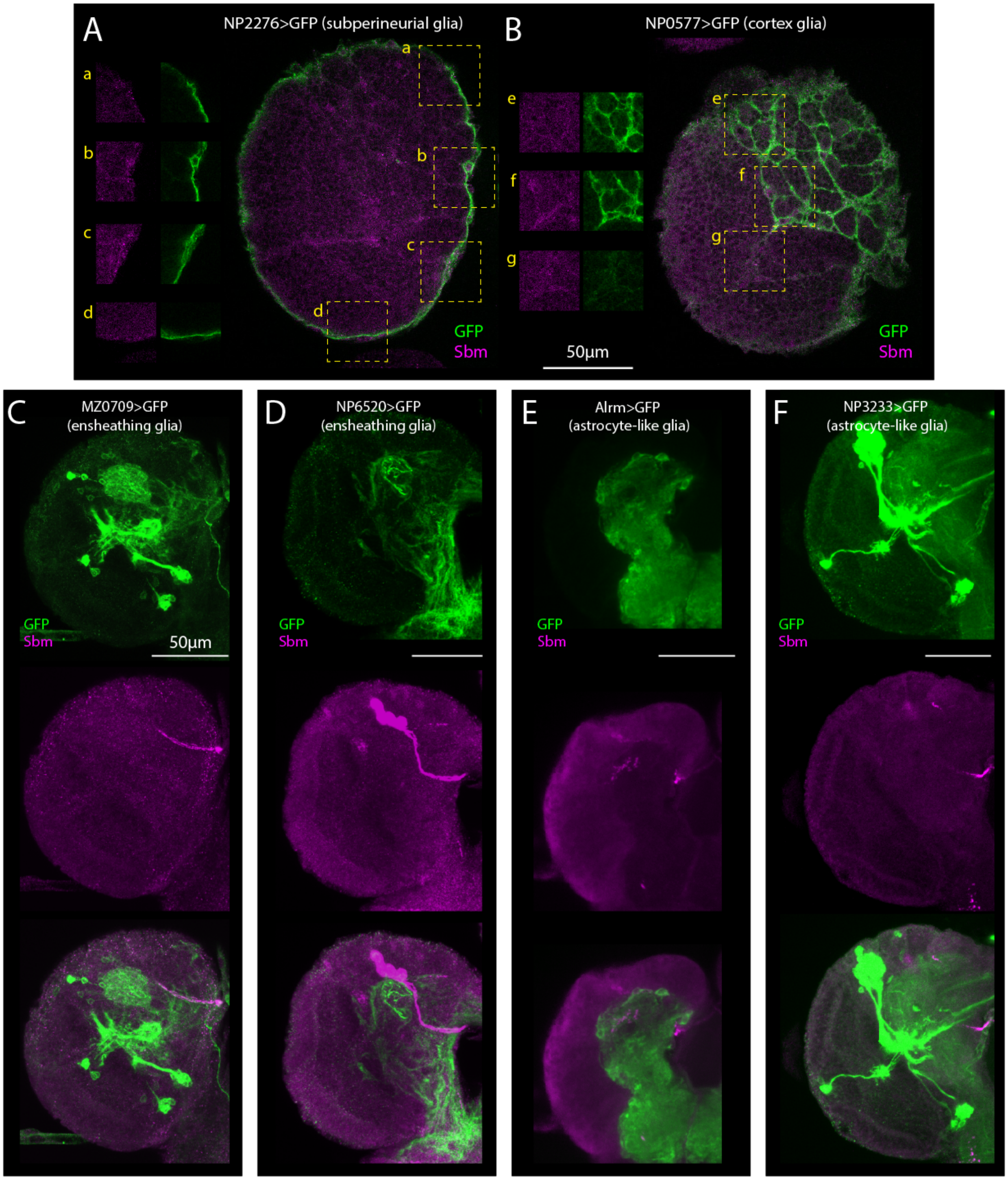

FIGURE S2. Sbm expression is observed in subperineurial and cortex glial subtypes, but not in other subtypes of glial cells. Related to Figure 1. (A-B) Subperineurial glia marked through the expression of GFP (green) driven by the NP2276-Gal4 line (A) and cortex glia marked through the expression of GFP (green) driven by the NP0577-Gal4 line (B), show expression of Sbm

(magenta). The merged signals are shown in the full frames. Squares of broken yellow line mark the position of the corresponding insets (a-g) which show details of the separate channels in A and B. (C-F) Expression of Sbm (magenta) is not observed overlapping other glial subtypes marked by the expression of GFP (green) driven by lines MZ0709-Gal4 (C) and NP6520-Gal4 (D) (ensheathing glia), and Alrm-Gal4 (E) and NP3233-Gal4 (F) (astrocyte-like glia). In all pictures the scale bar represents 50 µm. Pictures in A-B are single confocal planes. Pictures in C-F are Z-Stacks of single confocal planes.

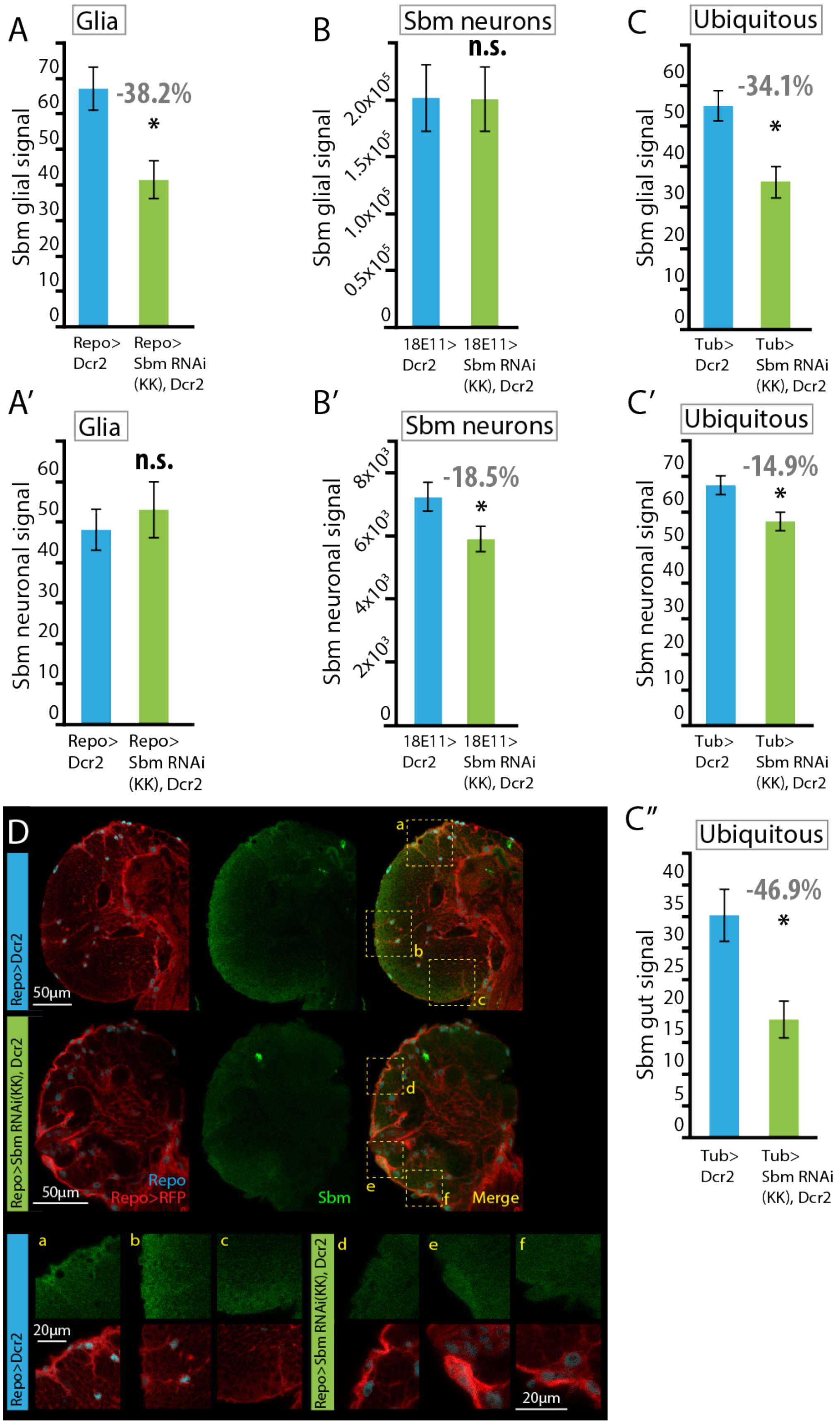
A
Glia
Sbm glial signal
70
60
50
40
30
20
10
0
-38.2%
*
Repo> Dcr2
Repo> Sbm RNAi (KK), Dcr2
B
Sbm neurons
n.s.
Sbm glial signal
2.0x10^5
1.5x10^5
1.0x10^5
0.5x10^5
0
18E11> Dcr2
18E11> Sbm RNAi (KK), Dcr2
C
Ubiquitous
Sbm glial signal
60
50
40
30
20
10
0
-34.1%
*
Tub> Dcr2
Tub> Sbm RNAi (KK), Dcr2
A′
Glia
n.s.
Sbm neuronal signal
60
50
40
30
20
10
0
Repo> Dcr2
Repo> Sbm RNAi (KK), Dcr2
B′
Sbm neurons
-18.5%
*
Sbm neuronal signal
8x10^3
6x10^3
4x10^3
2x10^3
0
18E11> Dcr2
18E11> Sbm RNAi (KK), Dcr2
C′
Ubiquitous
-14.9%
*
Sbm neuronal signal
70
60
50
40
30
20
10
0
Tub> Dcr2
Tub> Sbm RNAi (KK), Dcr2
D
Repo>Dcr2
Repo>Sbm RNAi(KK), Dcr2
50µm
a
b
c
d
e
f
Repo
Repo>RFP
Sbm
Merge
20µm
C″
Ubiquitous
-46.9%
*
Sbm gut signal
40
35
30
25
20
15
10
5
0
Tub> Dcr2
Tub> Sbm RNAi (KK), Dcr2

FIGURE S3. Expression of an RNAi against *sbm* reduces the levels of Sbm protein. Related to Figure 2. (A-A’) Expression of an RNAi against *sbm* in glia (Repo-Gal4) reduces the levels of Sbm protein in the glia (A) but not in the Sbm immunoreactive neurons (A’).(t-Test; t=-3.22 for A and t=0.62 for A’;*, $p<0.05$; error bars, S.E.M; n =22-24). (B-B’) Expression of an RNAi against *sbm* in the Sbm immunoreactive neurons (18E11-Gal4) does not reduce the levels of Sbm protein in glia (B) but significantly reduces the levels of Sbm protein in the Sbm immunoreactive neurons (B’) (t-Test; t=0.72 for B and t=2.94 for B’;*, $p<0.05$; error bars, S.E.M; n =32-30). (C-C’) Expression of an RNAi against *sbm* ubiquitously (Tub-Gal4) reduces the levels of Sbm protein in glia (C), in the Sbm immunoreactive neurons (C’) and in the gut (C’’) (t-Test; t=3.54 for C, t=2.72 for C’ and t=3.5 for C’’; *, $p<0.05$; error bars, S.E.M; n =20-22). (D) Pictures illustrating Sbm (green) levels in the glia marked by RFP (Repo>RFP) to show its membrane structure and with Repo antibody (cyan) to show the cell nuclei. The insets a-f show a detailed view of the Repo and Sbm signals in the corresponding regions marked in the Merge signal panels by yellow broken line squares. The RNAi expressing brains show diminished signal in the glial cells but the corresponding glial subtype membranes (marked by repo>RFP) is still visible.

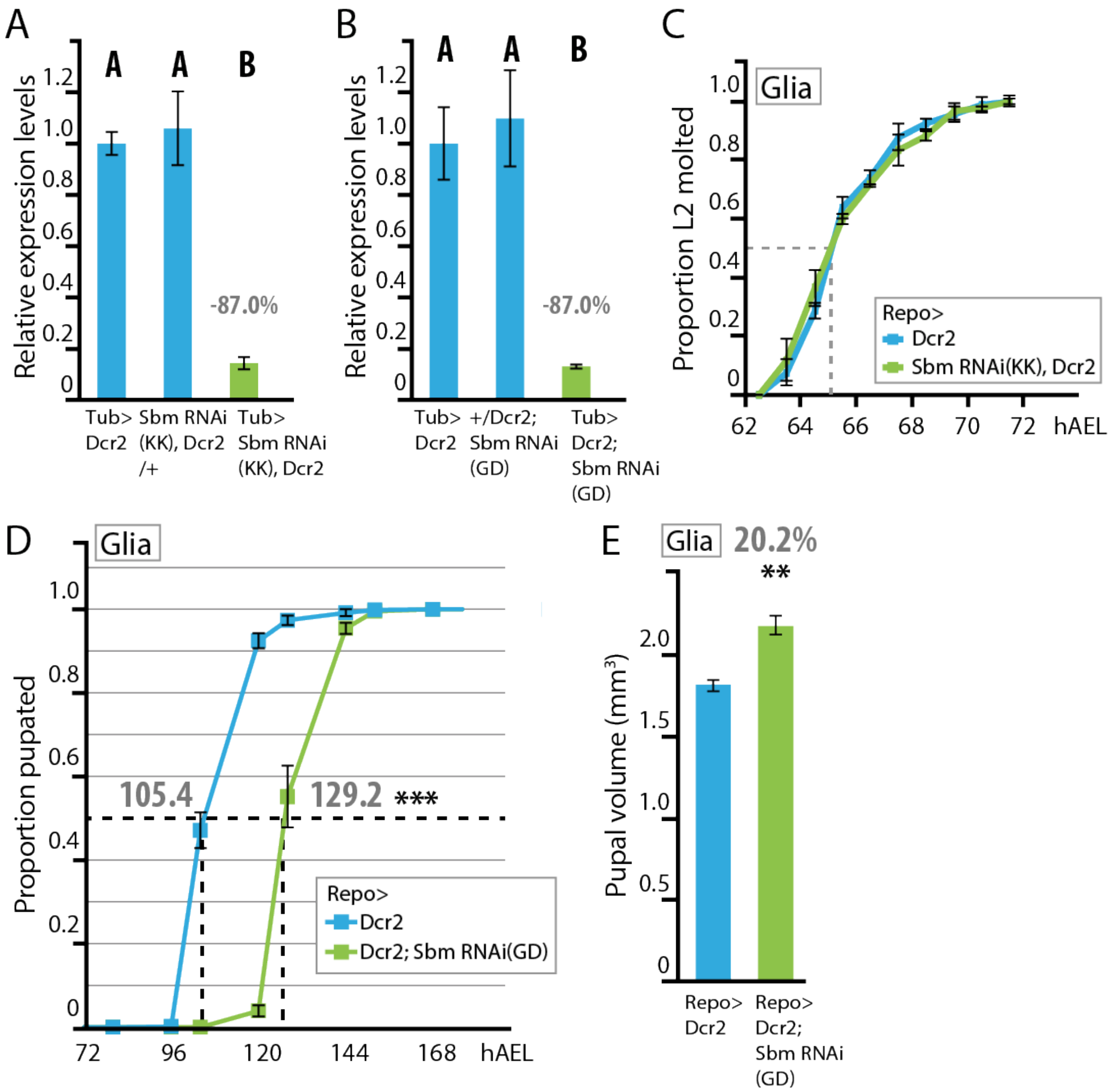

FIGURE S4. Expression of RNAi against *sbm* reduces the levels of *sbm* transcript. Related to Figure 2. (A-B) Quantitative RT-PCR showing that ubiquitous expression (Tub-Gal4) of (A) SbmRNAi (KK) or (B) SbmRNAi (GD), both combined with Dcr2, reduce the levels of *sbm* transcript relative to those of the controls (either Tub>Dcr2 or the heterozygous RNAis) (A, One-Way ANOVA, $p<0.0001$, $F=65.16$; bars with different letters signify that they are significantly different with $p<0.05$ in a Tukey post-test; error bars, S.E.M.; n=4-7; B, One-Way ANOVA, $p<0.0001$, $F=23.37$ with Tukey post-test; different letters, $p<0.05$; error bars, S.E.M.; n=4-5). **L2-L3 molt timing is not altered in the larvae expressing Sbm RNAi in glia.** (C)

Proportion through time of Repo>Dcr2 and Repo>SbmRNAi, Dcr2 second-instar larvae molting to the third instar shows no difference, demonstrating that the developmental delay caused by Sbm down-regulation is specific to the third-instar larval stage. **Effects of *sbm* downregulation in glia on developmental timing and growth, through the use of a second RNAi line.** (D-E) Proportion of larvae pupated through time (hAEL, hours after egg laying.) The expression of a second RNAi (SbmRNAi(GD), Dcr2) driven in glia by Repo-Gal4 leads to a 24 h delay to pupation (D), and an increase in pupal volume (E). (In D, t-Test, $t=15.62$; ***, $p<0.0001$; $n=11-12$, and in E, t-Test, $t=5.93$; **, $p<0.0001$.; $n=7$. Error bars, S.E.M.)

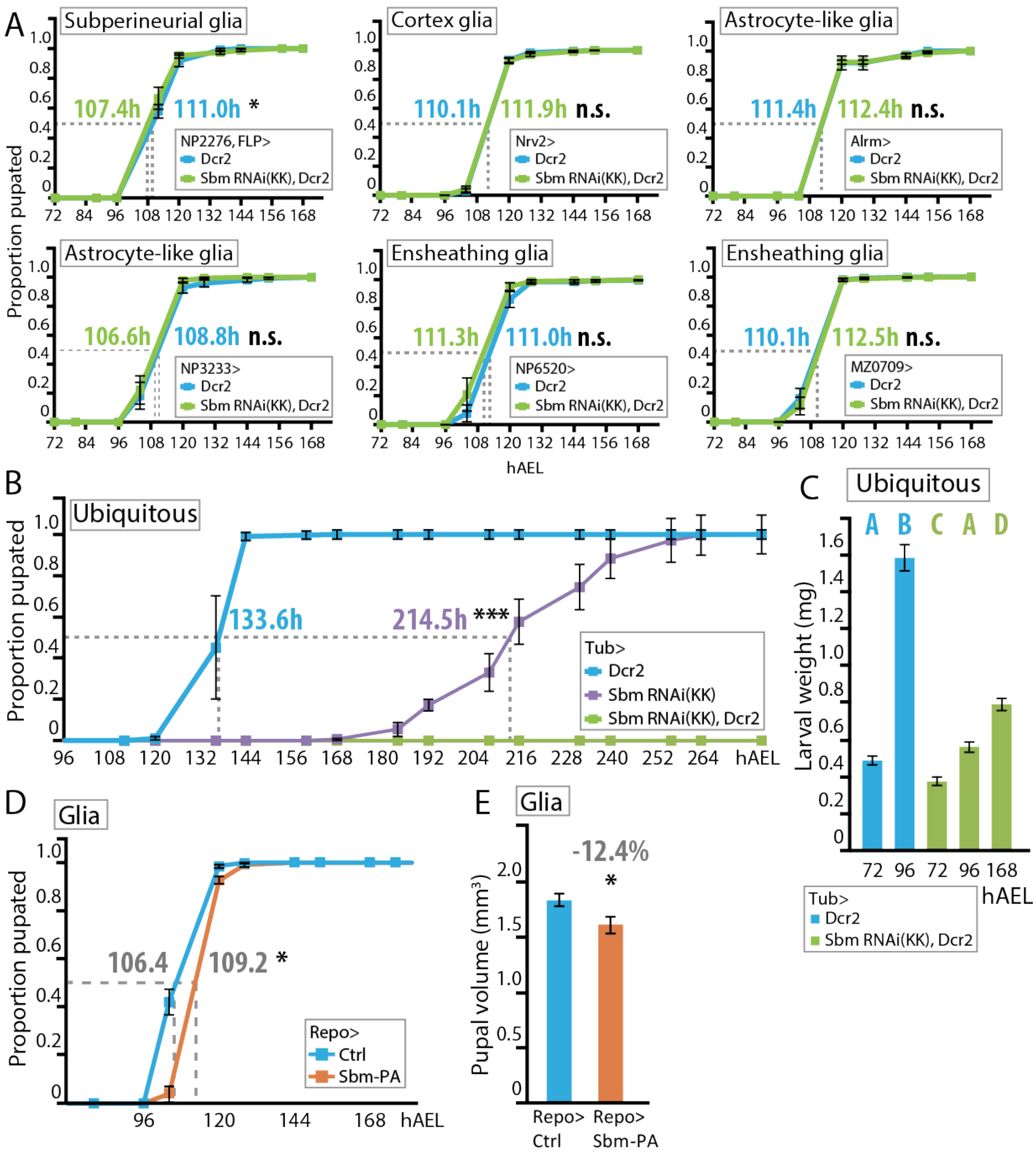

FIGURE S5. Downregulation of *sbm* in specific glial subtypes. Related to Figure 2. (A) Proportion of larvae pupated through time. In all cases the Gal4 driver is indicated on the top left corner of the graph and the glial subtype of expression on the bottom right. Blue lines and dots represent Dcr2 overexpression and green, Sbm RNAi combined with Dcr2. Subperineurial glial drivers were combined with UAS-FLP using the Act5C(FRT.CD2)GAL4 strain, to increase

expression by driving it with the strong *actin* gene promoter in the specific glial expression pattern (t-Test, from left to right and top to bottom t=2.52, t=1.44, t=0.38, t=0.68, t=0.17, t=1.29; n.s., non-significant differences; *, p=0.036; error bars, S.E.M.; n=5, n=10, n=3, n=3, n=4, n=10). **Effects of *sbm* ubiquitous downregulation on developmental timing and growth.** (B-C) Ubiquitous overexpression with the Tub-Gal4 driver of Dcr2 (blue in all graphs), SbmRNAi (purple) and both combined (green in all graphs) (B) Proportion of larvae pupated through time. (t-Test, t=9.18; *, p<0.0001; error bars, S.E.M.; n=5) (C) Larval weight at different developmental time points (Two-Way ANOVA, data was transformed to LN to fulfill the test criteria, p<0.0001, F=148.36; bars with different letters signify that they are significantly different with p<0.05 in a Duncan Post-Test; error bars, S.E.M.; n=5-7 per age and genotype). **Effects of *sbm* overexpression on developmental timing and growth.** (D-E) The expression of Sbm-PA driven in glia by Repo-Gal4 leads to a slight delay to pupation (D), and a significant decrease in pupal volume (E). (In D, t-Test, t=1.8; *, p<0.046; n=7, and in E, t-Test, t=2.32; **, p<0.05; error bars, S.E.M.; n=5. Error bars, S.E.M.) In all developmental curves, the mean median value in hours is indicated for each curve. hAEL, hours after egg laying.

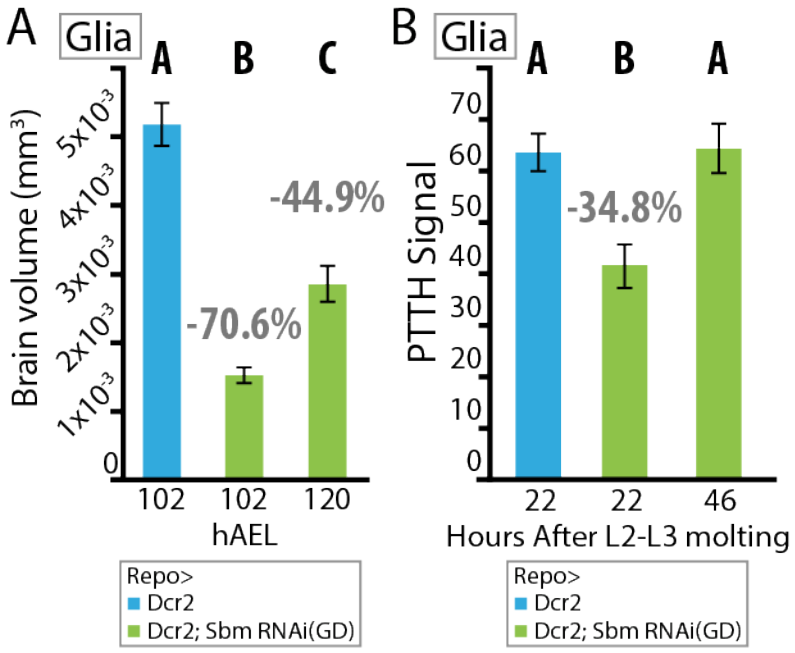

FIGURE S6 Effects of Sbm downregulation in glia on larval brain size and PTTH somatic levels using a second RNAi line. Related to Figure 3 and 6. (A) Expression of a second RNAi line combined with Dcr2 (Dcr2; SbmRNAi(GD)) also produces a reduction in brain size (estimated volume of a single brain lobe in mm^3) relative to controls (Dcr2) when expressed in glia (hAEL, hours after egg laying) (One-Way ANOVA, $p<0.0001$, $F=101.6$; bars with different letters signify that they are significantly different with $p<0.05$ in a Tukey post-test; different letters, $p<0.05$; error bars, S.E.M.; n=14-18). (B) PTTH levels in the somas of PTTH neurons at different time points in third-instar larvae synchronized at the second to third instar molt, upon downregulation of *sbm* in glia (Repo-Gal4) (Kruskal-Wallis, bars with different letters signify that they are significantly different, $p<0.05$; error bars, S.E.M.; n=38-40).

TABLE S1: list of all oligonucleotides used, related to the STAR METHODS (METHOD DETAILS, sbm transcript recognition, cloning and tagging, Sbm GAL4 and UAS-Sbm cloning)

Diagnostic primers for *sbm* transcripts		
RA-RD	F	CGCTTTCATTCTCTCGCAGT
RA-RD	R	GTGTAGCGCATCACGATCAG
RD	F	TATGTCCATGTGCGTCGTCT
RD	R	GTGTAGCGCATCACGATCAG
RC	F	CGACCATCTTGACCACACC
RC	R	TCTGGCATACGCTGAACTTG
RB	F	CCGCACTCCTTTCAGAACAT
RB	R	TTGAGATGGCTTCAACACCA

Cloning primers for *sbm* transcripts		
RB-RC	F	ATGCGCGATAAAATATCACAACT
RB-RC	R	GGGTATCCCCTTCACTCGAA
RA-RD	F	ATGTATCAGCATGTCCAACCA
RA-RD	R	GGGTATCCCCTTCACTCGAA
RD	F	ATGTATCAGCATGTCCAACCA
RD	R	GAGCAGGGCATAGAGATACTCA

Cloning primers for FLAG tagging		
FLAG-RA	F	CAAC**ATG**<u>GACTACAAGGACGACGATGACAAG</u>TATCAGCATGTCCAACCA
FLAG-RB	F	CAAC**ATG**<u>GACTACAAGGACGACGATGACAAG</u>CGCGATAAAATATCACAACT
FLAG-RC	F	CAAC**ATG**<u>GACTACAAGGACGACGATGACAAG</u>CGCGATAAAATATCACAACT
FLAG-RD	F	CAAC**ATG**<u>GACTACAAGGACGACGATGACAAG</u>TATCAGCATGTCCAACCA

Cloning primers for HA tagging		
CD98hc-HA	R	**TTA**<u>AGCGTAATCTGGAACATCGTATGGGTA</u>***TCCACCGCC***CTCCGCCTTCACTGGCA
CD98hc	F	CAACATGGTCCAGGATGAGAAGG

QPCR		
dilp2	F	ACGAGGTGCTGAGTATGGTGTGCG
dilp2	R	CACTTCGCAGCGGTTCCGATATCG
dilp5	F	TGTTCGCCAAACGAGGCACCTTGG
dilp5	R	CACGATTTGCGGCAACAGGAGTCG
dilp6	F	TGCTAGTCCTGGCCACCTTGTTCG
dilp6	R	GGAAATACATCGCCAAGGGCCACC
thor	F	TGATCACCAGGAAGGTTGTCATCTC

thor	R	GAGCCACGGAGATTCTTCATGAAAG
spok	F	TATCTCTTGGGCACACTCGCTG
spok	R	TATCTCTTGGGCACACTCGCTG
nvd	F	GGAAGCGTTGCTGACGACTGTG
nvd	R	TAAAGCCGTCCACTTCCTGCGA
sbm	F	GAATGACGCCGAGATTTATGA
sbm	R	ATGGCCATCGATGAGGTG
rpl29	F	GAACAAGAAGGCCCATCGTA
rpl29	R	AGTAAACAGGCTTTGGCTTGC
