# Supplementary material for: Sobremesa L-type Amino Acid Transporter Expressed in Glia Is Essential for Proper Timing of Development and Brain Growth
Source: Cell Rep. 2018 Sep 18;24(12):3156–3166.e4. doi: 10.1016/j.celrep.2018.08.067 (PMC6167638; doi:10.1016/j.celrep.2018.08.067)
Supplement: Document S2. Article plus Supplemental Information [file mmc2.pdf]

Cell Reports

Article

Sobremesa L-type Amino Acid Transporter Expressed in Glia Is Essential for Proper Timing of Development and Brain Growth

Graphical Abstract

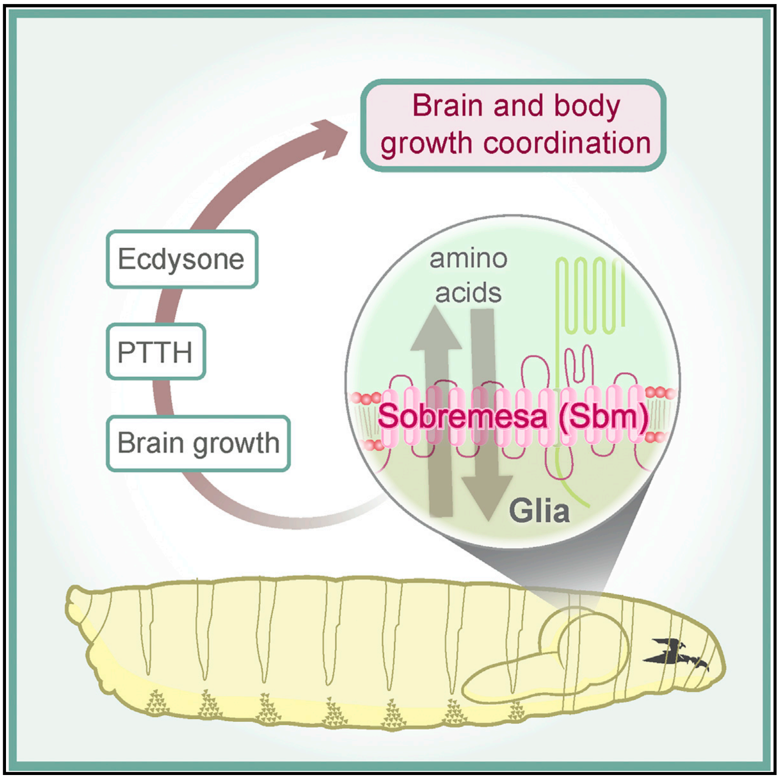

Highlights

- Glia express the SLC7 amino acid transporter Sobremesa, which controls development
- Sobremesa downregulation in glia leads to contrasting effects: small brain and big body size
- Sobremesa downregulation results in reduced ecdysone production
- Sobremesa downregulation causes a delayed rise in PTTH

Authors

Diego Galagovsky, Ana Depetris-Chauvin, Gérard Manière, ..., Elodie Noirot, Georges Alves, Yael Grosjean

Correspondence

yael.grosjean@u-bourgogne.fr

In Brief

Galagovsky et al. reveal a molecular and cellular pathway linking metabolism to developmental growth control. This pathway governs body and nervous system growth timing and involves an amino acid transporter expressed in glia.

Galagovsky et al., 2018, Cell Reports *24*, 3156–3166
September 18, 2018 © 2018 The Authors.
https://doi.org/10.1016/j.celrep.2018.08.067

CellPress

OPEN ACCESS
CellPress

Cell Reports
Article

Sobremesa L-type Amino Acid Transporter Expressed in Glia Is Essential for Proper Timing of Development and Brain Growth

Diego Galagovsky,[1] Ana Depetris-Chauvin,[1,3] Gérard Manière,[1] Flore Geillon,[1] Martine Berthelot-Grosjean,[1] Elodie Noirot,[2] Georges Alves,[1] and Yael Grosjean[1,4,*]
[1]Centre des Sciences du Goût et de l'Alimentation, AgroSup Dijon, CNRS, INRA, Université Bourgogne Franche-Comté, 21000 Dijon, France
[2]Plateforme DImaCell, INRA, Université Bourgogne Franche-Comté, 21000 Dijon, France
[3]Department of Evolutionary Neuroethology, Max Planck Institute for Chemical Ecology, 07745 Jena, Germany
[4]Lead Contact
*Correspondence: yael.grosjean@u-bourgogne.fr
https://doi.org/10.1016/j.celrep.2018.08.067

SUMMARY

In *Drosophila*, ecdysone hormone levels determine the timing of larval development. Its production is regulated by the stereotypical rise in prothoracicotropic hormone (PTTH) levels. Additionally, ecdysone levels can also be modulated by nutrition (specifically by amino acids) through their action on *Drosophila* insulin-like peptides (Dilps). Moreover, in glia, amino-acid-sensitive production of Dilps regulates brain development. In this work, we describe the function of an SLC7 amino acid transporter, Sobremesa (Sbm). Larvae with reduced Sbm levels in glia remain in third instar for an additional 24 hr. These larvae show reduced brain growth with increased body size but do not show reduction in insulin signaling or production. Interestingly, Sbm downregulation in glia leads to reduced Ecdysone production and a surprising delay in the rise of PTTH levels. Our work highlights Sbm as a modulator of both brain development and the timing of larval development via an amino-acid-sensitive and Dilp-independent function of glia.

INTRODUCTION

Members of the SLC7 family of proteins are membrane-bound transporters dedicated to the passage of amino acids. They are classified into two subfamilies: cationic amino acid transporters (CATs), facilitating diffusion of cationic amino acids, and L-type amino acid transporters (LATs), which are mostly specific exchangers. CATs act as single molecules, while LATs form heterodimeric amino acid transporters (HATs) together with members of the SLC3 family of proteins. The HAT is formed through a disulfide bridge between the LAT (12 transmembrane domain light subunit) and the SLC3 subunit (heavy subunit), involving a cysteine positioned in the external loop between the III and IV transmembrane domains of the LAT (Fotiadis et al., 2013; Verrey et al., 2004). Based on the structural characteristics of these proteins, bioinformatics analysis of the *Drosophila melanogaster* genome identified five genes that would code for proteins with LAT characteristics, namely *genderblind*, *minidiscs*, *JhI-21*, *CG1607*, and *CG9413* (Augustin et al., 2007; Reynolds et al., 2009). While the expression and function of a *D. melanogaster* CAT transporter Slimfast has been extensively studied (Bjordal et al., 2014; Cheng et al., 2011; Colombani et al., 2003; Okamoto and Nishimura, 2015; Shim et al., 2012), LATs have been a subject of less scrutiny, and not all five LATs have been functionally characterized. In the present study, we focus on CG9413, a yet-undescribed putative *Drosophila* LAT.

SLC7 amino acid transporters can have a potentially crucial role impacting the physiology of an organism. They can serve the uptake of amino acids to directly feed the protein synthesis machinery or impact the reuptake of amino acids in excretory organs such as the gut and kidneys (Palacín et al., 2005). Related to this, they can feed amino acids into signaling pathways that control the production, release, or response to hormones that regulate amino-acid-sensitive processes such as growth, developmental timing, or feeding, thus functioning as amino acid sensors (Fotiadis et al., 2013; Miguel-Aliaga, 2012).

Through amino acid sensors, the nutritional value of food is coupled to the hormonal signals that coordinate growth and developmental timing. In *Drosophila*, insulin-like peptides (Dilps) activate the insulin-like signaling (IIS) pathway in target tissues and induce cellular growth and proliferation (Edgar, 2006; Mirth and Riddiford, 2007). Their production and release depends on both local and remote signals from nutrient sensors that assess the nutritional status of the organism (Delanoue et al., 2016; Géminard et al., 2009; Okamoto and Nishimura, 2015; Rajan and Perrimon, 2012). Developmental timing, determined by rises of the molting hormone ecdysone and the stereotyped rise in the prothoracicotropic hormone (PTTH) (McBrayer et al., 2007), is also coupled to nutrition through Dilp signaling (Colombani et al., 2005; Mirth et al., 2005). CAT Slimfast functions as a general amino acid transporter and, in that capacity, as a nutrient sensor (Colombani et al., 2003). It is expressed in important organs for growth control, such as in fat body and the CNS, and feeds amino acids into the signaling pathways that ultimately impact the production and release of Dilps (Bjordal et al., 2014; Cheng et al., 2011; Colombani et al., 2003; Okamoto and Nishimura, 2015). Interestingly, LAT-1 Minidiscs and JhI-21 have recently

 © 2018 The Authors.
This is an open access article under the CC BY license (http://creativecommons.org/licenses/by/4.0/).

been shown to function as leucine sensors in the insulin-producing cells of the larva, thus having a role in the regulation of growth and metabolism (Manière et al., 2016; Ziegler et al., 2018).

In addition to their role as amino acid sensors, SLC7 transporters can also impact neurotransmission, since they can serve in the maintenance of the concentrations of specific amino acids that function as neurotransmitters or as precursors for neurotransmitters (Featherstone, 2011). In the case of LATs, Genderblind is expressed in some glia surrounding glutamatergic neurons, where it regulates the level of extracellular glutamate (Augustin et al., 2007; Piyankarage et al., 2008). Furthermore, Genderblind and also JhI-21 control ionotropic glutamate receptor (iGluR) clustering at the neuromuscular junction (NMJ) (Augustin et al., 2007; Ziegler et al., 2016), implicating an essential role for both LATs in neurotransmission. Moreover, *genderblind* disruption in adults impacts pheromonal perception in males and food odor detection in both sexes (Grosjean et al., 2008).

In the present study, we focus on CG9413, one of the less-studied *Drosophila* putative LATs. We show evidence supporting its capacity to form a HAT together with the *Drosophila* SLC3 protein CD98hc. We confirm that it is expressed in the gut and in the CNS of larvae. Moreover, we reveal that expression of CG9413, here named *sobremesa* (*sbm*), is crucial for proper larval growth, feeding, and timing of pupation. Most importantly, *sbm* expression in glia is a key factor for proper timing of development and brain growth. Sbm glial knockdown provokes a reduction in PTTH somatic levels, revealing an unknown glial function and a possible axis of amino-acid-sensitive communication between glia and PTTH-producing cells.

RESULTS

Sobremesa (CG9413 - Sbm) Shows Characteristics of an SLC7 Amino Acid Transporter

To study *sbm* gene expression in *D. melanogaster*, we performed diagnostic PCRs over total cDNA from w^{1118} control flies (Figure S1A), followed by cloning and sequencing of CDSs. We confirmed the existence and predicted sequences listed in FlyBase of its 4 transcripts: *sbm*-RA, RB, RC, and RD. Sbm-PA and PB would have a structure that could interact with the putative *Drosophila* SLC3 protein, the HAT heavy subunit CD98hc. On the contrary, Sbm-PC and PD would be shorter proteins with atypical cysteine locations possibly not compatible for this interaction (Figure 1A).

To visualize these possible physical interactions, we made tagged versions of the different isoforms. CD98hc fused to hemagglutinin (CD98hc:HA) expressed alone in S2 cells appears localized in part to the plasma membrane and provokes membrane extensions resembling filopodia and lamelipodia (Figures 1D and S1C). FLAG:Sbm PA and PB expressed alone also show this localization, while PC and PD localize mainly in big clumps inside the cell or near the cell surface. None of the FLAG:Sbm variants provoke any change in the normal morphology of S2 cells (Figure 1B). Co-expression of CD98hc:HA with either FLAG:Sbm PA or PB showed colocalization of FLAG:Sbm variants with CD98hc:HA at the plasma membrane, supporting the hypothetical interaction between the two proteins (Figure 1C). Western blots revealed either with FLAG or HA antibodies show a band at the expected size for the heterodimer. Breaking the disulfide bridges by adding a reducing agent (DTT) effectively abolished the heterodimer band, displaying clear bands corresponding to the monomers (Figures 1E and S1B). On the contrary, co-expression of PC or PD FLAG:Sbm and CD98hc:HA variants leads to a localization in dots inside the cell with no visible colocalization of these 2 subunits and does not affect cell morphology (Figure 1C). Furthermore, FLAG:Sbm-PC and PD do not produce the dimer size bands expected when co-expressed with CD98hc:HA (Figure S1B). These results strongly suggest that Sbm-PA and PB are able to interact with CD98hc to form a heterodimer at the plasma membrane, while the shorter proteins (PC and PD) would not form a heterodimer with CD98hc and may even interfere with its localization. The interaction between Sbm and CD98hc constitutes important evidence to confirm Sbm as an SLC7A family member and also that they form a complex, which potentially functions as a HAT to transport amino acids.

sbm Expression Pattern

To explore *sbm* expression pattern in further detail at the larval stage, we constructed a Sbm-Gal4 line and produced an antibody that would detect all Sbm proteins. *sbm* is expressed in the CNS, both in glia and neurons (Figures 1F, 1G, and S1D–S1G), and the gut (Figures 1H and S1H). Distinct signal can be seen in subperineurial and cortex glia (Figures 1F, S2A, and S2B), but not in other glial subtypes (Figures S2C–S2F), and in a cluster of four neurons in each brain hemisphere that project to the corpora cardiaca in the ring gland (Figures 1G and S1D–S1F). The expression pattern observed with an antibody against Sbm is supported by that observed with the Gal4 line (Figures S1F–S1H). Noteworthy, the antibody signal appears localized in part to the membrane of the cells (Figures 1F, 1G, S2A, and S2B), supporting the predicted structure and function of the protein as a membrane transporter.

Downregulation of *sbm* in Glia Delays Development and Affects Growth Differentially Among Organs

Since *sbm* is expressed in several larval organs and cell types, we next screened for the effects of *sbm* downregulation in these restricted expression domains. To that end, we drove expression of RNAi in the gut using the myo1D-Gal4 driver line (Figure 2A), panneuronally by combining Appl-Gal4 and Syb-Gal4 lines (Figure 2B) or specifically in the Sbm immunoreactive cluster of neurons that project to the corpora cardiaca with the 18E11-Gal4 line (Figure 2C), and finally in glia with Repo-Gal4 line (Sepp et al., 2001; Yuasa et al., 2003) (Figure 2D). Downregulation with two independent RNAi lines strongly reduces *sbm* mRNA levels to the same extent (Figures S4A and S4B) and significantly decreases the protein levels exclusively in the cellular type where the expression is directed (Figures S3A–S3D). Notoriously, specific downregulation of *sbm* with these two independent RNAi lines in glia provoked a strong developmental delay of almost 24 hr (Figures 2D, S4D, and S5D), while the downregulation of *sbm* on any of the other expression domains did not affect developmental timing (Figures 2A–2C). This phenotype led us to name this gene *sbm*, a Spanish word that translates as "the extended period of time spent at the table conversing long after the meal

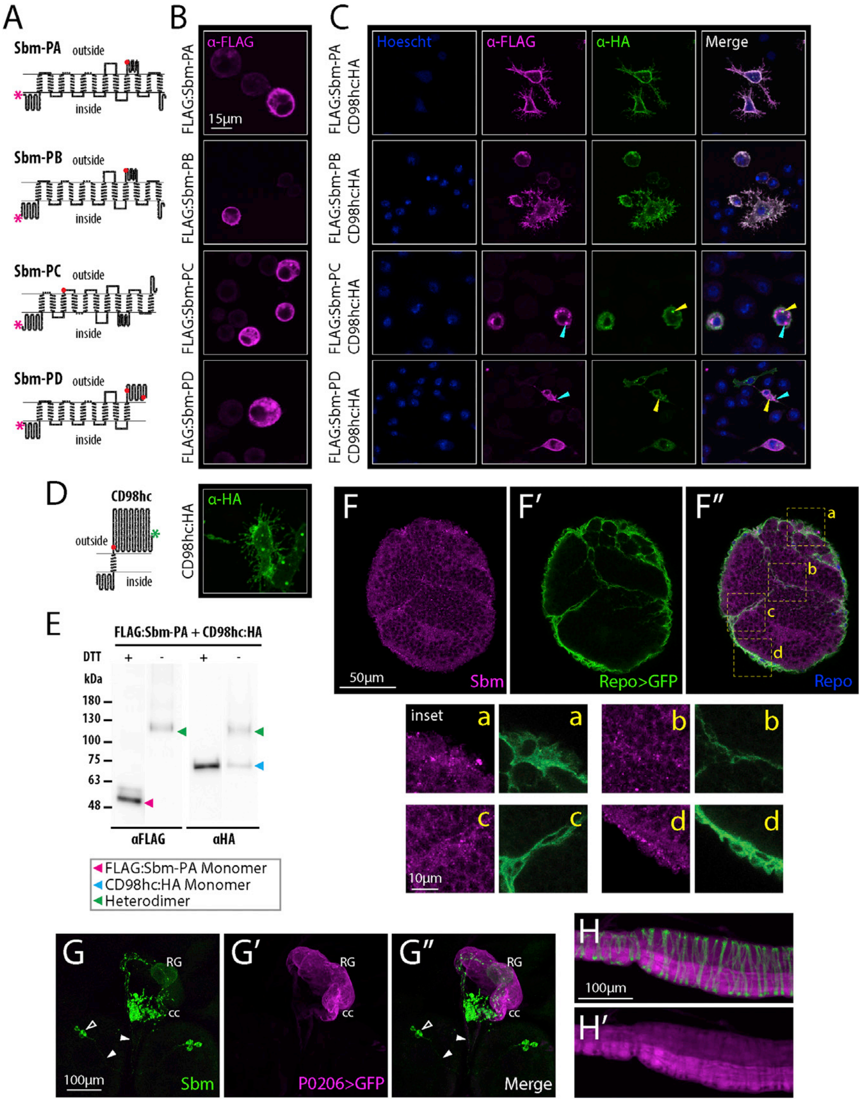

Figure 1. Cellular Localization, Interaction with CD98hc, and Expression Pattern of *sobremesa* (CG9413 – *sbm*)

(A) Putative secondary structure of the translation products of each *sbm* mRNA. The magenta asterisk shows the position of the FLAG tag. Each circle represents an amino acid, and red circles represent extracellular cysteine.

(B) Expression of each FLAG-tagged protein in S2 cells revealed by staining against FLAG (magenta). PA and PB, but not PC and PD, localize in part to the plasma membrane, and PC and PD form clumps inside the cell.

(C) Coexpression of each FLAG:Sbm protein with CD98hc:HA (blue, Hoescht; magenta, FLAG; green, HA) in S2 cells. PA and PB, but not PC and PD, colocalize with CD98hc:HA (cyan and yellow arrowheads for FLAG and HA signal, respectively). PC and PD alter CD98hc localization and abolish the membrane extensions phenotype provoked by CD98hc expression.

(D) Putative structure of CD98hc (left) and immunostaining of S2 cells expressing CD98hc:HA depicting the membrane extension phenotype (right). The green asterisk shows the position of the HA tag on CD98hc.

(E) Western blot of coexpressing FLAG:Sbm-PA and CD98hc:HA cell extracts in the presence (+) and absence (−) of reducing agent (DTT), revealed with anti-FLAG and anti-HA antibodies. The expected position of the heterodimer and monomer bands are pointed based on the molecular weight marker. Heterodimer bands are revealed in the absence of DTT and disappear with DTT, while monomer bands behave in the opposite way (expected molecular weights: Sbm-PA, 57.6 kDA; CD98hc, 62.7 kDa).

(F) Immunostaining in larval brains of Repo-Gal4 > UAS-CD8:GFP reveals expression of Sbm in glia, apparently on the membrane of the subperineurial and cortex glia (Sbm, magenta; GFP, green; Repo, blue; in F″, yellow letters and broken line squares signal the corresponding positions of insets a–d).

(G) Sbm (green) is expressed in a group of neurons (somas, hollow arrowhead; projections, filled arrowheads) projecting to the corpora cardiaca (cc) portion of the ring gland (RG). Counterstaining against GFP (magenta) in a P0206-Gal4 > UAS-CD8:GFP line delimits the RG.

(H) Immunostaining against Sbm (magenta) shows the expression of Sbm in part of the hindgut; counterstaining with phalloidin (green) is shown to delineate the tissue.

has actually ended." This developmental delay was specific to the third larval instar, since molting from the second to the third instar was not delayed (Figure S4C). Downregulation of Sbm using specific glial subtype drivers failed to reproduce the developmental delay phenotype observed with the general glial driver Repo-Gal4 (Figure S5A), suggesting that *sbm* function could be important in several glial subtypes and that its downregulation in one could be compensated by others. It is interesting to note that a very strong ubiquitous knockdown of the gene with the Tubulin-Gal4 driver provokes an extended third larval instar in which larvae remain in the feeding substrate for up to 10 days and never commence wandering or pupation (Figures S5B and S5C); this suggests that Sbm could have a function in other tissues apart from glia and the CNS.

Surprisingly, despite the developmental delay in Repo > SbmRNAi flies, larval weight was notably increased, especially at 96 hr after egg laying (hAEL), a time corresponding to the last 24 hr of the third larval instar of controls (Figure 2E), resulting in individuals of bigger final size. This was also attested by an increase in pupal volume (Figures 2F and S4E) and adult male weight (Figure 2G), which also had bigger wings composed of bigger and more numerous cells (Figures 2H–2J). Also noteworthy is that food intake and the rate of food incorporation to body mass was increased in Repo > SbmRNAi flies (Figure 2K.) Contrary to the glial knockdown of Sbm, its overexpression does not lead to a very significant developmental delay and has a negative effect on growth (Figures S5D and S5E).

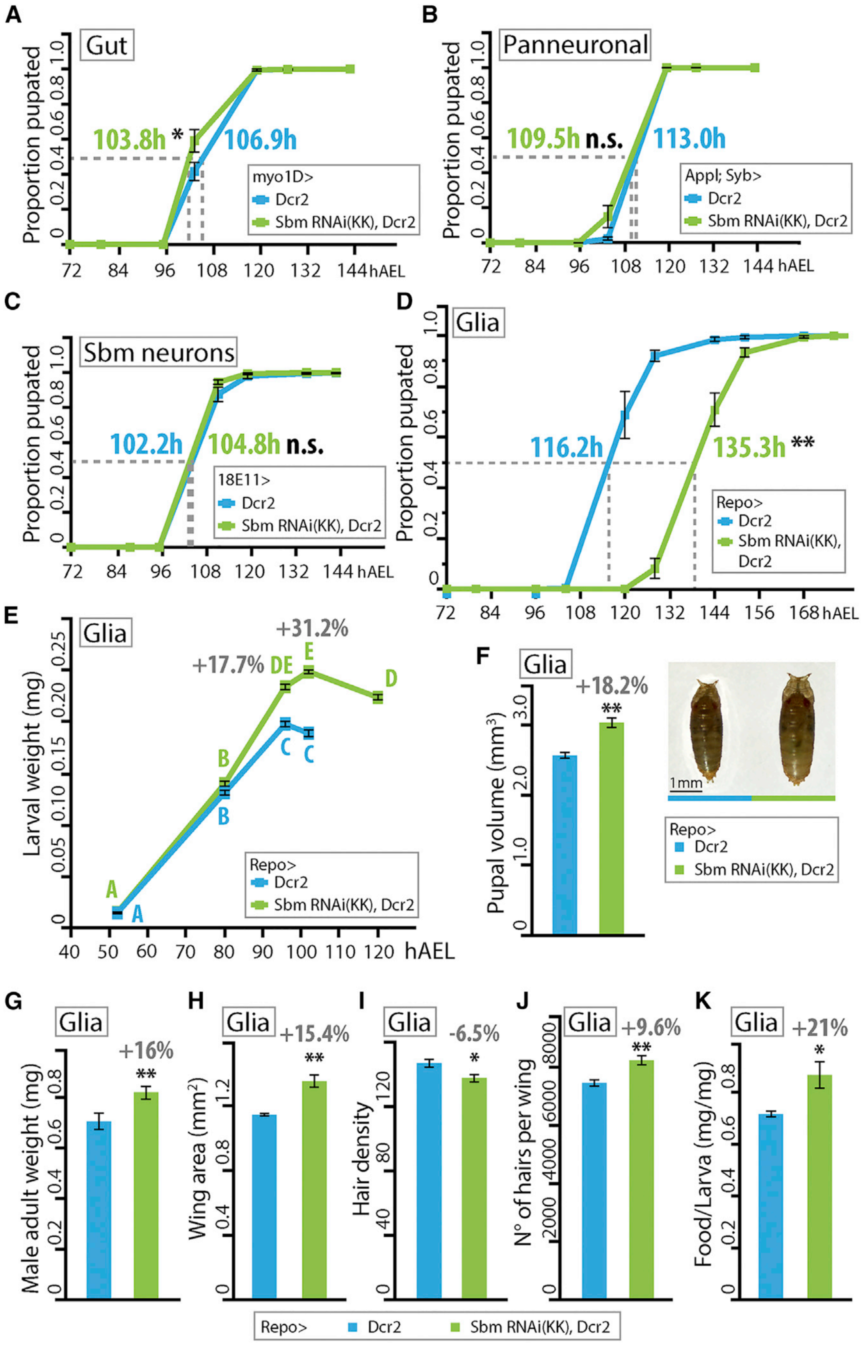

Figure 2. *sbm* Downregulation in Glia Has a Specific Effect on Developmental Timing and Growth

(A–C) Proportion of larvae pupated through time after downregulation of *sbm* in the gut (A; myo1D-Gal4), panneuronally (B; Appl-Gal4; Syb-Gal4), and in the group of neurons that project to the corpora cardiaca (C; 18E11-Gal4). In all cases, a t test was performed, and n.s. indicates nonsignificant differences. t values are as follows: $t = 2.56$ (A, $n = 8$), $t = 1.95$ (B, $n = 9$), and $t = 1.79$ (C, $n = 5$).

(D–K) Downregulation of *sbm* in glia (via expression of the RNAi with Repo-Gal4) leads to a delay to pupation, an increase in final size, and an increase in feeding.

(D) Proportion of larvae pupated through time (t test, $t = 8.07$; $^{**}p < 0.0001$; $n = 15$).

(E) Larval weight (in milligrams) at different time points through development (two-way ANOVA; data were transformed to natural logarithm [ln] to fulfill the test criteria; $p < 0.0001$, $F = 18.99$; points with different letters signify that they are significantly different with $p < 0.05$ in a Tukey post-test; $n = 15–22$ per age and genotype).

(F) Estimated pupal volume (in cubic centimeters) (t test, $t = 6.09$; $^{**}p < 0.0001$; $n = 15$). The picture on the right shows example individuals of each genotype.

(G) Adult male fly weight (mg) (t test, $t = 7.32$; $^{**}p < 0.0001$; $n = 36–65$).

(H) Male fly wing area (mm^2) (t test, $t = 5.51$; $^{**}p < 0.01$; $n = 3$).

(I) Hair density on male fly wings (t test, $t = 2.63$; $^{*}p < 0.05$; $n = 3$).

(J) Estimated total number of hairs per male wing (t test, $t = 4.03$; $^{**}p < 0.01$; $n = 3$).

(K) Amount of food (mg) ingested by 22-hr-old synchronized third-instar larvae normalized by their mass (mg) (t test, $t = 3.28$; $^{*}p < 0.05$; $n = 5$).

In all graphs, data represent mean ± SEM, and the percentages above the bars indicate the difference in respect to the control. hAEL, hours after egg laying.

In order to study if the increased larval weight was a consequence of a general elevated growth rate, we analyzed the size of different organs. We found that not all organs present the same growth rate nor final size differences between controls and Repo > SbmRNAi larvae as the whole animals do. In the case of fat body, an endoreplicative tissue, the cell area behaved similarly to the whole larva, with bigger cells during the early parts of the third larval instar (80 and 96 hAEL), a marked increase in size in the Repo > SbmRNAi larvae relative to controls in the last hours (102 hAEL), and no further rise during the extra time before pupation (120 hAEL) (Figure 3 A). Conversely, the wing imaginal disc area was always significantly smaller in RNAi-treated larvae (a diploid tissue) than in controls, though growth rates were similar (80–102 hAEL). Nevertheless, since the growth rate was maintained during the extra hours they spend as larvae, the final size of the imaginal disc is bigger (120 hAEL), which could in turn account for the bigger wings measured in adults (Figures 3B and 2H). Finally, to our surprise, we observed that the brains of Repo > SbmRNAi larvae were much smaller than that of controls. Even though second-instar larvae do not show statistically significant differences in estimated brain lobe volume (52 hAEL) compared to controls, by the first day of the larval third-instar, their brain size has not changed, much resulting in a brain that is 72.2% smaller than that of controls (80 hAEL). This difference in growth rate is further maintained though time

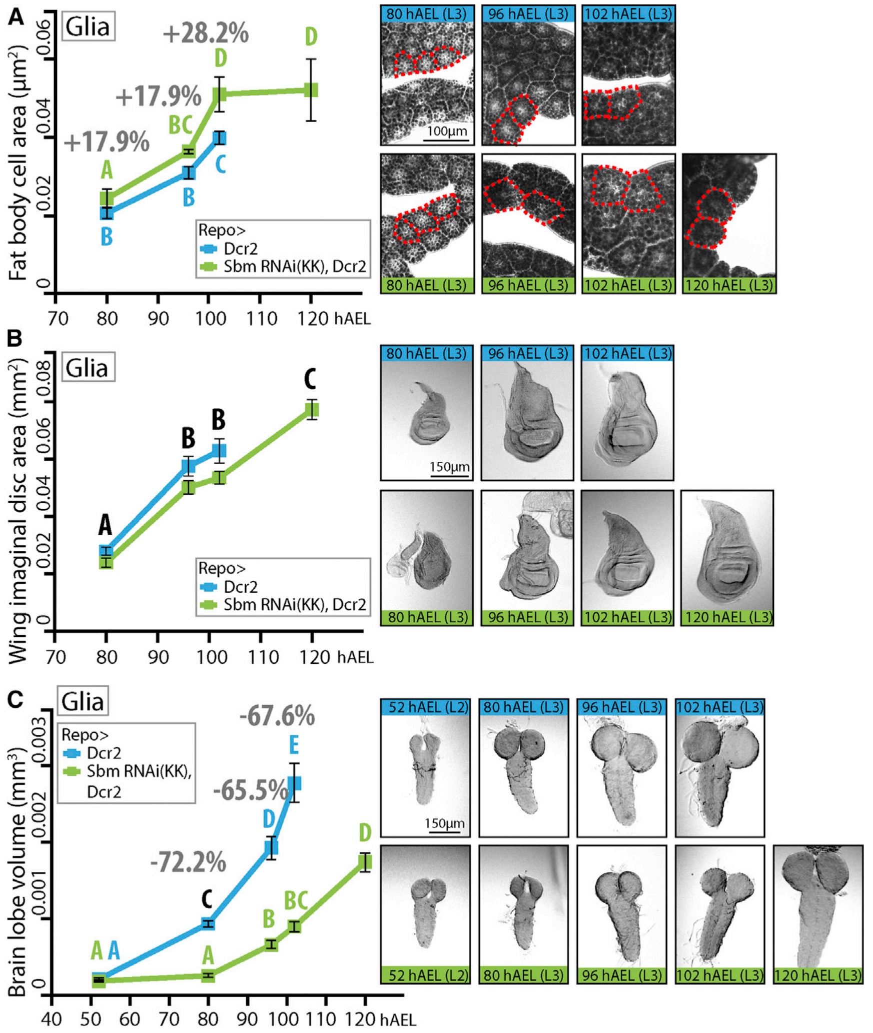

Figure 3. Effects of *sbm* Downregulation in Glia on Various Tissue Growth

(A) Fat body cell area (μm^2) through time and representative pictures of fat bodies stained with Sudan black to show fat accumulation (the red broken line exemplifies some measured cells in each picture) (two-way ANOVA and Duncan post-test, $p < 0.01$, $F = 6.26$; $n = 4–5$.)

(B) Wing imaginal disc area (mm^2) through time and representative pictures (Two-Way ANOVA with a Duncan Post-Test, interaction between the variables time and genotype is not significant so only statistical differences between time points is shown in the graph, $p < 0.0001$, $F = 123.13$; $n = 11–15$).

(C) Estimation of larval single brain lobe volume (mm^3) through time and representative pictures (two-way ANOVA with a Tukey post-test, $p < 0.0001$, $F = 22.55$; DMS = 0.34; $n = 4–5$.)

In all statistical analyses, data were transformed to natural logarithm (ln) to fulfill the test criteria. In all graphs, points with different letters signify that they are significantly different ($p < 0.05$). Data represent mean ± SEM; the percentages above points indicate the difference in respect to the control. hAEL, hours after egg laying.

(96 and 102 hAEL), and while Repo > SbmRNAi larvae spend almost 24 extra hours eating and growing (120 hAEL), their brain never reaches the final control size (Figures 3C and S6A). Observations of the anatomical features (Dearborn and Kunes, 2004; Ngo et al., 2017) of the brain of the Sbm-knockdown larvae through these time points show that brain development is delayed compared to controls (Figure 4A). This is also highlighted by the differences in the number of dividing cells. We found that Repo > SbmRNAi larvae have significantly fewer proliferating cells than controls at both 96 and 120 hAEL. This has negative consequences, at least on the number of glial cells, which are significantly less numerous in Repo > SbmRNAi larvae at 120 hAEL, but not at 96 hAEL (Figures 4B and 4C).

To sum up, reduced levels of Sbm in glia produce a developmental delay that results in heavier larvae, bigger wing imaginal discs, and bigger fat body cells but a smaller brain, possibly due to a decrease in cell proliferation with delayed consequences on neuron and glial cell numbers. Extension of the larval stage is accompanied by larvae staying longer in the food, resulting in bigger pupae and bigger and heavier adult flies.

Downregulation of *sbm* in Dilp6-Expressing Glia Leads to Developmental Delay, but General Glial Knockdown Does Not Lead to Expected Effects of Dilp6 Deficiency

Larval growth is primarily regulated by eight insulin-like peptides (Dilp1–8). Since they all act through a single insulin receptor, regulation of growth is achieved through the spatial, temporal, and circumstantial regulation of their production and release. One such peptide, Dilp6, is produced by glial cells in a nutrient-sensitive manner and regulates brain growth (Okamoto and Nishimura, 2015; Sousa-Nunes et al., 2011).

This led us to explore if the downregulation of *sbm* in Dilp6-producing glia could reproduce the developmental-delay phenotype observed with the panglial driver Repo. Expression of Sbm RNAi with a Dilp6-Gal4 line (which drives expression in glial cells of the subperineurial glia and others [possibly the cortex glia (Okamoto and Nishimura, 2015) and fat body]) significantly delayed the onset of pupation by ~11 hr (Figure 5A), a slighter effect than with the general glial driver. This delay was fully rescued when Gal4 activity was blocked in glial cells co-expressing Gal80, indicating that the observed developmental delay in Dilp6 > SbmRNAi larvae depends on deregulation of Sbm in Dilp6+ glial cells. (Figure 5A). Plus, larvae expressing the RNAi in the fat body did not display a pupation time statistically different from controls (Figure 5B).

Amino acids enter glial cells via CAT transporter Slif, which functions as an amino acid sensor, activating the TOR signaling pathway and positively regulating *dilp6* expression (Okamoto

OPEN ACCESS
CellPress

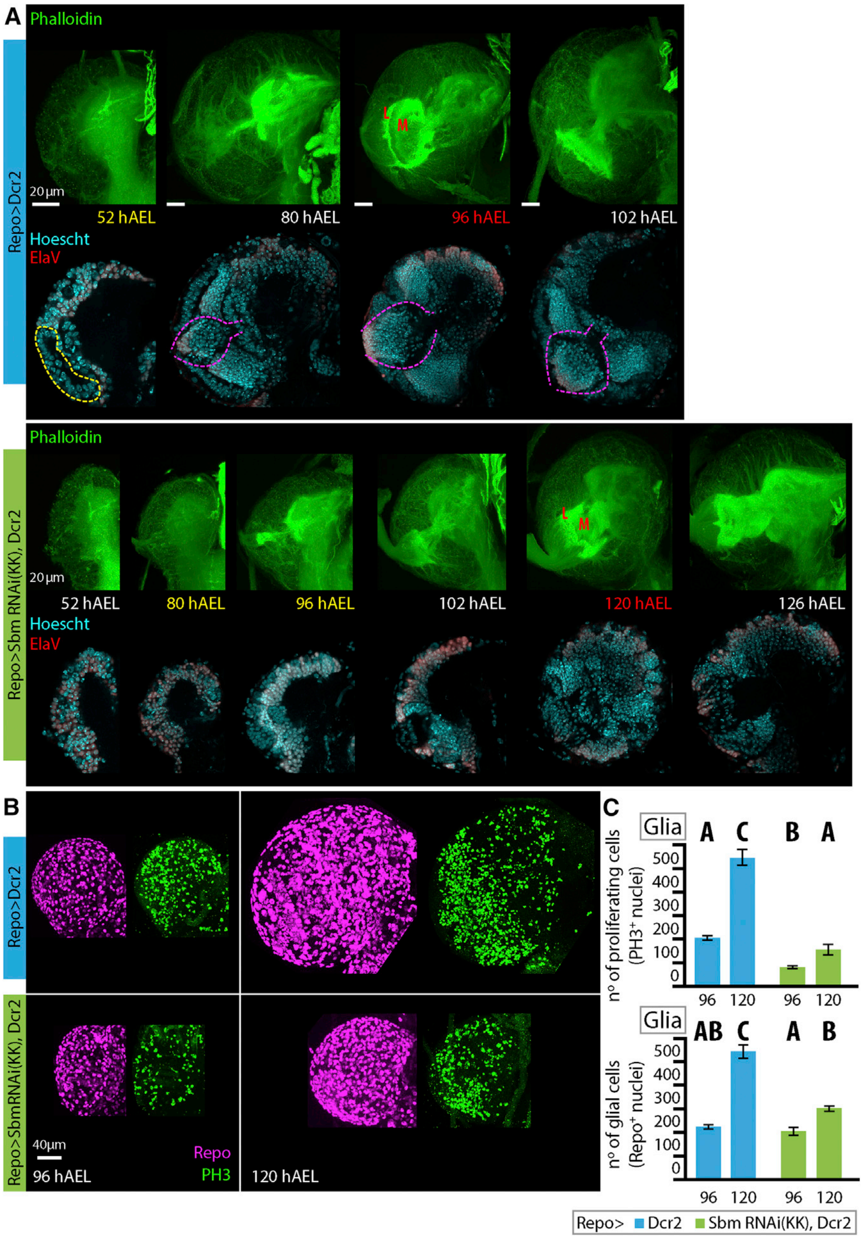

Figure 4. Effects of Sbm Downregulation in Glia on Larval Brain Size, Development, and Brain Cell Proliferation

(A) Observation of the formation of the optic lobe and its neuropils by staining brains with phalloidin (green, cell membranes) and Hoescht (blue, nuclei) and neurons (ElaV, red) at different time points through larval development (hAEL, hours after egg laying) shows that Sbm downregulation in glia produces a delay in the development of larval brain structures. In control brains (top two rows), a group of ElaV-negative (red) cells is observed at 52 hAEL (region marked by broken yellow line) in glia Sbm RNAi-treated larvae (bottom two rows); this is not observed earlier than the mid-third-instar larval stage (80–96 hAEL). In control brains, optic lobe formation is already evident by the early third-instar (80 hAEL, broken yellow lines), and the optic lobe (L) and medulla (M) neuropils are already formed in mid-third-instar larvae (96 hAEL). In Sbm RNAi-treated larvae, optic lobe formation seems to occur between 96 and 102 hAEL, and neuropils are seen at 120 hAEL. It is important to note that while structures form, they do not seem to have the regularity observed in controls.

(B) Representative pictures showing proliferating cells (PH3, green) and glial cells (Repo, magenta) in the brain of control Repo > Dcr2 (top row) and Repo > SbmRNAi Dcr2 (bottom row) larvae at 96 and 120 hAEL.

(C) Quantification of the number of proliferating cells (top) and the number of glial cells (bottom) (two-way ANOVA and Duncan post-test, $p < 0.0001$, $F = 34.33$ and $F = 37.96$; $n = 3–6$). Points with different letters signify that they are significantly different ($p < 0.05$); data represent mean ± SEM. hAEL, hours after egg laying.

and Nishimura, 2015; Sousa-Nunes et al., 2011). If Sbm also functions as an amino acid sensor, then its downregulation should produce effects similar to those caused by a the lack of Slif (i.e., decreased *dilp6* expression and reduced activation of the IIS pathway [target of Dilp6] in the whole brain and reduced production of Dilp5 in insulin-producing cells [IPCs] of the brain; Okamoto and Nishimura, 2015). Contrary to this prediction, the expression level of *dilp6* or the direct target of the IIS pathway, *thor*, were not affected in Repo > SbmRNAi larvae, meaning that the pathway is equally active in the brain of RNAi-treated larvae and controls (Figure 5C). Moreover, the mRNA levels of the two Dilps produced in IPCs, *dilp5* and *dilp2*, were almost doubled in the Repo > SbmRNAi larvae (Figure 5D). It has been described that levels of the Dilp2 immunoreactive signal are inversely correlated with its release into the circulation from the producing cells (Géminard et al., 2009). We found that Repo > SbmRNAi larvae show a lower immunoreactive Dilp2 signal in IPCs (Figure 5E), suggesting that the release of this peptide is augmented in Repo > SbmRNAi larvae.

In conclusion, Sbm functions in Dilp6+ glia, but a reduction of Sbm levels in all glia does not act negatively on *dilp6* expression and function. This suggests that Sbm in glial cells could regulate developmental timing and growth through a different effector.

sbm Downregulation in Glia Reduces the Levels of Key Hormones that Control Developmental Timing and Growth

In *D. melanogaster*, larval growth rate and the progression of larval stages are determined by levels of the molting hormone 20-ecdysone (20E). PTTH induces the expression of the genes that code for the enzymes responsible for synthesizing 20E in the prothoracic gland (McBrayer et al., 2007; Rewitz et al.,

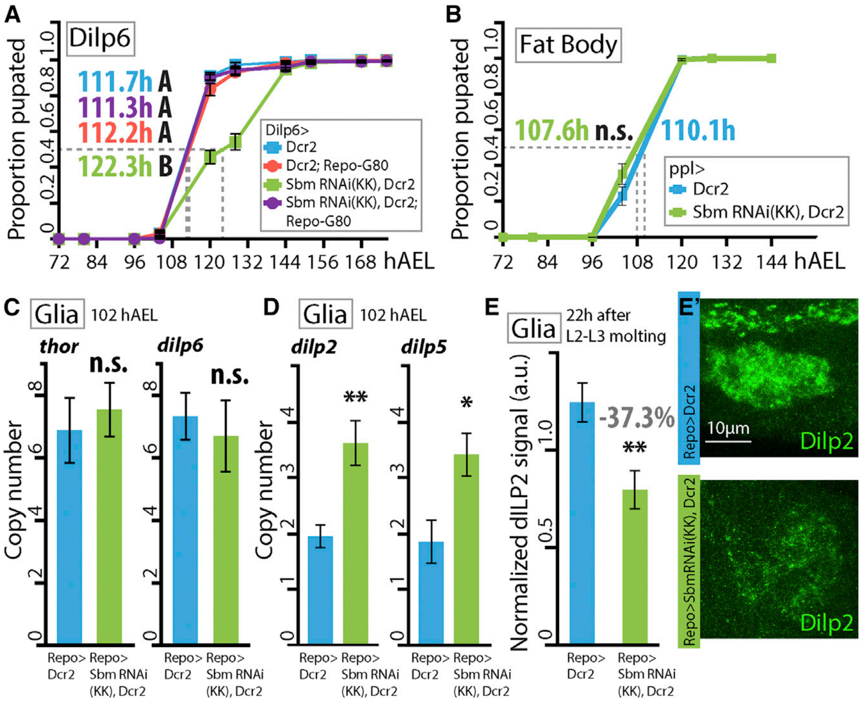

Figure 5. Downregulation of *sbm* in *dilp6*-Expressing Glia Partially Reproduces the Developmental Delay Observed without Affecting *dilp6* Levels

(A) Proportion of larvae pupated through time when overexpressing Dcr2 or SbmRNAi, Dcr2 in *dilp6*-expressing cells (Dilp6-Gal4) either alone or combined with Repo-Gal80 (one-way ANOVA; data were transformed to the reciprocal values to best fulfill the test criteria, $p < 0.0001$, $F = 15.79$; points with different letters signify that they are significantly different [$p < 0.05$] with Duncan post-test; $n = 4–10$).

(B) Idem A but for downregulation of *sbm* in the fat body (ppl-Gal4) (t-Test, $t = 1.29$; $n = 4$).

(C and D) Transcription levels of *thor* and *dilp6* (C) and *dilp2* and *dilp5* (D) (expressed in copy number) in the brain of 102 hAEL third-instar larvae after downregulation of *sbm* in all glia (Repo-Gal4) (t test, *thor* $t = 0.57$, *dilp6* $t = 0.54$, *dilp2* $t = 4.25$, and *dilp5* $t = 3.25$; $^*p < 0.05$; $^{**}p < 0.001$; $n = 4$).

(E) Dilp2 immunoreactive signal levels in the somas of insulin-producing cells (IPCs) 22 hr after the second- to third-instar molt in control and Repo > SbmRNAi larvae (t test, $t = 3.27$; $^{**}p < 0.001$; $n = 28$). The percentage in the graph indicates the difference with respect to controls.

(E′) Representative z stack images of each genotype that illustrate the differences in Dilp2 levels.

In all graphs, error bars represent SEM and n.s. indicates nonsignificant differences.

2009). A good proxy for assessing the levels of ecdysone production are the transcriptional levels of the genes of its synthesis pathway, such as *neverland* and *spookier* (Figure 6A) (McBrayer et al., 2007). We found that in control animals, the transcriptional levels of both genes rise until the moment of wandering, while the levels of these genes are lower in Repo > SbmRNAi larvae than controls at every time point. Most importantly, in Repo > SbmRNAi larvae, *spookier* transcriptional levels never reach the peak levels attained by controls at 37 hr after the L2–L3 molt (Figure 6B). Also, transcript levels of the ecdysone receptor (EcRA), an ecdysone-target gene (Varghese and Cohen, 2007), are reduced in 96 hAEL Repo > SbmRNAi larvae (~24 hr after L2–L3 molt) relative to controls (Figure S6B). Taken together, these results strongly suggest that 20E production in *sbm* mutant larvae is reduced.

Systemic circulating Dilps activate the IIS pathway in the prothoracic gland (PG), a part of the ring gland (RG), increasing its size and increasing 20E levels (Colombani et al., 2005; Mirth et al., 2005). We measured the size of the RG in 102 hAEL larvae and did not find statistically significant differences between Repo > SbmRNAi larvae and controls (Figure 6C). This indicates that the increased Dilp expression and release observed (Figures 5D and 5E) do not significantly affect RG growth; hence, alterations in the transcription of the 20E synthesis genes should not be a consequence of Dilp signaling. Therefore, we hypothesized that alterations on PTTH levels may be responsible for the reduction in the transcription of 20E synthesis genes levels observed.

Because peak levels of PTTH are normally attained a few hours before peak transcriptional levels of ecdysone synthesis genes (McBrayer et al., 2007), we analyzed somatic PTTH levels in the brains of larvae at 22 hr after the L2–L3 molt. Immunoreactive signal levels were significantly reduced in RNAi-treated larvae compared to controls (Figures 6D and S6B). Interestingly, PTTH signal levels in Repo > SbmRNAi larvae at 46 hr after L2–L3 molting (a time point at which control animals have already pupated) were statistically indistinguishable from those of the controls 24 hr earlier (Figures 6D and S6B), indicating that lower levels of Sbm in glia do not abolish the rise in PTTH levels but rather produce a shift in the PTTH peak. This shift is likely the cause of ecdysone in the RG not reaching the levels needed to trigger pupation in the stereotypical timing, leading to the 24-hr prolongation of the larval third-instar observed in these animals and resulting in a phenotype strongly resembling the recently described PTTH-null mutants (Shimell et al., 2018).

DISCUSSION

In this work, we have shown that downregulation of *sbm* in glia provokes negative effects on developmental timing with consequent alterations in growth, and this effect is specific to the larval third-instar. This is associated with a delayed PTTH peak and reduced expression of 20E synthesis and target genes. These larvae have a significantly smaller brain than controls, with fewer proliferating cells and normal levels of dilp6 transcription and activation of the IIS pathway (Figure 7).

Developmental timing and growth are regulated through PTTH and ecdysone. During the larval third-instar in *D. melanogaster*, there is an important developmental checkpoint, the critical weight (CW), after which starvation will no longer delay pupation.

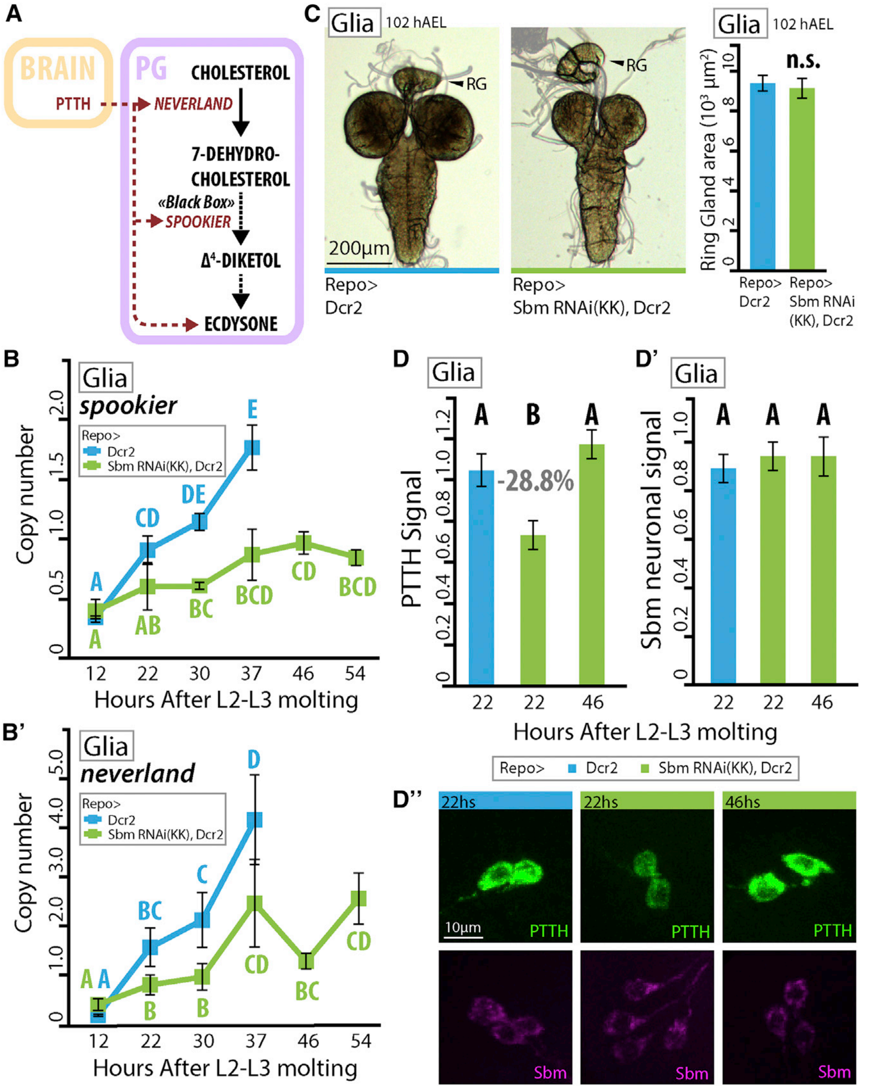

Figure 6. Effects on Developmental Timing Are Likely due to Disruption in PTTH and Ecdysone Levels

(A) As development proceeds, prothoracicotropic hormone (PTTH) is produced in the brain of the larva and released in the prothoracic gland (PG), where it induces the expression of the genes of the molting hormone ecdysone synthesis pathway.

(B and B′) Transcription levels of *spookier* (B) and *neverland* (B′) (expressed in copy number) at different time points in third-instar larvae (L3), synchronized at second- to third-instar molt, upon downregulation of *sbm* in glia (Repo-Gal4) (two-way ANOVA with a Duncan post-test; data were transformed to ln to fulfill the test criteria, $p < 0.05$, $F = 3.12$ and $F = 3.93$; $n = 5$).

(C) Photograph depicting the difference in relative sized between the RG and brain in 102 hAEL larvae (equivalent to ~37 hours after L2–L3 molt) and RG area measurements expressed in 10^3 μm^2 (t test $t = 0.40$; n.s., nonsignificant differences; $n = 11$).

(D and D′) Somatic PTTH (D) and neuronal somatic Sbm levels (D′; as internal control) at different time points in synchronized third-instar larvae upon downregulation of *sbm* in glia (Repo-Gal4). In both cases, one-way ANOVA with a Duncan post-test was used. In (D), $p < 0.05$, $F = 14.46$, $n = 40–46$. In (D′), $p = 0.71$, $F = 3.37$, $n = 40–46$.

(D″) Representative images of somatic PTTH (green) levels and Sbm (magenta) levels in the *sbm*-expressing neurons.

In all graphs, data represent mean ± SEM, and different letters signify groups that are significantly different ($p < 0.05$) in a Duncan post-test.

CW is defined by a rise in the levels of ecdysone, a product of nutrient-dependent Dilp signaling and rising levels of PTTH (Mirth and Riddiford, 2007; Nijhout et al., 2014). Lack of PTTH provokes a shift in the CW and prolongs the growth periods before and after it (McBrayer et al., 2007; Shimell et al., 2018), resulting in bigger larvae. The phenotypes we describe in the present report are consistent with this.

Nature of the Link between Glia and PTTH Neurons

Glial cells regulate the exit from quiescence of neuroblasts in third-instar larval brain (Lanet et al., 2013; Sousa-Nunes et al., 2011), allowing for an increase in proliferation and brain size. This regulation is mediated through two factors produced by glia: Dilp6 and Jellybelly (Jeb) (Cheng et al., 2011; Sousa-Nunes et al., 2011). Dilp6 is regulated at the transcriptional level according to the nutritional status of the larva (Okamoto and Nishimura, 2015; Sousa-Nunes et al., 2011). Jeb is constitutively produced in glia and allows for “brain growth sparing” in nutritionally adverse conditions, permitting the brain to grow at a higher rate than the rest of the body (Cheng et al., 2011). Both factors activate the phosphatidylinositol 3-kinase (PI3K) signaling pathway in quiescent neuroblasts; Dilp6 does it through the insulin receptor, while Jeb does it through the receptor Alk. We observed that brain growth and proliferation in the brain are highly impaired in glial-Sbm-deficient larvae, which could be due to reduced production of either of these two factors. Since our results indicate that *dilp6* transcription does not appear to be affected in this context, future work should focus on Jeb as a likely candidate in mediating the observed phenotypes. We also observed that PI3K pathway activation does not appear to be altered by Sbm glial downregulation. This raises the possibility that another unknown factor is involved, though it remains to be determined whether the release of Dilp6 from glial cells, the temporal dynamics of its transcription, or the temporal dynamics of PI3K pathway activation are affected. In any case, we have shown that glial downregulation of Sbm reduces brain size and that this may be in part due to reduced proliferation, which at least initially does not affect glia itself. This could mean that downstream to the glial signals, neuroblasts communicate with PTTH-producing neurons during their proliferation, allowing for the coordination of the

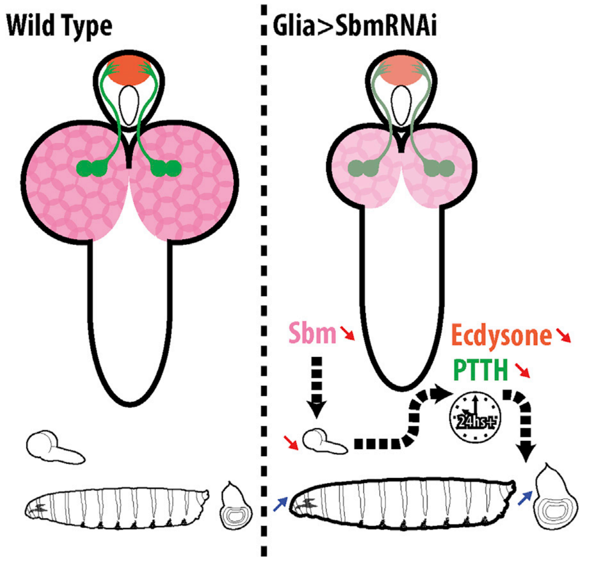

Figure 7. Model

Sbm is expressed in glia of the larva (magenta). Downregulation of Sbm in glia provokes a delay in the development of the brain and a reduction in its final size (red arrow); in turn, this impacts the production of PTTH in PTTH neurosecretory cells (green) and leads to a reduction in expression of the genes responsible for the production of ecdysone in the PG (orange). This leads to a delay in the initiation of wandering and pupation, allowing the larvae an extra 24 hr in which tissues such as the represented wing imaginal disc continue growing (blue arrows), resulting in larvae and pupae of an increased size with relatively small brains.

progression of brain development with developmental timing, possibly ensuring that a minimal brain development is achieved before triggering the processes that indefectibly lead to pupation.

It is interesting to note that the recently described PTTH-null mutants (Shimell et al., 2018) show growth phenotypes very similar to the ones described in the present work, strengthening our model (Figure 7): a 24-hr delay to pupation in the larval third-instar without affecting the L2–L3 transition; an increase in larval, pupal, and adult size; a desynchronization of growth of different tissues; and altered transcription of ecdysone genes. More importantly, the PTTH-null mutation does not alter brain size, which adds to the idea that Sbm function in the glia is necessary to regulate brain growth, and this in turn regulates developmental timing (Figure 7).

It is important to add that the relationship between glia and PTTH could potentially be direct and involve close contact between Sbm^{+} glia and PTTH-producing neurons. Imaging and functional experiments should be carried out to explore this possibility.

Cellular Function of Sbm

Whether the link between glia and PTTH neurons is direct or indirect, in both cases, the question that arises is how is the Sbm amino acid antiporter involved? As a LAT, Sbm could act by exchanging specific amino acids between the internal brain medium and glia, regulating in this way their relative concentrations and ensuring proper cellular functions. Transported amino acids could be themselves neurotransmitters or precursors for neurotransmitters. In this regard, Genderblind, another member of the SLC7 family expressed in some glia, has been described to regulate ambient glutamate levels, impacting neuronal physiology and social communication (Augustin et al., 2007; Featherstone, 2011; Grosjean et al., 2008; Piyankarage et al., 2008). Also, the SLC7 antiporter JhI-21 has a similar function in the NMJ (Ziegler et al., 2016). Alternatively, Sbm could exchange amino acids against Na^{+} (Fotiadis et al., 2013) and affect the glial resting membrane potential and, in doing so, alter the Ca^{2+} waves necessary for production and release of Dilp6 (Spéder and Brand, 2014). Lastly, Sbm could be transporting amino acids from the hemolymph and serving to sense the levels of one or more specific amino acids in the glia, as it has been described for Minidiscs in IPCs, which transports leucine (Manière et al., 2016). Further investigations describing Sbm amino acid transport capabilities will help to solve its action on PTTH neurons through glia.

STAR★METHODS

Detailed methods are provided in the online version of this paper and include the following:

- KEY RESOURCES TABLE
- CONTACT FOR REAGENT AND RESOURCE SHARING
- EXPERIMENTAL MODEL AND SUBJECT DETAILS
  - Fly stocks and rearing conditions
  - S2 cell culture
- METHOD DETAILS
  - sbm transcript recognition, cloning and tagging
  - Sbm GAL4 and UAS-Sbm cloning
  - Sbm antibody
  - S2 cell immunocytology and western blots
  - Dissections and Immunostainings
  - Fluorescence quantification
  - Developmental timing, growth assessment and feeding assays
  - Organ size measurement
  - Real Time PCRs
- QUANTIFICATION AND STATISTICAL ANALYSIS

SUPPLEMENTAL INFORMATION

Supplemental Information includes six figures and one table and can be found with this article online at https://doi.org/10.1016/j.celrep.2018.08.067.

ACKNOWLEDGMENTS

We thank the Bloomington Stock Center and the Vienna Drosophila RNAi Center for fly stocks. We acknowledge Christine Arnould (Plateforme DImaCell, INRA, Université Bourgogne Franche-Comté), Aymeric Fabre, Serge Loquin, Cindy Ménagé, Kévin Piffaretti, and José Solonot for their technical assistance. We thank Dr. Bill Hansson and Dr. Markus Knaden of the Max Plank Institute for Chemical Ecology (Jena, Germany) for access to the resources used to perform experiments at their laboratory. We also thank Dr. Nuria Romero of the Institute de Biologie Valrose at Parc Valrose (Nice, France) for discussions about the data related to this article. We thank Pr. David Featherstone and Dr. Anna Ziegler for their assistance and discussions. Research in

Y.G.'s laboratory is supported by the Centre National de la Recherche Scientifique, the Université de Bourgogne Franche-Comté, the Conseil Régional Bourgogne Franche-Comté (PARI grant), the FEDER (European Funding for Regional Economic Development), and the European Council (ERC starting grant GliSFCo-311403).

In memory of Pr. David E. Featherstone.

AUTHOR CONTRIBUTIONS

Conceptualization, D.G. and Y.G.; Methodology, D.G. and Y.G.; Investigation, D.G., A.D.-C., F.G., G.M., M.B.-G., E.N., and G.A.; Writing – Original Draft, D.G. and Y.G., Writing – Review & Editing, D.G., A.D.-C., and Y.G.; Visualization, D.G. and A.D.-C.; Funding Acquisition, Y.G.; Supervision, Y.G.

DECLARATION OF INTERESTS

The authors declare no competing interests.

Received: September 27, 2017
Revised: July 13, 2018
Accepted: August 22, 2018
Published: September 18, 2018

REFERENCES

Augustin, H., Grosjean, Y., Chen, K., Sheng, Q., and Featherstone, D.E. (2007). Nonvesicular release of glutamate by glial xCT transporters suppresses glutamate receptor clustering in vivo. J. Neurosci. *27*, 111–123.

Awasaki, T., Lai, S.-L., Ito, K., and Lee, T. (2008). Organization and postembryonic development of glial cells in the adult central brain of Drosophila. J. Neurosci. *28*, 13742–13753.

Bischof, J., Björklund, M., Furger, E., Schertel, C., Taipale, J., and Basler, K. (2013). A versatile platform for creating a comprehensive UAS-ORFeome library in Drosophila. Development *140*, 2434–2442.

Bjordal, M., Arquier, N., Kniazeff, J., Pin, J.P., and Léopold, P. (2014). Sensing of amino acids in a dopaminergic circuitry promotes rejection of an incomplete diet in Drosophila. Cell *156*, 510–521.

Cheng, L.Y., Bailey, A.P., Leevers, S.J., Ragan, T.J., Driscoll, P.C., and Gould, A.P. (2011). Anaplastic lymphoma kinase spares organ growth during nutrient restriction in Drosophila. Cell *146*, 435–447.

Colombani, J., Raisin, S., Pantalacci, S., Radimerski, T., Montagne, J., and Léopold, P. (2003). A nutrient sensor mechanism controls Drosophila growth. Cell *114*, 739–749.

Colombani, J., Bianchini, L., Layalle, S., Pondeville, E., Dauphin-Villemant, C., Antoniewski, C., Carré, C., Noselli, S., and Léopold, P. (2005). Antagonistic actions of ecdysone and insulins determine final size in Drosophila. Science *310*, 667–670.

Dearborn, R., Jr., and Kunes, S. (2004). An axon scaffold induced by retinal axons directs glia to destinations in the Drosophila optic lobe. Development *131*, 2291–2303.

Delanoue, R., Meschi, E., Agrawal, N., Mauri, A., Tsatskis, Y., McNeill, H., and Léopold, P. (2016). Drosophila insulin release is triggered by adipose Stunted ligand to brain Methuselah receptor. Science *353*, 1553–1556.

Doherty, J., Logan, M.A., Taşdemir, O.E., and Freeman, M.R. (2009). Ensheathing glia function as phagocytes in the adult Drosophila brain. J. Neurosci. *29*, 4768–4781.

Edgar, B.A. (2006). How flies get their size: genetics meets physiology. Nat. Rev. Genet. *7*, 907–916.

Featherstone, D.E. (2011). Glial solute carrier transporters in Drosophila and mice. Glia *59*, 1351–1363.

Fotiadis, D., Kanai, Y., and Palacín, M. (2013). The SLC3 and SLC7 families of amino acid transporters. Mol. Aspects Med. *34*, 139–158.

Galagovsky, D., Katz, M.J., Acevedo, J.M., Sorianello, E., Glavic, A., and Wappner, P. (2014). The Drosophila insulin-degrading enzyme restricts growth by modulating the PI3K pathway in a cell-autonomous manner. Mol. Biol. Cell *25*, 916–924.

Géminard, C., Rulifson, E.J., and Léopold, P. (2009). Remote control of insulin secretion by fat cells in Drosophila. Cell Metab. *10*, 199–207.

Grosjean, Y., Grillet, M., Augustin, H., Ferveur, J.-F., and Featherstone, D.E. (2008). A glial amino-acid transporter controls synapse strength and courtship in Drosophila. Nat. Neurosci. *11*, 54–61.

Lanet, E., Gould, A.P., and Maurange, C. (2013). Protection of neuronal diversity at the expense of neuronal numbers during nutrient restriction in the Drosophila visual system. Cell Rep. *3*, 587–594.

Manière, G., Ziegler, A.B., Geillon, F., Featherstone, D.E., and Grosjean, Y. (2016). Direct sensing of nutrients via a LAT1-like transporter in Drosophila insulin-producing cells. Cell Rep. *17*, 137–148.

McBrayer, Z., Ono, H., Shimell, M., Parvy, J.-P.J.-P.P., Beckstead, R.B., Warren, J.T., Thummel, C.S., Dauphin-Villemant, C., Gilbert, L.I., and O'Connor, M.B. (2007). Prothoracicotropic hormone regulates developmental timing and body size in Drosophila. Dev. Cell *13*, 857–871.

Miguel-Aliaga, I. (2012). Nerveless and gutsy: intestinal nutrient sensing from invertebrates to humans. Semin. Cell Dev. Biol. *23*, 614–620.

Mirth, C.K., and Riddiford, L.M. (2007). Size assessment and growth control: how adult size is determined in insects. BioEssays *29*, 344–355.

Mirth, C., Truman, J.W., and Riddiford, L.M. (2005). The role of the prothoracic gland in determining critical weight for metamorphosis in Drosophila melanogaster. Curr. Biol. *15*, 1796–1807.

Ngo, K.T., Andrade, I., and Hartenstein, V. (2017). Spatio-temporal pattern of neuronal differentiation in the Drosophila visual system: A user's guide to the dynamic morphology of the developing optic lobe. Dev. Biol. *428*, 1–24.

Nijhout, H.F., Riddiford, L.M., Mirth, C., Shingleton, A.W., Suzuki, Y., and Callier, V. (2014). The developmental control of size in insects. Wiley Interdiscip. Rev. Dev. Biol. *3*, 113–134.

Okamoto, N., and Nishimura, T. (2015). Signaling from glia and cholinergic neurons controls nutrient-dependent production of an insulin-like peptide for Drosophila body growth. Dev. Cell *35*, 295–310.

Palacín, M., Nunes, V., Font-Llitjós, M., Jiménez-Vidal, M., Fort, J., Gasol, E., Pineda, M., Feliubadaló, L., Chillarón, J., and Zorzano, A. (2005). The genetics of heteromeric amino acid transporters. Physiology (Bethesda) *20*, 112–124.

Pfeiffer, B.D., Ngo, T.-T.T.B., Hibbard, K.L., Murphy, C., Jenett, A., Truman, J.W., and Rubin, G.M. (2010). Refinement of tools for targeted gene expression in Drosophila. Genetics *186*, 735–755.

Piyankarage, S.C., Augustin, H., Grosjean, Y., Featherstone, D.E., and Shippy, S.A. (2008). Hemolymph amino acid analysis of individual Drosophila larvae. Anal. Chem. *80*, 1201–1207.

Rajan, A., and Perrimon, N. (2012). Drosophila cytokine unpaired 2 regulates physiological homeostasis by remotely controlling insulin secretion. Cell *151*, 123–137.

Rewitz, K.F., Yamanaka, N., Gilbert, L.I., and O'Connor, M.B. (2009). The insect neuropeptide PTTH activates receptor tyrosine kinase torso to initiate metamorphosis. Science *326*, 1403–1405.

Reynolds, B., Roversi, P., Laynes, R., Kazi, S., Boyd, C.A., and Goberdhan, D.C.I. (2009). Drosophila expresses a CD98 transporter with an evolutionarily conserved structure and amino acid-transport properties. Biochem. J. *420*, 363–372.

Sepp, K.J., Schulte, J., and Auld, V.J. (2001). Peripheral glia direct axon guidance across the CNS/PNS transition zone. Dev. Biol. *238*, 47–63.

Shim, J., Mukherjee, T., and Banerjee, U. (2012). Direct sensing of systemic and nutritional signals by haematopoietic progenitors in Drosophila. Nat. Cell Biol. *14*, 394–400.

Shimell, M., Pan, X., Martin, F.A., Ghosh, A.C., Leopold, P., O'Connor, M.B., and Romero, N.M. (2018). Prothoracicotropic hormone modulates environmental adaptive plasticity through the control of developmental timing. Development *145*, dev159699.

Sousa-Nunes, R., Yee, L.L., and Gould, A.P. (2011). Fat cells reactivate quiescent neuroblasts via TOR and glial insulin relays in Drosophila. Nature *471*, 508–512.

Spéder, P., and Brand, A.H. (2014). Gap junction proteins in the blood-brain barrier control nutrient-dependent reactivation of Drosophila neural stem cells. Dev. Cell *30*, 309–321.

Tennessen, J.M., Barry, W.E., Cox, J., and Thummel, C.S. (2014). Methods for studying metabolism in Drosophila. Methods *68*, 105–115.

Varghese, J., and Cohen, S.M. (2007). microRNA miR-14 acts to modulate a positive autoregulatory loop controlling steroid hormone signaling in Drosophila. Genes Dev. *21*, 2277–2282.

Verrey, F., Closs, E.I., Wagner, C.A., Palacin, M., Endou, H., and Kanai, Y. (2004). CATs and HATs: the SLC7 family of amino acid transporters. Pflugers Arch. *447*, 532–542.

Yamanaka, N., Romero, N.M., Martin, F.A., Rewitz, K.F., Sun, M., O'Connor, M.B., and Léopold, P. (2013). Neuroendocrine control of Drosophila larval light preference. Science *341*, 1113–1116.

Yuasa, Y., Okabe, M., Yoshikawa, S., Tabuchi, K., Xiong, W.C., Hiromi, Y., and Okano, H. (2003). Drosophila homeodomain protein REPO controls glial differentiation by cooperating with ETS and BTB transcription factors. Development *130*, 2419–2428.

Ziegler, A.B., Augustin, H., Clark, N.L., Berthelot-Grosjean, M., Simonnet, M.M., Steinert, J.R., Geillon, F., Manière, G., Featherstone, D.E., and Grosjean, Y. (2016). The amino acid transporter JhI-21 coevolves with glutamate receptors, impacts NMJ physiology, and influences locomotor activity in Drosophila larvae. Sci. Rep. *6*, 19692.

Ziegler, A.B., Manière, G., and Grosjean, Y. (2018). JhI-21 plays a role in Drosophila insulin-like peptide release from larval IPCs via leucine transport. Sci. Rep. *8*, 1908.

STAR★METHODS

KEY RESOURCES TABLE

REAGENT or RESOURCE	SOURCE	IDENTIFIER
Antibodies		
Mouse monoclonal Anti-FLAG	Sigma	Cat#F3165; RRID:AB_259529
Rabbit polyclonal Anti-HA	Sigma	Cat#H6908; RRID:AB_260070
Rabbit polyclonal Anti-Phospho-Histone-3	Sigma	Cat# H0412; RRID:AB_477043
Mouse monoclonal Anti-HA	Eurogentec	Cat# AFC-101P; RRID:AB_291231
Rabbit polyclonal Anti-GFP	Thermo Fisher Scientific	Cat# A-11122; RRID:AB_221569
Mouse monoclonal Anti-GFP	Thermo Fisher Scientific	Cat# MA5-15256; RRID:AB_10979281
Mouse monoclonal Anti-ElaV	DSHB	Cat# 9F8A9; RRID:AB_2314364
Mouse monoclonal Anti-Repo	DSHB	Cat# 8D12; RRID:AB_528448
Guinea Pig polyclonal Anti-PTTH	Yamanaka et al., 2013	N/A
Rat polyclonal Anti-Dilp2	Géminard et al., 2009	N/A
Rabbit polyclonal Anti-Sbm	This paper	N/A
Goat polyclonal anti-mouse IgG-HRP	Santa Cruz Biotechnology	Cat# sc-2005; RRID:AB_631736
Goat anti-Rabbit IgG (H+L) Cross-Adsorbed Secondary Antibody, Alexa Fluor 488	Thermo Fisher Scientific	Cat# A11008; RRID:AB_141365
Goat anti-Rabbit IgG (H+L) Cross-Adsorbed Secondary Antibody, Alexa Fluor 594	Thermo Fisher Scientific	Cat# A11012; RRID:AB_2534079
Goat anti-Rat IgG (H+L) Cross-Adsorbed Secondary Antibody, Alexa Fluor 488	Thermo Fisher Scientific	Cat# A-11006; RRID:AB_2534074
Goat anti-Mouse IgG (H+L) Cross-Adsorbed Secondary Antibody, Alexa Fluor 488	Thermo Fisher Scientific	Cat# R37120; RRID:AB_2556548
Goat anti-Guinea Pig IgG (H+L) Highly Cross-Adsorbed Secondary Antibody, Alexa Fluor 488	Thermo Fisher Scientific	Cat# A-11073; RRID:AB_2534117
Goat anti-Mouse IgG (H+L) Cross-Adsorbed Secondary Antibody, Alexa Fluor 594	Thermo Fisher Scientific	Cat# A-11005; RRID:AB_2534073
Goat anti-Mouse IgG1 Cross-Adsorbed Secondary Antibody, Alexa Fluor 633	Thermo Fisher Scientific	Cat# A-21126; RRID:AB_2535768
Experimental Models: Cell Lines		
Drosophila S2 Cells	Thermo Fisher Scientific	Cat# R69007
Experimental Models: Organisms/Strains		
D. melanogaster UAS-Sbm RNAi	Vienna Drosophila Resource Center	VDRC #108867
D. melanogaster UAS-Sbm RNAi(GD)	Vienna Drosophila Resource Center	VDRC #45180
D. melanogaster UAS-Dcr2	Vienna Drosophila Resource Center	VDRC #60009
D. melanogaster AttP2	Kyoto Drosophila Genomics and Genetic Resources	DGGR #36303
D. melanogaster Myo1D-Gal4	Kyoto Drosophila Genomics and Genetic Resources	DGGR #113094
D. melanogaster NP2276-Gal4	Kyoto Drosophila Genomics and Genetic Resources	DGGR #112853
D. melanogaster NP6520-Gal4	Kyoto Drosophila Genomics and Genetic Resources	DGGR #105240
D. melanogaster NP3233-Gal4	Kyoto Drosophila Genomics and Genetic Resources	DGGR #113173
D. melanogaster Dilp6-Gal4	Kyoto Drosophila Genomics and Genetic Resources	DGGR #103877
D. melanogaster Appl-Gal4	Bloomington Drosophila Stock Center	BDSC #32040
D. melanogaster Syb-Gal4	Bloomington Drosophila Stock Center	BDSC #51635
D. melanogaster 18E11-Gal4	Bloomington Drosophila Stock Center	BDSC #45446
D. melanogaster Repo-Gal4	Bloomington Drosophila Stock Center	BDSC #7415
D. melanogaster Ppl-Gal4	Bloomington Drosophila Stock Center	BDSC #58768
D. melanogaster Nrv2-Gal4, UAS-GFP	Bloomington Drosophila Stock Center	BDSC #6794

(*Continued on next page*)

Continued		
REAGENT or RESOURCE	SOURCE	IDENTIFIER
D. melanogaster Tub-Gal4	Bloomington Drosophila Stock Center	BDSC #5138
D. melanogaster UAS-GFP	Bloomington Drosophila Stock Center	BDSC # 5130
D. melanogaster Act5C(FRT.CD2)Gal4	Bloomington Drosophila Stock Center	BDSC #4780
D. melanogaster UAS-FLP	Bloomington Drosophila Stock Center	BDSC #4539 and #8209
D. melanogaster P0206-Gal4	Gift from Dr. Pablo Wappner (Colombani et al., 2005	N/A
D. melanogaster Alrm-Gal4	Gift from Dr. Marc Freeman (Doherty et al., 2009)	N/A
D. melanogaster MZ0709-Gal4	Gift from Dr. Marc Freeman (Doherty et al., 2009)	N/A
D. melanogaster Repo-Gal80	Gift from Dr. Yuh Nung Jan (Awasaki et al., 2008	N/A
D. melanogaster Sbm-Gal4	This paper	N/A
D. melanogaster UAS-SbmPA	This paper	N/A
Oligonucleotides		
Please refer to Table S1.	N/A	N/A
Recombinant DNA		
BACR22I24	BAC PAC Resources at the Children's Hospital Oakland Research Institute	Genebank ID AC022346

CONTACT FOR REAGENT AND RESOURCE SHARING

Further information and requests for resources and reagents should be directed to and will be fulfilled by the lead contact, Yael Grosjean (yael.grosjean@u-bourgogne.fr)

EXPERIMENTAL MODEL AND SUBJECT DETAILS

Fly stocks and rearing conditions

Flies were reared in food containing 6.5% corn flour, 6.5% yeast extract, 1% Agar-Agar and 3% of a 0.1% Tegosept (Apex) antifungal solution in ethanol. Rearing temperature was 25°C for ubiquitous expression experiments. In all other experiments crosses, ovipositions and embryo development were carried at 25°C while development from the 1st-instar stage onward was conducted at 29°C in constant darkness. In all experiments with larvae, animals used were of unknown gender. In experiments with adult flies, only male flies were used to avoid variability due to gender dimorphism affecting size. A complete list of fly stocks is given in the Key Resources Table.

S2 cell culture

Cells were cultured in Schneider's medium (Invitrogen) containing penicillin (50 u/mL), streptomycin (50 μg/mL) and 10% fetal bovine serum (FBS), at 28°C.

METHOD DETAILS

sbm transcript recognition, cloning and tagging

Total RNA was extracted using Isol-RNA Lysis Reagent (5 Prime) and cDNA synthesized using iScript Select cDNA Synthesis Kit (BioRad) with OligodT and a Random Primers (for cloning the different mRNAs) or specific primer (for identification of transcripts). PCR for transcript identification and cloning of different isoforms was done using specific primers listed in Table S1. Amplification products were purified using NucleoSpin Extract II (Macherey Nagel) and cloned into the TOPO entry vector (Invitrogen).

Protein tagged versions were generated using the primers listed in Table S1, cloned into TOPO vector (Invitrogen) for FLAG versions or pCR8 vector for HA version, and subcloned into the S2 cell expression vector pMT-DEST48 (Invitrogen) using the Gateway cloning system (Invitrogen).

Sbm GAL4 and UAS-Sbm cloning

To generate the Sbm-Gal4 transgenic line we analyzed the genome region of the *sbm* gene with Evoprinter (https://evoprinter.ninds.nih.gov/), comparing to the *D. sechellia*, *D. yakuba*, *D. erecta* and *D. Pseudoobscura* sequences and found that there were sections of the introns of the gene with a degree of conservation that suggested that they could contain regulatory sequences. We cloned into the TOPO vector (Invitrogen) an approximately 3.4 kb long fragment of the *locus* including the introns of the RC and RD transcripts, from the template BACR22I24 (Genebank ID AC022346, obtained from the BAC PAC Resources at the Children's Hospital Oakland

Research Institute) with the primers listed in Table S1. Using the Gateway system (Invitrogen) we subcloned this fragment into the pBPGAL4.2Uw-2 (a gift from Gerald Rubin, Addgene plasmid #26227, (Pfeiffer et al., 2010) and sent to Genetic Services, Inc. (MA, USA) for injection into their *y, w; attP2* line (location 68A4, chromosome 3L.)

To generate the UAS-Sbm transgenic line, we used the Gateway system (Invitrogen) to subclone the CDS from the TOPO vector to the pUASg:attB vector (Bischof et al., 2013) and sent to Genetic Services, Inc. (MA, USA) for injection into their *y, w; attP2* line (location 68A4, chromosome 3L.)

Sbm antibody

Polyclonal anti-Sbm antibody was produced by immunization of rabbits with the synthetic peptide EAPETDSSGTGRMRKPLE corresponding to amino acids 42-59 of Sbm-PA and PD, and amino acids 51-68 of Sbm-PB and PC (Eurogentec).

S2 cell immunocytology and western blots

Cells were seeded at 0.5 million cells/mL 30 h before transfection in multiwell plates with the same medium, then washed in Schneider's medium without FBS and transfected with FuGENE HD Transfection Reagent (Promega) in a ratio of 4.5:1 to sterile plasmid DNA (0.25 μg/μL). Cells were incubated for 17 h in the medium without FBS followed by 8 h in medium with 10% FBS and induced for 21 h with 0.5 mM copper sulfate.

For immunocytology cells were seeded on wells containing poly-L-lysine treated coverslips (0.01%, Sigma) before induction. After induction, they were washed three times with PBS and two times with PBS-Glycine 0.1M. Cells were permeabilized with Cytoperm (BD-Bioscience) and washed with PBS-Saponin 0.05%. Cells were incubated for 1 h with primary antibodies, then washed and further incubated with secondary antibodies for 30' and with Hoechst (1 μg/mL) for 5 minutes to mark nuclei. Cells were mounted in Fluorescent Mounting Medium (Dako). Images were obtained with a Leica TCS SP2 confocal microscope.

For western blots, transfected cells were scrapped, lysed 30 min at 4°C with 1% Triton X-100 buffer (150 mM NaCl; 50 mM Tris-HCl pH8; Triton X-100 1%; protease inhibitor Roche, 1 mM PMSF) and total proteins were quantified by Lowry method ("DC Protein Assay" kit from Biorad). Samples were then treated in Laemmli Buffer containing or not reducing agent (700 mM beta-mercaptoethanol; 200 mM DTT). Proteins were separated on polyacrylamide gel and then transferred on PVDF membranes. The membrane was probed with mouse anti HA antibody and then with the HRP conjugated secondary antibody, before being revealed with ECL reagent (Amersham biosciences).

Dissections and Immunostainings

Larval tissues were dissected in PBS and fixed in 4% paraformaldehyde (PFA) for 20 min at room temperature (RT), rinsed 3x15 min in PBS with 0.3% Triton X-100 (PT), and blocked with 7% normal goat serum (NGS; Sigma #G9023) for 45 min, followed by incubation with primary antibody at 4°C (overnight, or 48 h to 72 h). Samples were washed 4x15 min in PT, incubated with secondary antidobies for 2-3 h at RT, washed 4x15min in PT and mounted in Vectashield mounting medium. A list of the primary and secondary antibodies is listed in Table S2. For Phalloidin (Thermo Fisher Scientific) and Hoescht 33342 (Thermo Fisher Scientific) stainings a working solution was added together with, or 15 min immediately after secondary antibodies, respectively.

Fluorescence quantification

Images were taken on either a Leica TCS SP2, a Leica TCS SP8 or an LSM 880 Axio Imager 2 Zeiss confocal microscope. All quantitative analyses were performed employing FIJI Software. Within each experiment, all pictures were taken employing the same confocal settings and in the case of the Leica TCS SP8 Confocal microscope, using its Hybrid mode. Sbm signal in neurons, glia and gut, and somatic PTTH and somatic dilp2 levels were quantified from confocal images. In all cases, we analyzed the mean gray value, a measurement that is independent of the area.

For quantification of Sbm signal in glial cells, confocal images of brain hemispheres stained against Sbm and REPO were taken with a 20X objective and an optical zoom of 1.5X using a 1 μm step size. First, a Z projection of 6 stacks showing Sbm^+ glial signal was made. Then, a region of interest (ROI) was selected, adjusting the threshold image in order to mark most of the Sbm^+ glial signal. Mean Sbm fluorescence intensity and area was measured within the ROI created. A rectangle of the same or a higher area was located outside of glial membrane and used to subtract background signal. For the analysis, we considered the mean gray value (with the subtracted background signal) a measurement that is independent of the area. The same protocol was applied to measure Sbm levels in neuronal somas.

The mean gray value of PTTH in somas was quantified from confocal Z stacks of brains stained against Sbm and PTTH. Photos were obtained with a 20X objective with an optical zoom of 3X using a 0.69 μm step size. The signal of Sbm in neuronal somas was also quantified to compare its variation to that of PTTH. To compare the different genotypes and developmental times, PTTH values were normalized to the average PTTH signal of each independent experiment.

For dilp2 immunoreactive analysis within the IPCs, confocal images of brains stained against Dilp2 and FOXO (to better localize IPCs somas) were taken with a 40X objective and an optical zoom of 3 using a 0.69 μm step size. The mean gray value signal from Z stacks was measured as before, and data was normalized to the average dilp2 signal of each independent experiment to compare between genotypes.

Nuclei of glial cells (anti-Repo) and of dividing cells (anti-PH3) were counted using the Quantitation Module of Volocity® 6.3 software (Quorum Technologies Inc., Ontario), on LASX average Z projection images of one brain lobe, pre-treated with median filter.

In all cases of fluorescent signal quantification, the sample size is the number of brains measured, each brain is considered a replicate.

Developmental timing, growth assessment and feeding assays

Crosses were performed in oviposition chambers and eggs collected onto an agar plate supplemented with yeast paste for 4 hours. Recently hatched 1st-instar larvae were transferred to rearing vials (60 L1 larvae per vial). The number of pupa was counted and pupal volume was calculated as previously described (Galagovsky et al., 2014). Each mutant condition and its specific control were assayed strictly at the same time to allow their precise comparisons. Larval and male adult weight was measured with a Sartorius R160P balance. In the case of larvae, each replicate represents the average of 3 groups of 8 larvae. Virgin males were kept in groups of 15, left for 2 days at 25°C and frozen at −80°C until weighted. Wing size measurements were performed as in (Galagovsky et al., 2014). For feeding assays, larvae of the appropriate age were transferred to regular food tubes supplemented with 0.5% Brilliant Blue FCF (Sigma). After 2 h, larvae were recovered, thoroughly rinsed, dried and frozen at −80°C in groups of 8. The amount of food ingested was determined by absorbance measurement of FCF dye at 625 nm. Three groups of 8 larvae were averaged for each point.

Organ size measurement

Larvae were dissected and fixed in 0.4% PFA. Organs were dissected and mounted in 80% Glycerol in PBS, pictures taken with a Leica DM5000B microscope and measured using the FIJI software. Fat bodies were previously stained with Sudan Black (Sigma) following the protocol in (Tennessen et al., 2014). Brain lobe volume was estimated by approximating it to a sphere ($V = 4/3\pi(1/2W)^3$) where W is the average of the width of both brain lobes of a same brain.

Real Time PCRs

Total RNA for *dilp2*, *dilp5*, *dilp6* and *thor* analysis was prepared from 20 120 hAEL third-instar larval brains per sample. Larvae were dissected in PBS and brains immediately placed in Isol-RNA Lysis reagent (5-Prime) on ice and then lysed using a TissueLyser (QIAGEN) and frozen at −80°C until use. For *spookier* and *neverland* samples were prepared from 8 whole third instar larvae per sample, carefully synchronized at the second to third instar transition. For *sbm* 10 whole third instar larvae per sample were used. 4 to 7 independent samples were prepared for each point. In all cases *rpl29* was used to normalize. To determine copy number a standard curve was performed from a mix of equal amounts of all the samples in each experiment.

RNA was treated with DNAase (Thermo Fisher Scientific) and cDNA was synthesized using iScript cDNA synthesis kit (BioRad). Real Time PCRs were carried out using the IQ SYBR Green Supermix (BioRad) in a MyiQ single-color Real-Time PCR detection system (BioRad). Primers used are detailed in Table S1.

QUANTIFICATION AND STATISTICAL ANALYSIS

Statistical analyses were performed with the InfoStat package version 2009 (Grupo InfoStat, FCA, Universidad Nacional de Cordoba, Argentina). To determine if data met the criteria for ANOVA analysis normality was tested using Shapiro-Wilks test and the homogeneity of variance was assessed with Levene's test. $p < 0.05$ was considered statistically significant. In the cases were data did not meet these criteria, transformations were. If transformations were not sufficient to meet the criteria, non-parametric analysis were performed. For the analysis of pupation curves, each independent replicate, i.e., each vial with 60 initial larvae, was adjusted to a Gompertz non-linear regression model, and the median time to pupation was estimated. The statistical tests, number of replicates and special requirements for each experiment, as well as type of data transformation performed if necessary are detailed in the legends of the corresponding figures.

Cell Reports, Volume *24*

Supplemental Information

Sobremesa L-type Amino Acid Transporter Expressed in Glia Is Essential for Proper Timing of Development and Brain Growth

Diego Galagovsky, Ana Depetris-Chauvin, Gérard Manière, Flore Geillon, Martine Berthelot-Grosjean, Elodie Noirot, Georges Alves, and Yael Grosjean

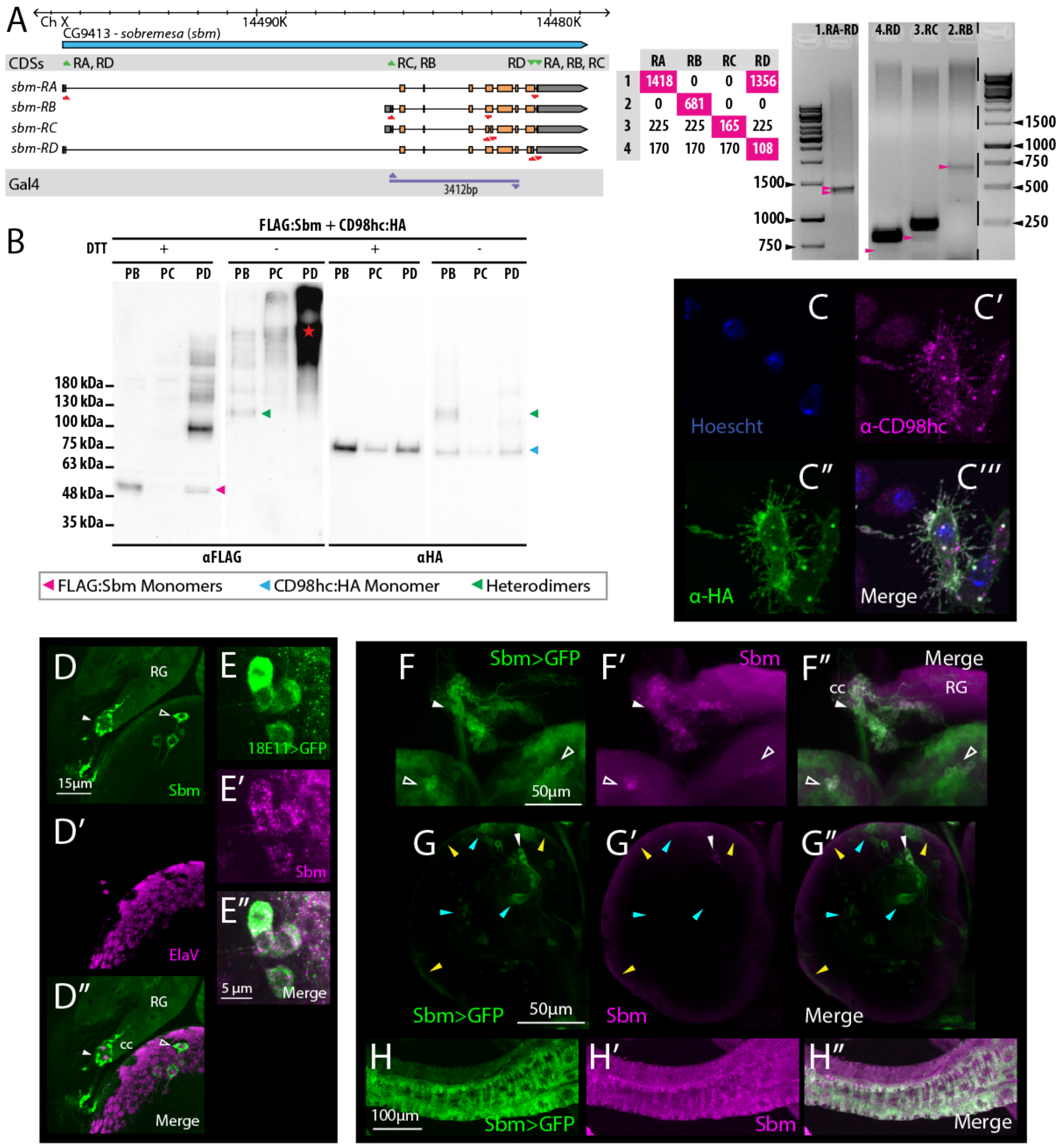

FIGURE S1. ***sobremesa*** **(CG9413 –** ***sbm*****) gene structure, products, expression pattern and localization. Related to Figure 1.** (A) Gene location and structure as reported in Flybase. We confirmed the existence of the four putative mRNAs. The two forward and two reverse primers used to amplify and clone the CDSs are shown in green on their target location relative to the mRNAs and the genomic *locus*. After cloning, colonies were screened using the pairs of primers

shown in red and selected according to the size of the amplification product obtained, as detailed on the table (magenta numbers indicate the unique bands used as diagnostic). Two gels showing diagnostic bands (magenta arrow heads) amplified by PCR from total cDNA are shown on the far right. In lilac is shown the region of the *locus* cloned and the primers used to generate the Sbm-Gal4 driver line. (B) Western Blot of extracts from S2 cells expressing FLAG:Sbm-PB, PC or PD together with CD98hc:HA in the presence (+) and absence (-) of reducing agent (DTT), revealed with anti-FLAG and anti-HA antibodies. The expected position of the heterodimer and monomer bands are pointed based on the molecular weight marker. The red star indicates signal saturation. For FLAG tagged Sbm-PB expected heterodimer bands are revealed in the absence of DTT and disappear or are fainter in the presence of DTT, while monomer bands behave in the opposite way. (C) The expression of CD98hc in S2 cells provokes membrane extensions. Pictures show in blue, Hoescht; magenta, CD98hc and green, HA. (D) Sbm antibody reveals expression in a group of cells in the brain (somas-hollow arrowhead, projections-filled arrowheads) projecting to the corpora cardiaca (cc) portion of the ring gland (RG). Staining against ElaV confirms that these cells are neurons. (E) The transgenic line 18E11-Gal4 drives expression in the Sbm^{+} neurons as revealed by expression of GFP (18E11>GFP in green) in the Sbm immunoreactive neurons (anti-Sbm in magenta.) (F-H) The Sbm-Gal4 transgenic line drives expression in the Sbm immunoreactive tissues and cells. (F) Sbm-Gal4 driven expression of GFP (green) can be observed in the somas (hollow arrowheads) and in the projections (filled arrowheads) of the neurons that express Sbm (magenta) and innervate the corpora cardiaca (cc) of the ring gland (RG). (G) Sbm-Gal4 drives expression of GFP (green) in the Sbm^{+} surface glia (magenta); yellow arrowheads point to spots of similar signal localization; a white arrowhead marks the Sbm^{+} neurons. Sbm-Gal4 also drives expression in many cells that do not show Sbm immunoreactive signal (examples are marked by cyan arrowhead.) (H) Sbm-Gal4 drives expression of GFP (green) in the region of the hindgut of the larva that shows immunoreactive Sbm signal (magenta.)

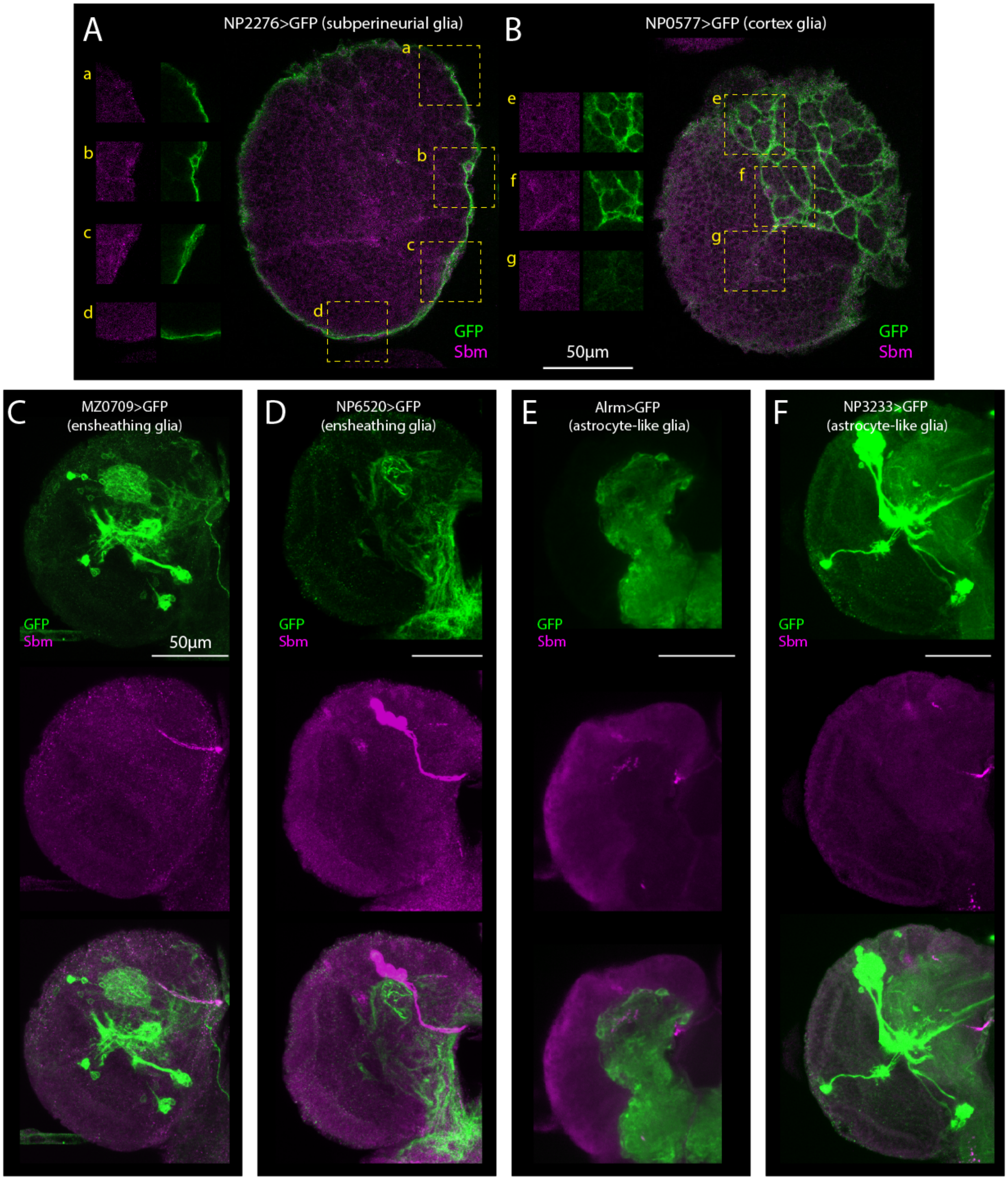

FIGURE S2. Sbm expression is observed in subperineurial and cortex glial subtypes, but not in other subtypes of glial cells. Related to Figure 1. (A-B) Subperineurial glia marked through the expression of GFP (green) driven by the NP2276-Gal4 line (A) and cortex glia marked through the expression of GFP (green) driven by the NP0577-Gal4 line (B), show expression of Sbm

(magenta). The merged signals are shown in the full frames. Squares of broken yellow line mark the position of the corresponding insets (a-g) which show details of the separate channels in A and B. (C-F) Expression of Sbm (magenta) is not observed overlapping other glial subtypes marked by the expression of GFP (green) driven by lines MZ0709-Gal4 (C) and NP6520-Gal4 (D) (ensheathing glia), and Alrm-Gal4 (E) and NP3233-Gal4 (F) (astrocyte-like glia). In all pictures the scale bar represents 50 µm. Pictures in A-B are single confocal planes. Pictures in C-F are Z-Stacks of single confocal planes.

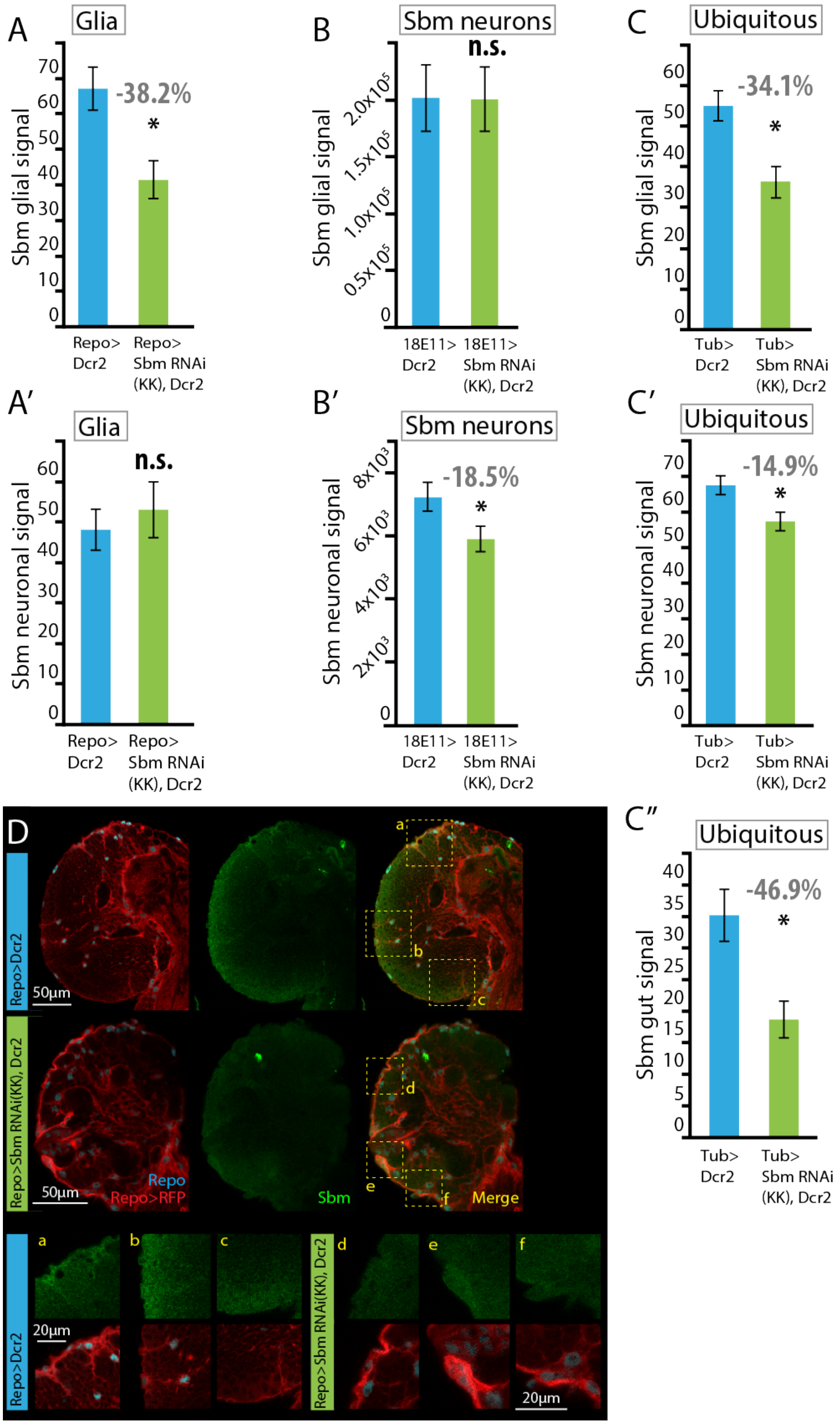
A
Glia
Sbm glial signal
-38.2%
*
Repo> Dcr2
Repo> Sbm RNAi (KK), Dcr2
B
Sbm neurons
n.s.
Sbm glial signal
18E11> Dcr2
18E11> Sbm RNAi (KK), Dcr2
C
Ubiquitous
Sbm glial signal
-34.1%
*
Tub> Dcr2
Tub> Sbm RNAi (KK), Dcr2
A'
Glia
n.s.
Sbm neuronal signal
Repo> Dcr2
Repo> Sbm RNAi (KK), Dcr2
B'
Sbm neurons
-18.5%
*
Sbm neuronal signal
18E11> Dcr2
18E11> Sbm RNAi (KK), Dcr2
C'
Ubiquitous
-14.9%
*
Sbm neuronal signal
Tub> Dcr2
Tub> Sbm RNAi (KK), Dcr2
D
Repo>Dcr2
Repo>Sbm RNAi(KK), Dcr2
50µm
Repo
Repo>RFP
Sbm
Merge
20µm
C"
Ubiquitous
-46.9%
*
Sbm gut signal
Tub> Dcr2
Tub> Sbm RNAi (KK), Dcr2

FIGURE S3. Expression of an RNAi against *sbm* reduces the levels of Sbm protein. Related to Figure 2. (A-A’) Expression of an RNAi against *sbm* in glia (Repo-Gal4) reduces the levels of Sbm protein in the glia (A) but not in the Sbm immunoreactive neurons (A’).(t-Test; t=-3.22 for A and t=0.62 for A’;*, p<0.05; error bars, S.E.M; n =22-24). (B-B’) Expression of an RNAi against *sbm* in the Sbm immunoreactive neurons (18E11-Gal4) does not reduce the levels of Sbm protein in glia (B) but significantly reduces the levels of Sbm protein in the Sbm immunoreactive neurons (B’) (t-Test; t=0.72 for B and t=2.94 for B’;*, p<0.05; error bars, S.E.M; n =32-30). (C-C’) Expression of an RNAi against *sbm* ubiquitously (Tub-Gal4) reduces the levels of Sbm protein in glia (C), in the Sbm immunoreactive neurons (C’) and in the gut (C’’) (t-Test; t=3.54 for C, t=2.72 for C’ and t=3.5 for C’’; *, p<0.05; error bars, S.E.M; n =20-22). (D) Pictures illustrating Sbm (green) levels in the glia marked by RFP (Repo>RFP) to show its membrane structure and with Repo antibody (cyan) to show the cell nuclei. The insets a-f show a detailed view of the Repo and Sbm signals in the corresponding regions marked in the Merge signal panels by yellow broken line squares. The RNAi expressing brains show diminished signal in the glial cells but the corresponding glial subtype membranes (marked by repo>RFP) is still visible.

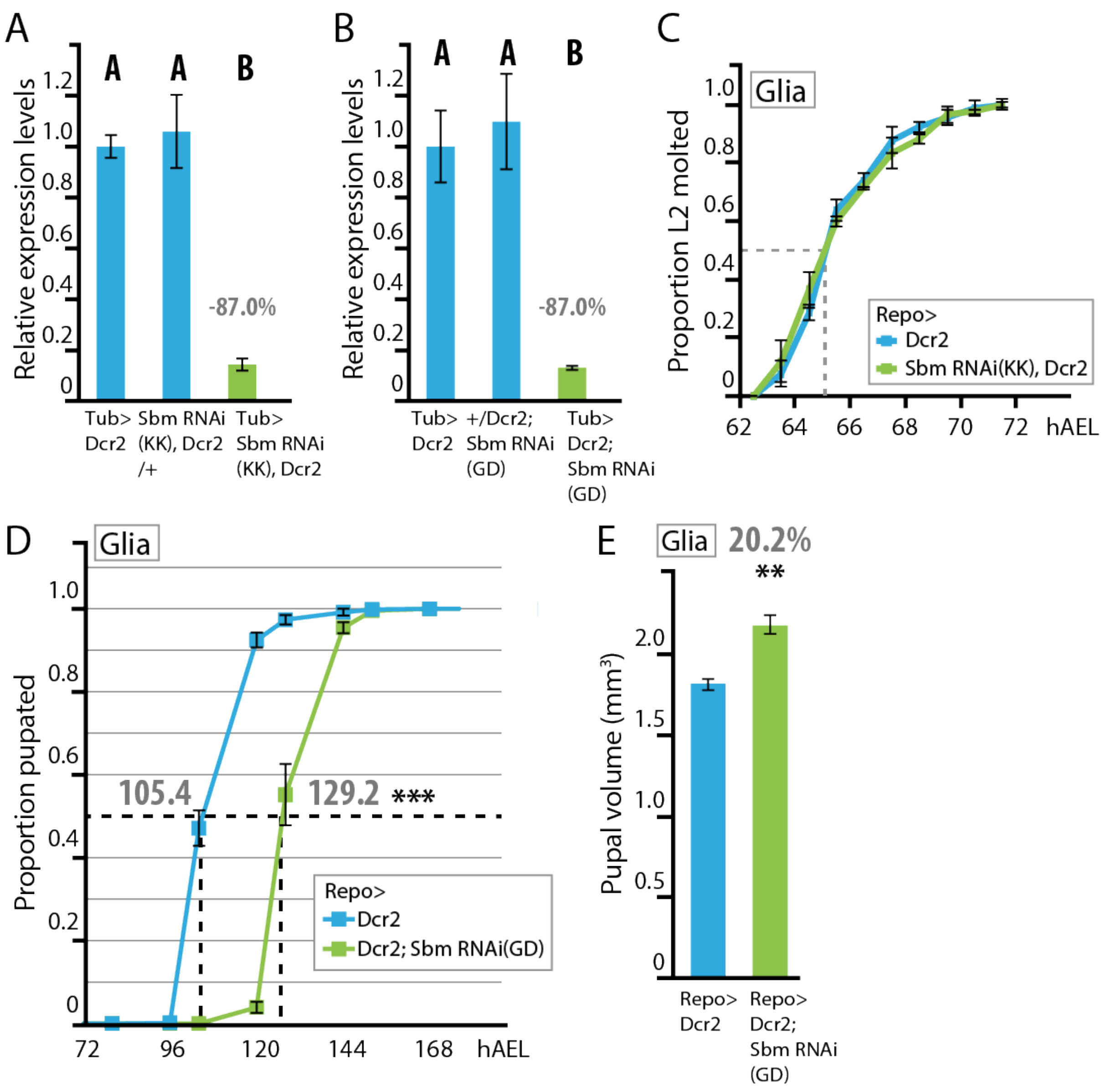

FIGURE S4. Expression of RNAi against *sbm* reduces the levels of *sbm* transcript. Related to Figure 2. (A-B) Quantitative RT-PCR showing that ubiquitous expression (Tub-Gal4) of (A) SbmRNAi (KK) or (B) SbmRNAi (GD), both combined with Dcr2, reduce the levels of *sbm* transcript relative to those of the controls (either Tub>Dcr2 or the heterozygous RNAis) (A, One-Way ANOVA, $p<0.0001$, $F=65.16$; bars with different letters signify that they are significantly different with $p<0.05$ in a Tukey post-test; error bars, S.E.M.; n=4-7; B, One-Way ANOVA, $p<0.0001$, $F=23.37$ with Tukey post-test; different letters, $p<0.05$; error bars, S.E.M.; n=4-5). **L2-L3 molt timing is not altered in the larvae expressing Sbm RNAi in glia.** (C)

Proportion through time of Repo>Dcr2 and Repo>SbmRNAi, Dcr2 second-instar larvae molting to the third instar shows no difference, demonstrating that the developmental delay caused by Sbm down-regulation is specific to the third-instar larval stage. **Effects of *sbm* downregulation in glia on developmental timing and growth, through the use of a second RNAi line.** (D-E) Proportion of larvae pupated through time (hAEL, hours after egg laying.) The expression of a second RNAi (SbmRNAi(GD), Dcr2) driven in glia by Repo-Gal4 leads to a 24 h delay to pupation (D), and an increase in pupal volume (E). (In D, t-Test, t=15.62; ***, p<0.0001; n=11-12, and in E, t-Test, t=5.93; **, p<0.0001.; n=7. Error bars, S.E.M.)

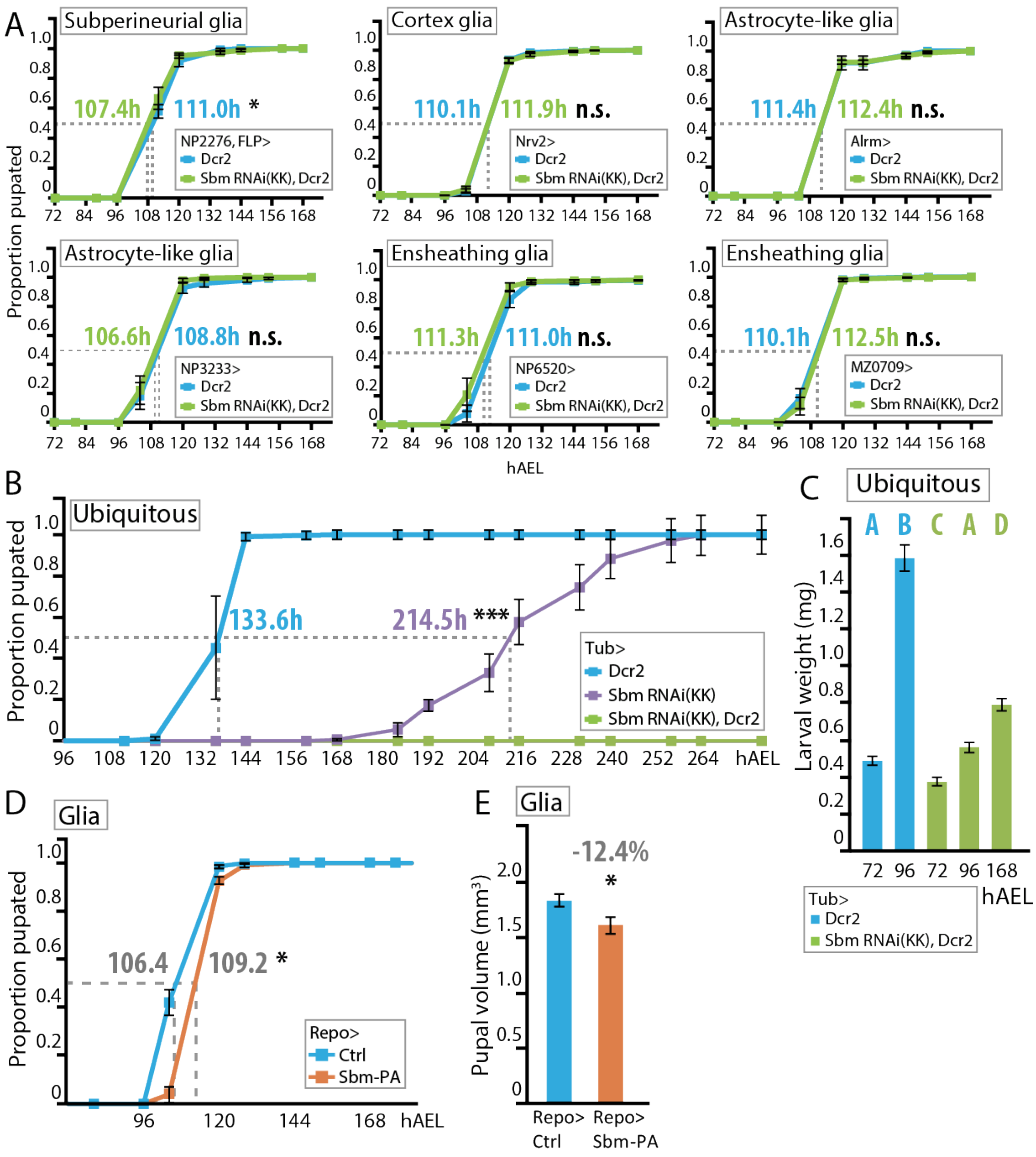

FIGURE S5. Downregulation of *sbm* in specific glial subtypes. Related to Figure 2. (A) Proportion of larvae pupated through time. In all cases the Gal4 driver is indicated on the top left corner of the graph and the glial subtype of expression on the bottom right. Blue lines and dots represent Dcr2 overexpression and green, Sbm RNAi combined with Dcr2. Subperineurial glial drivers were combined with UAS-FLP using the Act5C(FRT.CD2)GAL4 strain, to increase

expression by driving it with the strong *actin* gene promoter in the specific glial expression pattern (t-Test, from left to right and top to bottom t=2.52, t=1.44, t=0.38, t=0.68, t=0.17, t=1.29; n.s., non-significant differences; *, p=0.036; error bars, S.E.M.; n=5, n=10, n=3, n=3, n=4, n=10). **Effects of *sbm* ubiquitous downregulation on developmental timing and growth.** (B-C) Ubiquitous overexpression with the Tub-Gal4 driver of Dcr2 (blue in all graphs), SbmRNAi (purple) and both combined (green in all graphs) (B) Proportion of larvae pupated through time. (t-Test, t=9.18; *, p<0.0001; error bars, S.E.M.; n=5) (C) Larval weight at different developmental time points (Two-Way ANOVA, data was transformed to LN to fulfill the test criteria, p<0.0001, F=148.36; bars with different letters signify that they are significantly different with p<0.05 in a Duncan Post-Test; error bars, S.E.M.; n=5-7 per age and genotype). **Effects of *sbm* overexpression on developmental timing and growth.** (D-E) The expression of Sbm-PA driven in glia by Repo-Gal4 leads to a slight delay to pupation (D), and a significant decrease in pupal volume (E). (In D, t-Test, t=1.8; *, p<0.046; n=7, and in E, t-Test, t=2.32; **, p<0.05; error bars, S.E.M.; n=5. Error bars, S.E.M.) In all developmental curves, the mean median value in hours is indicated for each curve. hAEL, hours after egg laying.

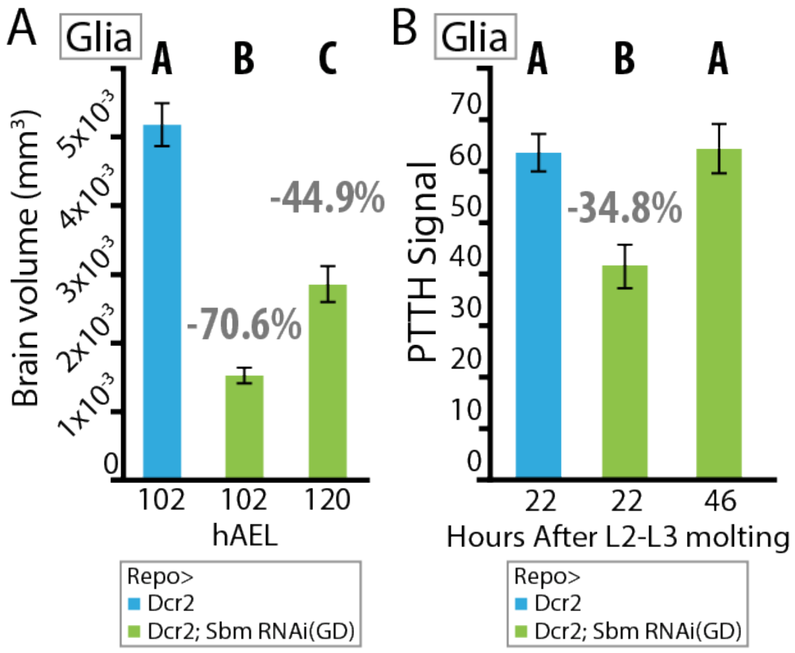

FIGURE S6 Effects of Sbm downregulation in glia on larval brain size and PTTH somatic levels using a second RNAi line. Related to Figure 3 and 6. (A) Expression of a second RNAi line combined with Dcr2 (Dcr2; SbmRNAi(GD)) also produces a reduction in brain size (estimated volume of a single brain lobe in mm^3) relative to controls (Dcr2) when expressed in glia (hAEL, hours after egg laying) (One-Way ANOVA, $p<0.0001$, $F=101.6$; bars with different letters signify that they are significantly different with $p<0.05$ in a Tukey post-test; different letters, $p<0.05$; error bars, S.E.M.; n=14-18). (B) PTTH levels in the somas of PTTH neurons at different time points in third-instar larvae synchronized at the second to third instar molt, upon downregulation of *sbm* in glia (Repo-Gal4) (Kruskal-Wallis, bars with different letters signify that they are significantly different, $p<0.05$; error bars, S.E.M.; n=38-40).

TABLE S1: list of all oligonucleotides used, related to the STAR METHODS (METHOD DETAILS, sbm transcript recognition, cloning and tagging, Sbm GAL4 and UAS-Sbm cloning)

Diagnostic primers for *sbm* transcripts		
RA-RD	F	CGCTTTCATTCTCTCGCAGT
RA-RD	R	GTGTAGCGCATCACGATCAG
RD	F	TATGTCCATGTGCGTCGTCT
RD	R	GTGTAGCGCATCACGATCAG
RC	F	CGACCATCTTGACCACACC
RC	R	TCTGGCATACGCTGAACTTG
RB	F	CCGCACTCCTTTCAGAACAT
RB	R	TTGAGATGGCTTCAACACCA

Cloning primers for *sbm* transcripts		
RB-RC	F	ATGCGCGATAAAATATCACAACT
RB-RC	R	GGGTATCCCCTTCACTCGAA
RA-RD	F	ATGTATCAGCATGTCCAACCA
RA-RD	R	GGGTATCCCCTTCACTCGAA
RD	F	ATGTATCAGCATGTCCAACCA
RD	R	GAGCAGGGCATAGAGATACTCA

Cloning primers for FLAG tagging		
FLAG-RA	F	CAAC**ATG**GACTACAAGGACGACGATGACAAGTATCAGCATGTCCAACCA
FLAG-RB	F	CAAC**ATG**GACTACAAGGACGACGATGACAAGCGCGATAAAATATCACAACT
FLAG-RC	F	CAAC**ATG**GACTACAAGGACGACGATGACAAGCGCGATAAAATATCACAACT
FLAG-RD	F	CAAC**ATG**GACTACAAGGACGACGATGACAAGTATCAGCATGTCCAACCA

Cloning primers for HA tagging		
CD98hc-HA	R	**TTA**AGCGTAATCTGGAACATCGTATGGGTA***TCCACCGCC***CTCCGCCTTCACTGGCA
CD98hc	F	CAACATGGTCCAGGATGAGAAGG

QPCR		
dilp2	F	ACGAGGTGCTGAGTATGGTGTGCG
dilp2	R	CACTTCGCAGCGGTTCCGATATCG
dilp5	F	TGTTCGCCAAACGAGGCACCTTGG
dilp5	R	CACGATTTGCGGCAACAGGAGTCG
dilp6	F	TGCTAGTCCTGGCCACCTTGTTCG
dilp6	R	GGAAATACATCGCCAAGGGCCACC
thor	F	TGATCACCAGGAAGGTTGTCATCTC

thor	R	GAGCCACGGAGATTCTTCATGAAAG
spok	F	TATCTCTTGGGCACACTCGCTG
spok	R	TATCTCTTGGGCACACTCGCTG
nvd	F	GGAAGCGTTGCTGACGACTGTG
nvd	R	TAAAGCCGTCCACTTCCTGCGA
sbm	F	GAATGACGCCGAGATTTATGA
sbm	R	ATGGCCATCGATGAGGTG
rpl29	F	GAACAAGAAGGCCCATCGTA
rpl29	R	AGTAAACAGGCTTTGGCTTGC
